# Nitrogen-containing FDA-approved drugs in 2025: synthesis, significance and therapeutic applications

**DOI:** 10.1039/d6ra00737f

**Published:** 2026-04-27

**Authors:** Chetna Jadala, Shweta Mishra, Gal Reddy Potuganti, Savio Cardoza, Ganga Reddy Velma

**Affiliations:** a Indiana University School of Medicine, Indiana University (IU) Indianapolis 46202 IN USA; b Dr. D.Y. Patil Institute of Pharmaceutical Sciences and Research Sant Tukaram Nagar, Pimpri Pune 411018 India; c DPGU - School of Pharmacy and Research Sant Tukaram Nagar, Pimpri Pune 411018 India; d Department of Chemistry and Biochemistry, The University of Oklahoma Norman Oklahoma 73019 USA; e Department of Pharmacology and Toxicology, R. Ken Coit College of Pharmacy, University of Arizona Tucson 85721 AZ USA vgreddy@arizona.edu velmagangareddy47@gmail.com

## Abstract

Nitrogen-containing heterocycles (N-Hets) are among the most prevalent and versatile structural motifs in pharmaceuticals, serving as key scaffolds in over 85% of biologically active small molecules. Their structural diversity and functional versatility have made them integral components of both U.S. Food and Drug Administration (FDA) approved and investigational drug molecules, modulating biological activity across a range of molecular targets. In 2025, the FDA approved 26 small-molecule drugs featuring N-Het frameworks, spanning multiple therapeutic areas, including oncology, metabolic disorders, infectious diseases, and rare/orphan conditions. This review offers a comprehensive overview of these newly approved compounds, emphasizing their biological activity and synthetic approaches. Special attention is given to drug–target interactions, focusing on receptor binding and highlighting the important role of N-Hets in medicinal chemistry. By exploiting the intrinsic chemical properties of N-Hets and leveraging modern synthetic methodologies, these scaffolds continue to drive the discovery of therapeutically relevant molecules, highlighting their sustained significance in modern drug discovery.

## Abstract 2

Nitrogen-containing heterocycles (N-Hets) are among the most prevalent and versatile structural motifs in pharmaceuticals, serving as key scaffolds in over 85% of biologically active small molecules.

## Graphical abstract

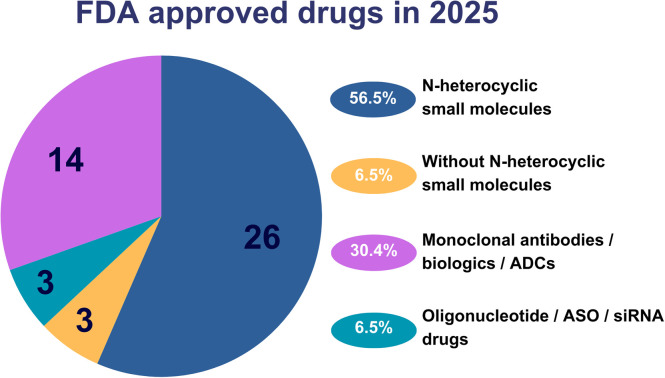

## 1. Introduction

Heterocyclic compounds, in general, constitute an important class of naturally occurring molecules with favorable physicochemical and biological properties, conferring substantial pharmaceutical relevance. Their adaptable structures serve as biomimetic frameworks, often incorporating essential pharmacophoric elements, making them indispensable motifs in modern drug discovery.^1–3^ Among the diverse heterocyclic systems,^4^ nitrogen-containing heterocycles (N-Hets) are particularly valued for their structural diversity, chemical stability, and ability to modulate biological targets with high specificity.^5,6^ N-Het represents one of the most prominent structural motifs in pharmaceuticals and remains a key fragment in drug discovery.^7^ Their versatile frameworks provide structural flexibility, enabling fine-tuning of the pharmacokinetic properties and biological efficacy of both investigational and approved drug molecules.^8^ Over the past two decades, these scaffolds have attracted extensive research, driving the design of novel small molecules and enabling the development of innovative synthetic strategies. Beyond advancing organic synthesis, N-Hets have played a central role in the discovery and development of therapeutically relevant compounds.^9–12^

This prominent feature of N-Het is further supported by a comprehensive database analysis of approved pharmaceuticals, underscoring their widespread presence in clinically successful therapeutics. The N-heterocyclic skeletons span a broad range of therapeutic applications, including anticancer,^13–16^ anti-HIV,^17^ anti-inflammatory,^18^ anti-convulsant,^19^ anti-diabetic,^13^ anti-bacterial^20^ and anti-depressants.^21^ They serve as key building blocks for numerous drug candidates, largely owing to the ability of nitrogen atoms to readily participate in hydrogen-bonding interactions with biological targets, thereby enhancing target recognition and binding affinity. Among the diverse heterocyclic frameworks, triazoles,^22^ tetrazoles,^23^ imidazoles,^24^ benzimidazoles,^25^ pyrimidines,^26,27^ and quinolines^26^ have received particular attention, emerging as highly prevalent scaffolds contributing to a wide range of therapeutic applications across diverse disease areas.

**Fig. 1:**
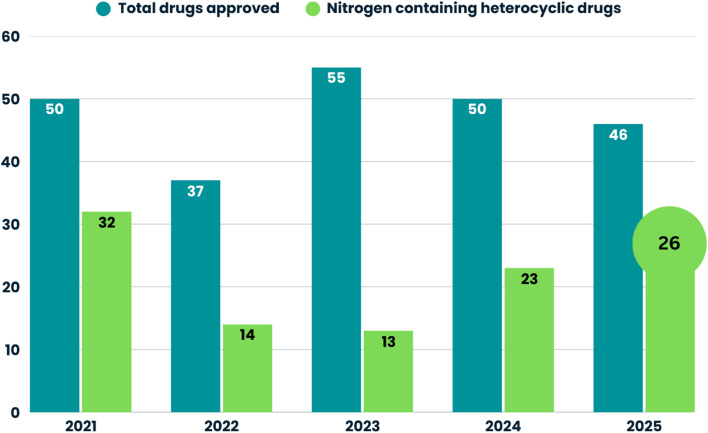
Trends in FDA drug approvals and nitrogen-containing heterocyclic small molecules from 2021 to 2025.

**Fig. 2:**
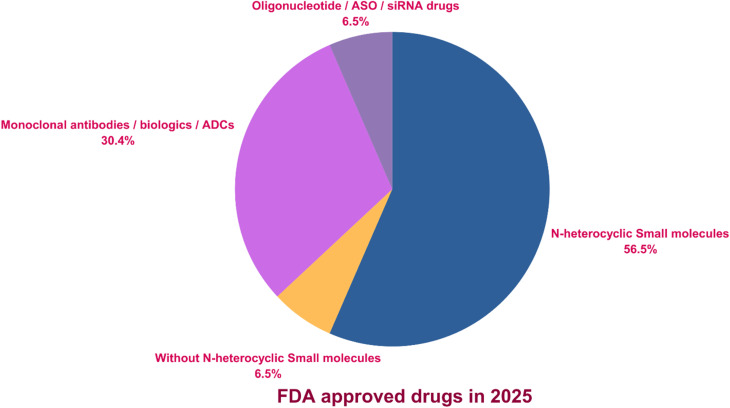
Contribution of N-heterocyclic small molecules to FDA-approved drugs in 2025.

A review of FDA-approved small molecules from 2021 to 2025 reveals a variable trend in N-Het approvals (Fig. 1). In 2021, 32 of 50 small molecules (64%) contained N-heterocyclic scaffolds. Approval declined in 2022 and 2023, with 14 of 37 (37%) and 13 of 55 (23%) N-heterocyclic small molecules, respectively. This trend reversed in 2024 and 2025, with approvals increasing to 23 of 50 (46%) and 26 of 46 (56%), respectively, highlighting the sustained relevance of N-Het in medicinal chemistry and drug discovery. In 2025, the FDA approved 46 drugs, of which 29 were small molecules.^28^ Among these, N-Het drugs accounted for 56.5% of approvals, reflecting their predominance among small molecules. Monoclonal antibodies, biologics, and antibody–drug conjugates (ADCs) together accounted for 30.4%, while oligonucleotide- and siRNA-based therapies accounted for the remaining 6.5% (Fig. 2).

**Fig. 3:**
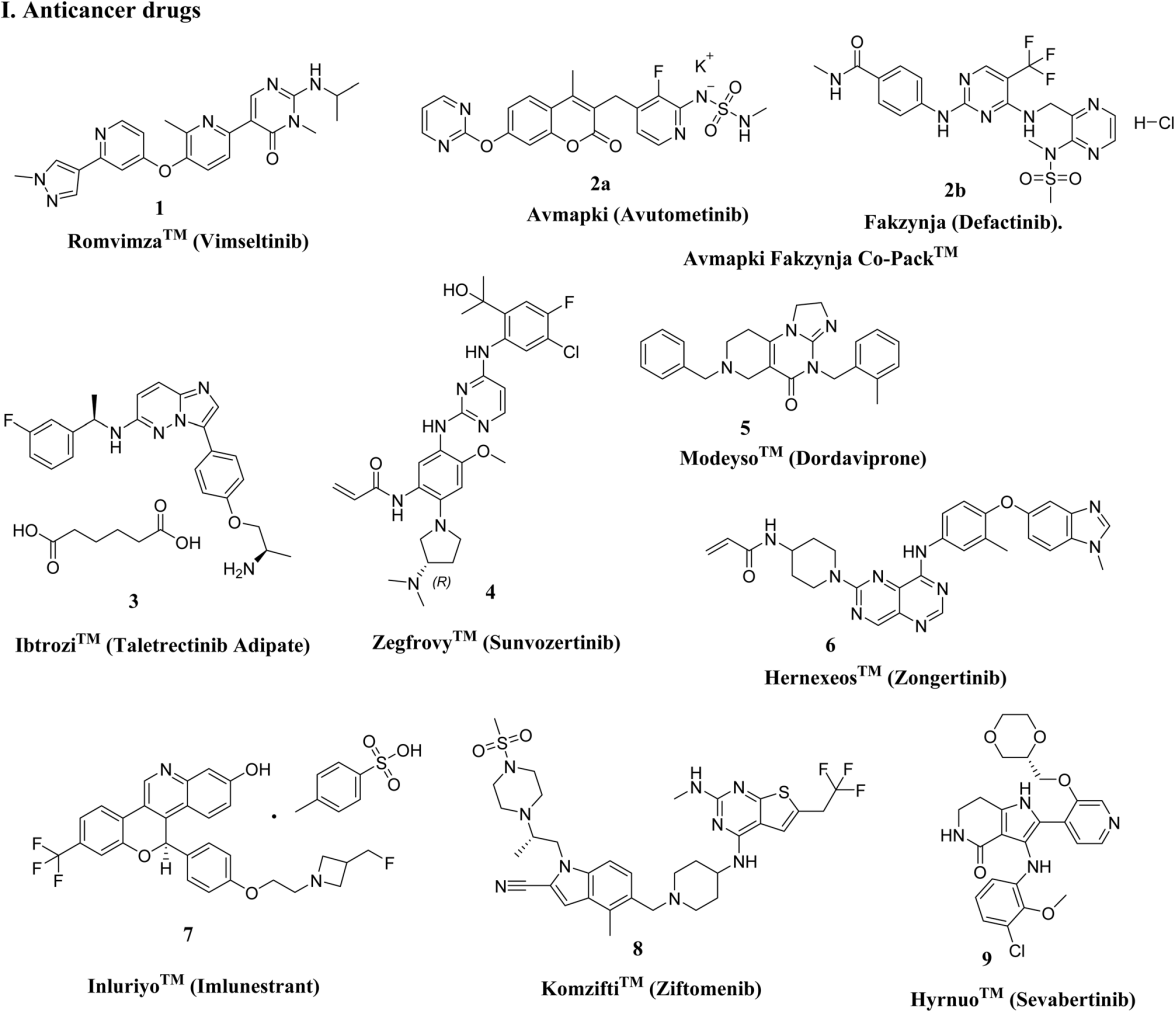
Nitrogen-containing anticancer drugs approved by the FDA in 2025.

**Fig. 4:**
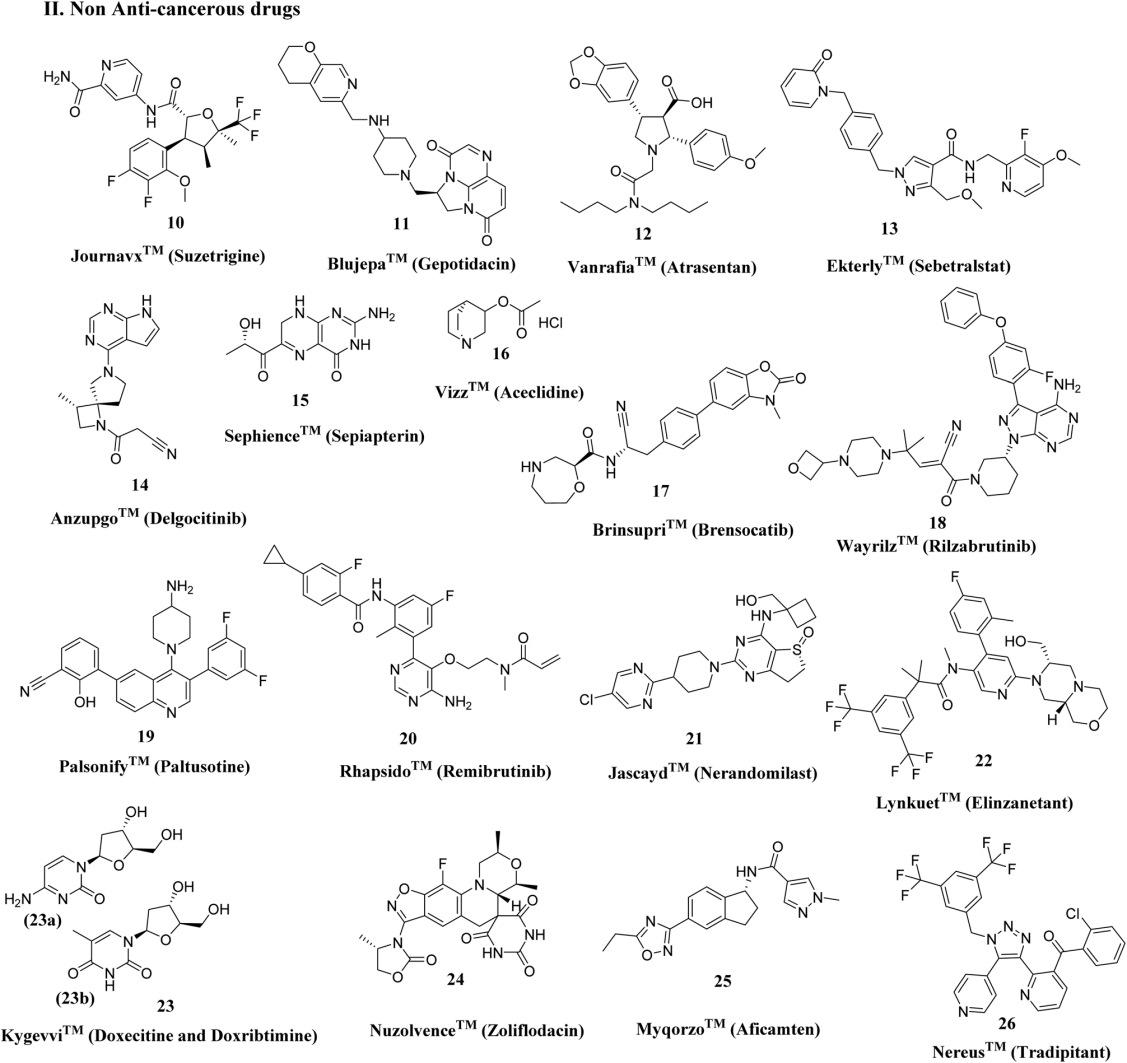
Nitrogen-containing non-anticancerous drugs approved by the FDA in 2025 across different therapeutic areas.

In 2025, FDA approvals spanned a broad range of therapeutic areas, reflecting ongoing efforts to address unmet medical needs in oncology, rare genetic disorders, metabolic diseases, infectious diseases, and cardiovascular conditions.^15,29,30^ Notably, 16 drugs (∼35%) targeted various cancers, including 9 N-Het small molecules (Fig. 3). Key approvals include Romvimza™ (vimseltinib) for symptomatic tenosynovial giant cell tumor (TGCT); Avmapki Fakzynja Co-Pack™ for KRAS-mutated recurrent low-grade serous ovarian cancer (LGSOC); Komzifti™ (ziftomenib) for relapsed or refractory acute leukemia; and Hyrnuo™ (sevabertinib) for HER2-mutated non-small cell lung cancer, highlighting ongoing advances in targeted oncology therapies and the continued prominence of N-Het scaffolds. Beyond oncology, approvals addressed genetic, rare, and metabolic disorders (Fig. 4), such as Kygevvi™ (doxecitine and doxribtimine) for thymidine kinase 2 deficiency, Sephience™ (sepiapterin) for hyperphenylalaninemia in phenylketonuria, and Palsonify™ (paltusotine) for adult acromegaly. Cardiovascular innovations included Myqorzo™ (aficamten) for obstructive hypertrophic cardiomyopathy. Infectious disease approvals included novel oral antibiotics Blujepa™ (gepotidacin) and Nuzolvence™ (zoliflodacin) for urinary tract infections and uncomplicated gonorrhea, reflecting efforts against antimicrobial-resistant pathogens.

Additional approvals encompassed lung diseases, including non-cystic fibrosis bronchiectasis (NCFB) with Brinsupri™ (brensocatib) and idiopathic pulmonary fibrosis (IPF) with Jascayd™ (nerandomilast), rare blood disorders were addressed by Wayrilz™ (rilzabrutinib), while endocrine and menopausal symptoms were treated with Lynkuet™ (elinzanetant), ophthalmic conditions such as presbyopia were managed by Vizz™, (aceclidine). Pain management included Journavx™ (suzetrigine), dermatological conditions encompassed chronic hand eczema with Anzupgo™ (delgocitinib), and for chronic spontaneous urticaria with Rhapsido™ (remibrutinib), and Nereus™ (tradipitant) was approved for motion sickness. Importantly, 2025 approvals emphasized treatments for rare and orphan diseases, including Ekterly™ (sebetralstat), Vanrafia™ (atrasentan), and Romvimza™ (vimseltinib) *etc.*, highlighting a strategic focus on addressing unmet needs in less common conditions.

This review focuses on the synthetic strategies and clinical applications of FDA-approved nitrogen-containing heterocycles in FDA-approved drugs in 2025, with the aim of providing insights into their mechanisms of action and therapeutic potential. To facilitate review and interpretation, nitrogen-containing heterocyclic molecules are classified into anticancer and non-anticancer categories. We further examine the synthetic pathways used in their development, emphasizing innovative approaches that could guide future drug design efforts.

In light of the increasing complexity of modern drug discovery, the efficient and sustainable synthesis of these molecules remains a critical component for advancing pharmaceutical research and accelerating the translation of novel therapies to the clinic. In this context, understanding the structure–activity relationships and pharmacophoric features of nitrogen-containing scaffolds is essential.^31^

### 1.1 Pharmacophore features, rational design, and SAR considerations

Although the FDA-approved drugs discussed in this review are structurally diverse, several general structure–activity relationship (SAR) trends can be identified for nitrogen-containing scaffolds. The presence and placement of nitrogen atoms significantly impact target binding through hydrogen bonds and ionic interactions, thus influencing potency and selectivity. Aromatic nitrogen heterocycles often contribute to π–π stacking and electronic modulation, while aliphatic amines enhance solubility and pharmacokinetic properties.^32^ Additionally, substitution patterns on heterocyclic frameworks are crucial for enhancing biological activity and target specificity.

Nitrogen-containing heterocycles play a central role in shaping the pharmacophoric structure of modern drugs. Incorporating nitrogen atoms as amines, amides, and within heterocyclic frameworks enables key interactions with biological targets, such as hydrogen bonding and ionic interactions, thereby improving binding affinity and selectivity. In many relevant clinical situations, protonated nitrogen centers help facilitate electrostatic interactions with negatively charged residues in enzyme active sites or receptor binding pockets.^33^

From a rational design perspective, nitrogen atoms are incorporated to adjust physicochemical properties such as p*K*_a_, aqueous solubility, and membrane permeability. Aromatic nitrogen-containing heterocycles like pyridine, imidazole, triazole, and pyrimidine serve as adaptable scaffolds for modifying electronic properties and enhancing metabolic stability. Conversely, saturated nitrogen-containing motifs such as piperidine and morpholine are often added to increase solubility and improve pharmacokinetic profiles. These design principles are evident in many FDA-approved drugs discussed in this review, where nitrogen-containing pharmacophores enhance target specificity and therapeutic effectiveness. The dominance of nitrogen-containing frameworks among FDA-approved drugs in 2025 further emphasizes their importance in balancing potency, selectivity, and favorable pharmacokinetic properties.^34^

## 2. FDA-approved nitrogen-containing drugs in 2025

### 2.1 Anticancer drugs

#### 2.1.1 Romvimza™ (vimseltinib)

Romvimza™ (vimseltinib, 1), developed by Deciphera Pharmaceuticals, LLC, is an orally bioavailable, small molecule kinase inhibitor that selectively targets the colony-stimulating factor 1 receptor (CSF1R).^35^ It has been approved for the treatment of adult patients with symptomatic TGCT for whom surgical resection is not feasible or is expected to result in significant functional impairment or severe morbidity. TGCT is a rare, non-malignant proliferative disorder of the synovial lining driven by aberrant CSF1-CSF1R signaling, leading to synovial hyperplasia, joint destruction, pain, and restricted mobility. Vimseltinib received U.S. FDA approval on February 14, 2025, based on results from the Phase III MOTION trial, a double-blind, multicenter, randomized, placebo-controlled study in which 123 patients with symptomatic TGCT not amenable to surgical resection were randomized (2 : 1) to receive either vimseltinib or placebo. Approval of vimseltinib underscores the successful translation of selective CSF1R inhibition from molecular design to clinical efficacy. Vimseltinib acts as a selective CSF1R inhibitor by binding to the kinase switch-control region and stabilizing the receptor in an inactive conformation (PDB ID: 7MFC). At the molecular level, the inhibitor forms key hydrogen-bonding interactions with residues such as Asp-796 and Cys-712 within the active site. Additional interactions with residues, including Lys-662, further contribute to stabilization of the drug–target complex. These interactions collectively promote the DFG-out conformation and enhance binding affinity and selectivity.^36^ Limitations of the drug: treatment may cause elevated Aspartate Aminotransferase (AST)/Alanine Aminotransferase (ALT) and fetal risk, so liver tests and contraception are advised.

**Scheme 1:**
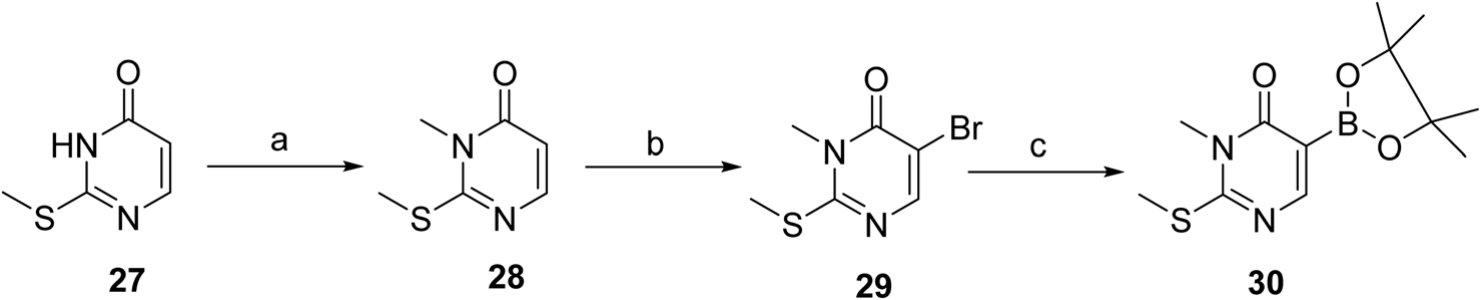
Synthesis of intermediate 30. ^*a*^Reaction conditions: (a) LiHMDS, MeI, DMF (62%); (b) Br_2_, DCM (97%); (c) bis(pinacolato)diboron, KOAc, PdCl_2_(dppf), DCM, 1,4-dioxane, 85 °C (100%).

**Scheme 2:**
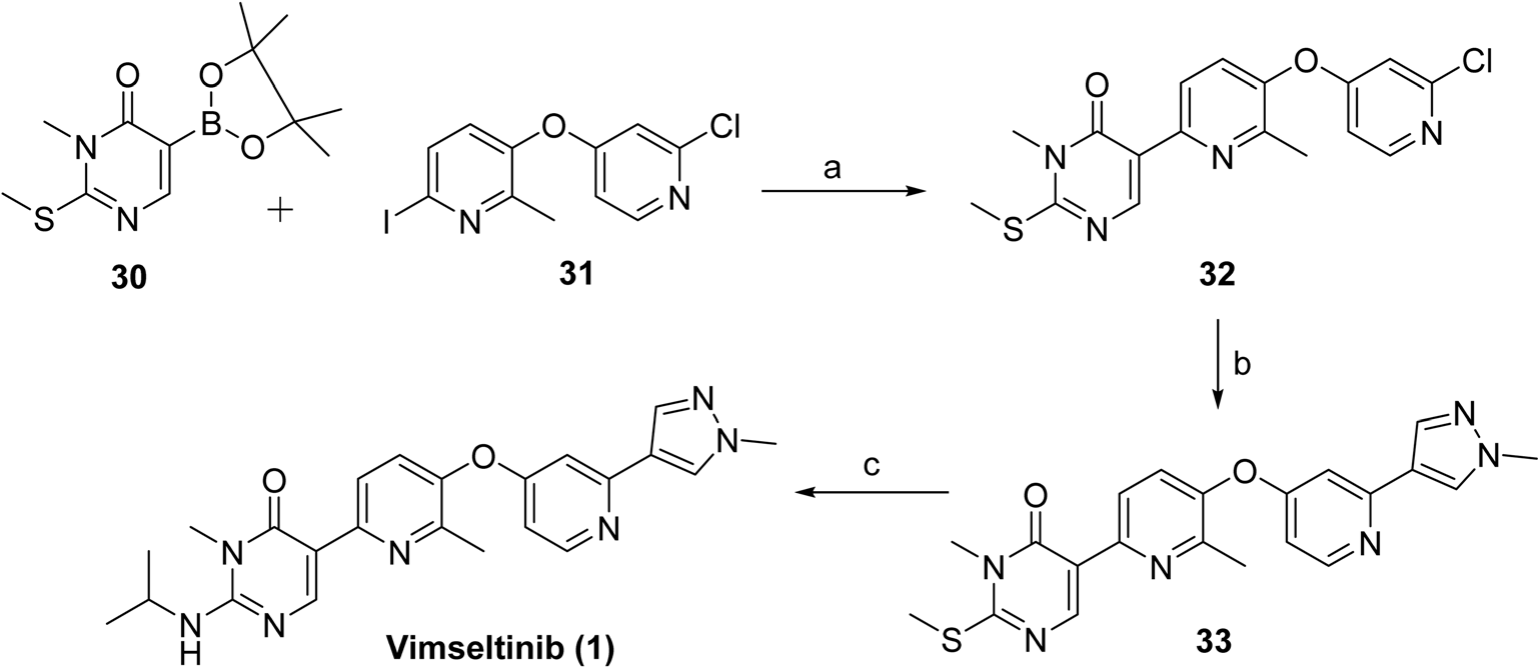
Synthesis of vimseltinib (1). ^*a*^Reaction conditions: (a) K_2_CO_3_, Pd(PPh_3_)_4_, 1,4-dioxane, H_2_O, 90 °C (67%); (b) 1-methyl-4-(4,4,5,5-tetramethyl-1,3,2-dioxaborolan-2-yl)-1*H*-pyrazole, K_2_CO_3_, Pd(PPh_3_)_4_, 1,4-dioxane, H_2_O, 90 °C (34%); (c) ^i^PrNH_2_ (neat), 100 °C (59%).

Schemes 1 and 2 outlines the synthetic route used for the preparation of vimseltinib (1).^35^ Methylation of the commercially available pyrimidone 27, using iodomethane and LiHMDS afforded intermediate 28, which was subsequently brominated to give 29. Intermediate 29 was then converted to corresponding boronate ester 30*via* palladium-mediated cross-coupling reaction with bis(pinacolato)diboron. Suzuki–Miyaura cross-coupling of 30 with the 2-chloropyridine derivative 31 provided the intermediate 32, which was further coupled with the appropriately substituted pyrazole boronate derivative, under classical Suzuki-type coupling conditions, to afford the pyrimidone 33. Final amination of 33 with isopropylamine provided vimseltinib (1).

#### 2.1.2 Avmapki Fakzynja Co-Pack™ (avutometinib and defactinib)

Avmapki Fakzynja Co-Pack™ (2), developed by Verastem, Inc., is an orally administered combination of avutometinib and defactinib approved for the treatment of adult patients with KRAS-mutated recurrent LGSOC who have received prior systemic therapy. Avmapki (Avutometinib, 2a) is a protein kinase inhibitor for the Raf and MEK mitogen-activated protein kinases (MAPKs) that suppresses cancer growth by shutting down the MAPK pathway and reducing drug resistance by preventing pathway reactivation, while Fakzynja (defactinib, 2b) is a focal adhesion kinase (FAK) inhibitor. Together, these agents provide a more comprehensive blockade of tumor growth and resistance mechanisms in RAS/MAPK pathway-dependent tumors.^37^

LGSOC^38^ is a rare, slow-growing, and highly recurrent ovarian cancer that predominantly affects younger women and shows limited responsiveness to standard platinum-based chemotherapy, approximately 30% of cases harbor KRAS mutations. On May 8, 2025, Avmapki Fakzynja Co-Pack (avutometinib in combination with defactinib) became the first FDA-approved therapy for KRAS-mutated recurrent LGSOC, receiving accelerated approval based on results from the Phase 2 RAMP 201 trial (NCT04625270), which demonstrated a 44% overall response rate and a duration ranging from 3.3 to 31.1 months. Prior to this approval, no FDA-approved treatments were available for LGSOC, which is a biologically distinct subtype that differs from high-grade serous ovarian cancer in both disease pathology and therapeutic response. The combination was granted breakthrough therapy and orphan drug designations. Avutometinib functions as a RAF/MEK pathway inhibitor by modulating kinase signaling and stabilizing the inactive conformation of MEK, thereby preventing downstream MAPK activation. Defactinib acts as an ATP-competitive inhibitor of focal adhesion kinase (FAK), binding within the catalytic domain and disrupting integrin-mediated signaling pathways involved in tumor cell survival and migration. These complementary mechanisms enable dual-pathway inhibition and contribute to enhanced antitumor efficacy. A limitation of this therapy is that approval was granted under the accelerated approval pathway based on response rate, and continued approval is contingent upon confirmation of clinical benefit in a confirmatory trial.

**Scheme 3:**
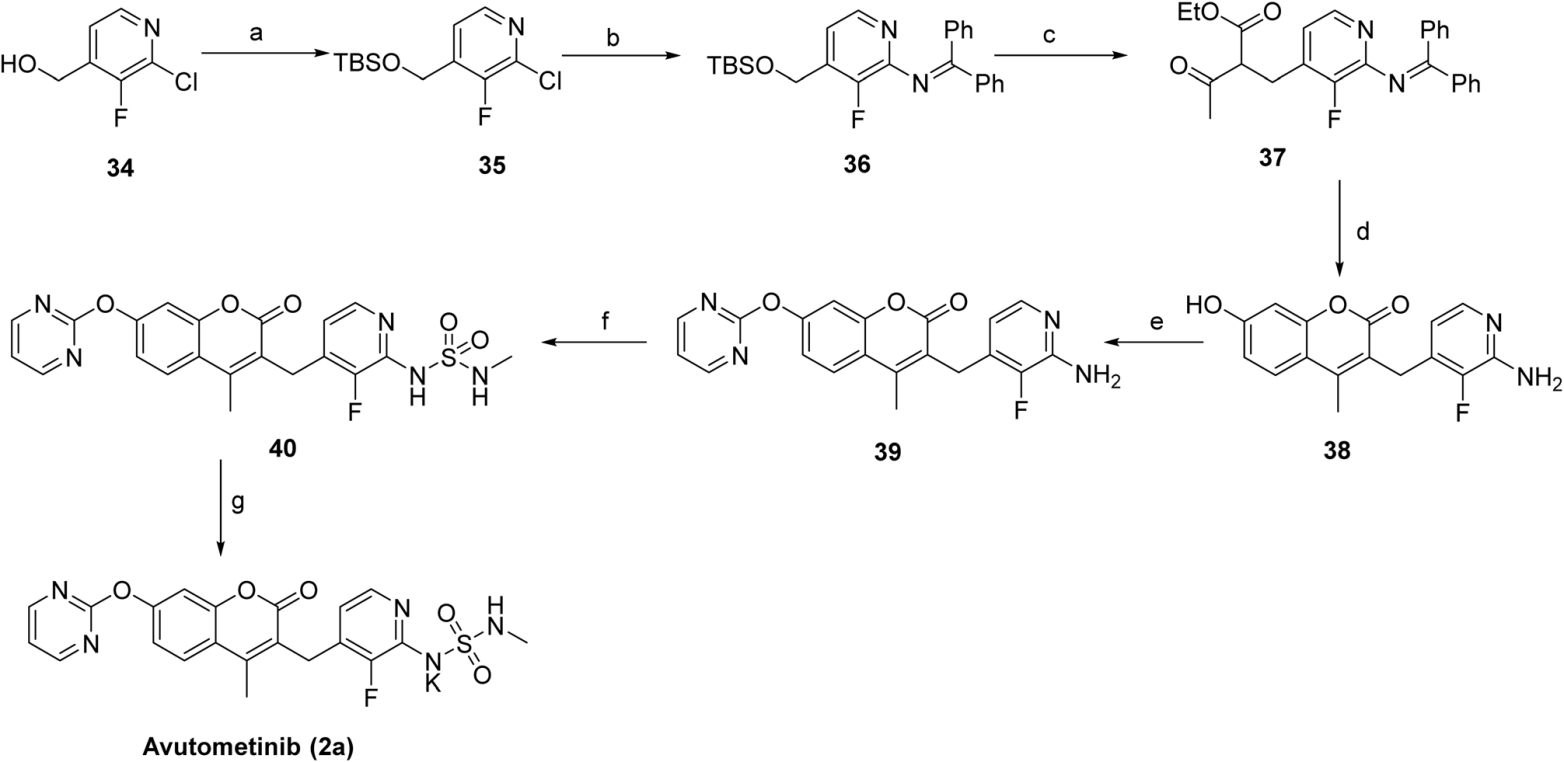
Synthesis of avutometinib (2a). ^*a*^Reaction conditions: (a) *tert*-butyldimethylsilyl chloride, imidazole, DMF; (b) benzophenone imine, Pd_2_(dba)_3_, rac-BINAP, *t*-BuONa, toluene, 60 °C; (c) ethyl 3-oxobutanoate, *t*-BuOLi, NaI, THF, 50 °C, 3 h; (d) resorcinol, H_2_SO_4_; (e) 2-bromopyrimidine, NaH, DMF; (f) *N*-methylsulfamoyl chloride, pyridine, DMF; (g) KOH.

Scheme 3 outlines the synthesis of avutometinib (2a).^39^ The route begins with the protection of the hydroxy group in 34 using *tert*-butyldimethylsilyl chloride, affording the protected intermediate 35, followed by palladium-catalyzed C–N coupling with benzophenone imine to give 36. C–C bond formation with ethyl 3-oxobutanoate generated 37, which then underwent acid-mediated cyclization with resorcinol to furnish 38. The pyrimidine moiety was installed *via* nucleophilic substitution with 2-bromopyrimidine, producing 39, and the sulfonamide functionality was introduced by reaction with *N*-methylsulfamoyl chloride to give 40. Final deprotection and/or saponification using KOH afforded the target compound, avutometinib (2a).

**Scheme 4:**
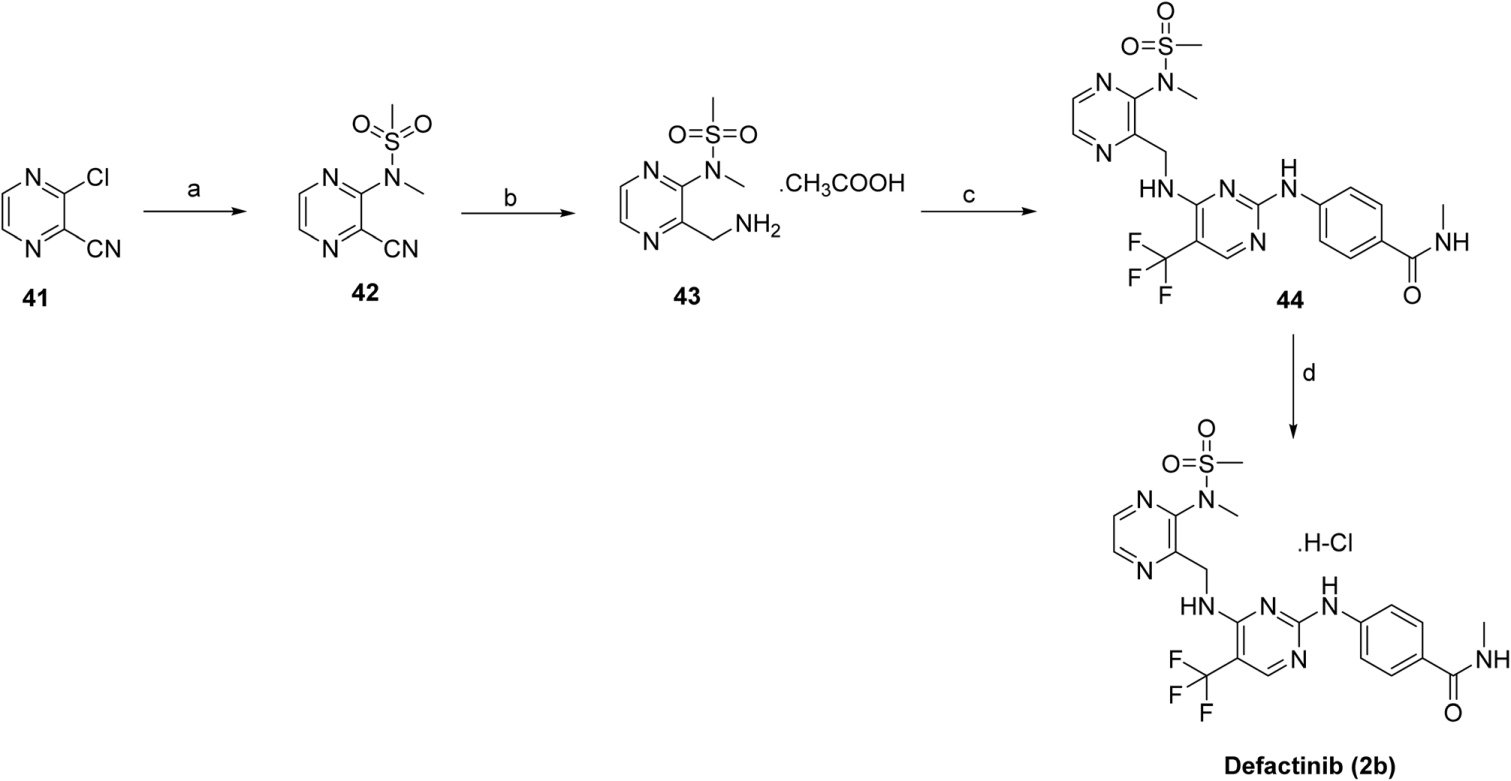
Synthesis of defactinib (2b). ^*a*^Reaction conditions: (a) *N*-methyl-methanesulfonamide; Cs_2_CO_3_; (b) H_2_, Pd/C in methanolic ammonia, AcOH in EtOAc; (c) 4-((4-chloro-5-(trifluoromethyl)pyrimidin-2-yl)amino)-*N*-methylbenzamide, DIEA in DCE/t-BuOH; (d) HCl in MeOH.

The target compound defactinib (2b)^40^ was synthesized as shown in Scheme 4. The synthesis began with the introduction of the *N*-methylsulfonamide group on 41 using *N*-methyl-methanesulfonamide in the presence of Cs_2_CO_3_, affording intermediate 42. The resulting intermediate 42 was then reduced with H_2_ and Pd/C to provide 43. Subsequent coupling of 43 with 4-((4-chloro-5-(trifluoromethyl)pyrimidin-2-yl)amino)-*N*-methylbenzamide in the presence of DIEA in DCE/*t*-BuOH installed the substituted pyrimidine amide functionality. Finally, treatment with HCl in MeOH resulted in deprotection, yielding the desired compound, defactinib (2b).

#### 2.1.3 Ibtrozi™ (taletrectinib)

Ibtrozi™ (taletrectinib, 3), developed by Nuvation Bio Inc. is an orally administered, small molecule next-generation ROS1 tyrosine kinase inhibitor (TKI) with central nervous system activity.^41,42^ Taletrectinib has demonstrated high and durable clinical responses, including robust activity against intracranial disease and resistance-associated mutations such as G2032R, while maintaining a favorable safety profile with a low incidence of neurologic adverse events.^43^ On June 11, 2025, the U.S. FDA approved taletrectinib for the treatment of adults with locally advanced or metastatic ROS1-positive non-small cell lung cancer (NSCLC). The drug is marketed under the brand name Ibtrozi and is administered as the taletrectinib adipate salt. FDA approval was based on efficacy and safety data from 270 patients with ROS1-positive NSCLC who had progressed beyond the lungs and who received taletrectinib at a dose of 600 mg orally once daily. These patients were enrolled in two multicenter, single-arm, open-label clinical trials, TRUST-I (NCT04395677) and TRUST-II (NCT04919811), which demonstrated the clinical activity of taletrectinib in this patient population.^43^ Taletrectinib acts as an ATP-competitive ROS1 kinase inhibitor by binding to the ATP-binding pocket and blocking kinase activity. This inhibition disrupts downstream signaling pathways, including MAPK and PI3K/AKT, thereby suppressing tumor growth and survival. Taletrectinib received priority review, breakthrough therapy, and orphan drug designations based on its clinical efficacy; however, its prescribing information includes warnings and precautions for hepatotoxicity, interstitial lung disease/pneumonitis, QTc interval prolongation, hyperuricemia, myalgia associated with creatine phosphokinase elevation, skeletal fractures, and embryo-fetal toxicity, which represent important limitations to its clinical use.

**Scheme 5:**
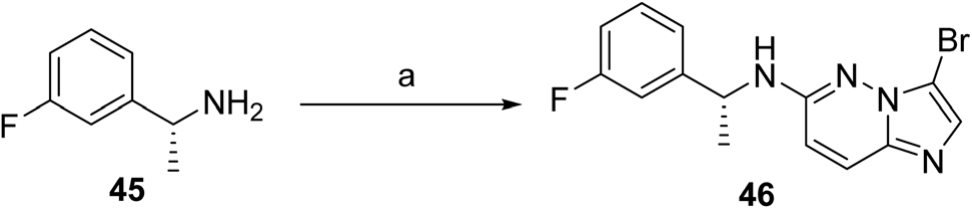
Synthesis of intermediate (46). ^*a*^Reaction conditions: (a) 3-bromo-6-chloroimidazo[1,2-*b*]pyridazine KF, DMSO at 120 °C.

**Scheme 6:**
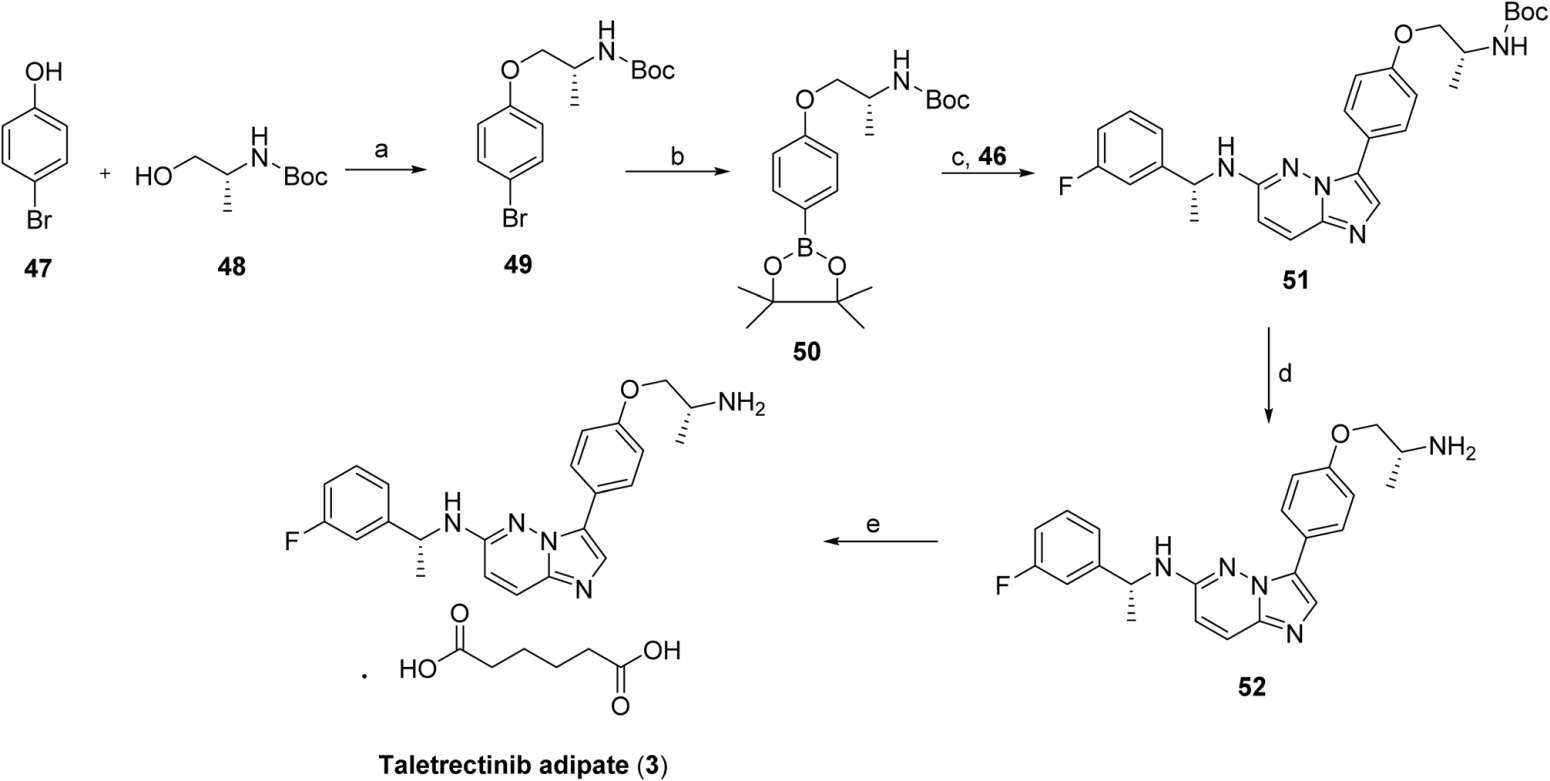
Synthesis of taletrectinib (3). ^*a*^Reaction conditions: (a) *N*-Boc-d-alaninol PPh_3_ and DIAD THF; (b) bis(pinacolato)diboron, using PdCl_2_(dppf)·CH_2_Cl_2_ and KOAc, 1,4-dioxane; (c) intermediate 46, PdCl_2_(dppf)·CH_2_Cl_2_ and K_2_CO_3_, 1,4-dioxane/H_2_O; (d) HCl in MeOH/1,4-dioxane; (e) adipic acid in *n*-PrOH.

The synthesis of taletrectinib (3)^44^ begins with a Mitsunobu coupling between *p*-bromophenol (47) and *N*-Boc-d-alaninol (48) using PPh_3_ and DIAD in THF, affording *N*-Boc-1-(4-bromophenoxy)-2(*R*)-propanamine (49) as shown in Scheme 6. This intermediate is then subjected to Miyaura borylation with bis(pinacolato)diboron in the presence of PdCl_2_(dppf)·CH_2_Cl_2_ and KOAc in 1,4-dioxane at 80 °C to yield the corresponding boronate ester (50). In a parallel sequence, nucleophilic aromatic substitution between 1(*R*)-(3-fluorophenyl)ethanamine (45) and 3-bromo-6-chloroimidazo[1,2-*b*]pyridazine, conducted in the presence of KF in DMSO at 120 °C, furnishes the secondary amine intermediate (46), as shown in Scheme 5. This intermediate is subsequently coupled with boronate (50) *via* a Suzuki–Miyaura cross-coupling using PdCl_2_(dppf)·CH_2_Cl_2_ and K_2_CO_3_ in refluxing 1,4-dioxane/water to afford *N*-Boc-protected taletrectinib (51). Final carbamate deprotection using HCl in methanol/1,4-dioxane yields taletrectinib free base (52), which is then converted to the final product by salt formation with adipic acid in *n*-propanol affording taletrectinib adipate (3).

#### 2.1.4 Zegfrovy™ (sunvozertinib)

Zegfrovy™ (sunvozertinib, 4), developed by Dizal (Jiangsu) Pharmaceutical Co., Ltd., is an orally administered, potent, irreversible, and mutant-selective epidermal growth factor receptor (EGFR) tyrosine kinase inhibitor (TKI) that shows activity against EGFR exon 20 insertion mutations (exon20ins) and other mutations.^45^ On July 2, 2025, the U.S. FDA granted accelerated approval to sunvozertinib (Zegfrovy) for the treatment of adult patients with locally advanced or metastatic non-small cell lung cancer (NSCLC) harboring EGFR exon 20 insertion mutations, as detected by an FDA-approved test, whose disease has progressed on or after platinum-based chemotherapy. The approval was based on efficacy data from WU-KONG1B (NCT03974022), a multinational, open-label, dose-randomization clinical trial enrolling patients with locally advanced or metastatic EGFR exon20ins-positive NSCLC following progression on platinum-based chemotherapy.^46,47^ This indication was granted accelerated approval based on overall response rate and duration of response, with continued approval contingent upon confirmation of clinical benefit in ongoing or future confirmatory trials. Mechanistically, sunvozertinib acts as a mutant-selective EGFR tyrosine kinase inhibitor by binding to the ATP-binding pocket of the receptor, thereby inhibiting kinase activity and downstream signaling pathways such as MAPK and PI3K/AKT.^46^ The prescribing information for sunvozertinib includes warnings for interstitial lung disease/pneumonitis, gastrointestinal and dermatologic adverse reactions, ocular toxicity, and embryo-fetal toxicity, which represent key limitations to its clinical use.

**Scheme 7:**
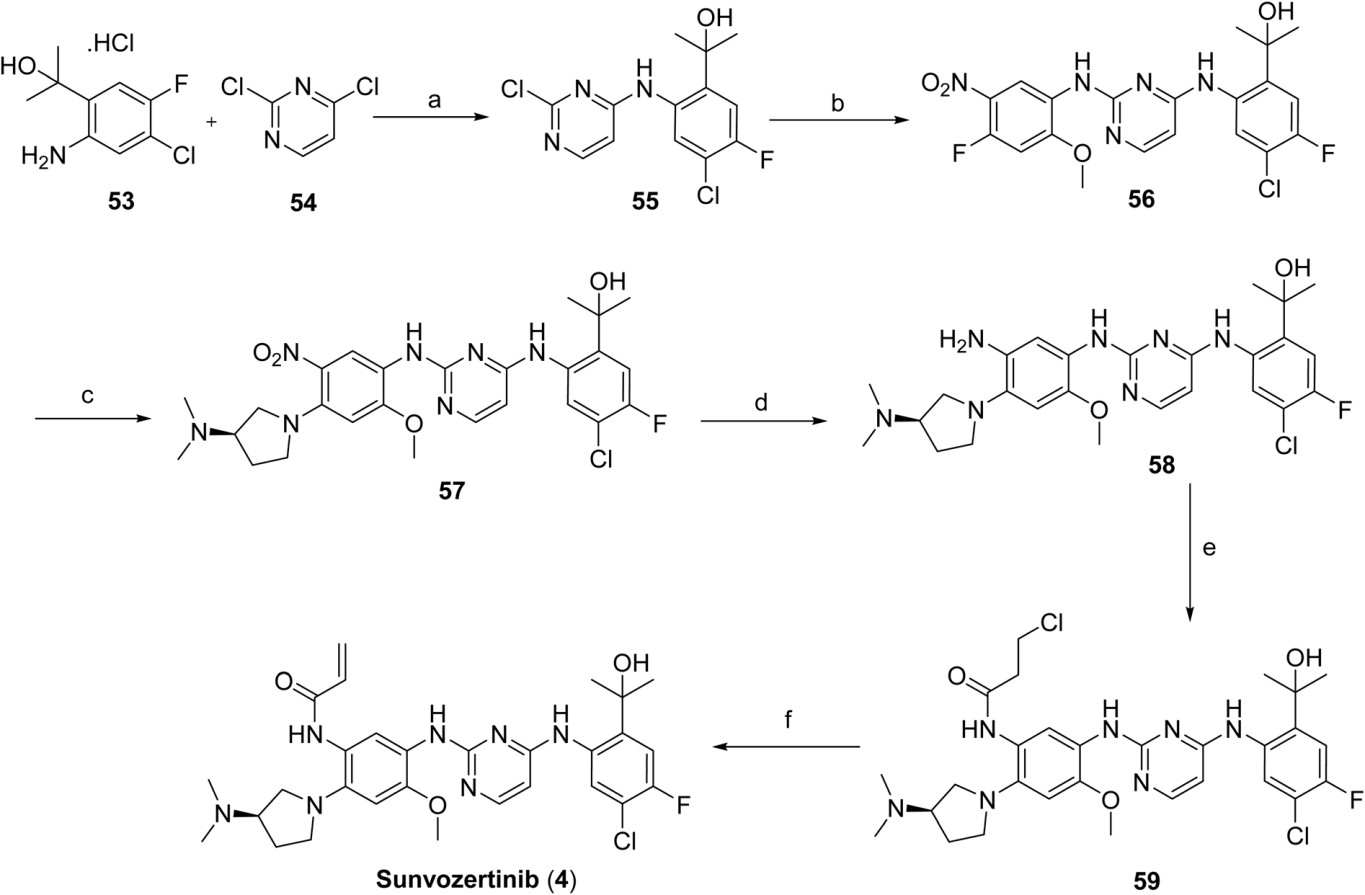
Synthesis of sunvozertinib (4). ^*a*^Reaction conditions: (a) DIPEA, IPA; (b) 4-fluoro-2-methoxy-5-nitroaniline, IPA, TFA; (c) (*R*)-*N*,*N*-dimethylpyrrolidin-3-amine, DIPEA, K_2_CO_3_, ACN; (d) Pt/C, THF; (e) acyl chloride, THF, H_2_O; (f) NaOH.

The synthesis of sunvozertinib (4) is depicted in Scheme 7. Compounds 53 and 54 undergo nucleophilic substitution under alkaline conditions to afford intermediate 55, which reacts with 4-fluoro-2-methoxy-5-nitroaniline to yield intermediate 56. Subsequent nucleophilic substitution of 56 with (*R*)-*N*,*N*-dimethylpyrrolidin-3-amine provides intermediate 57. Reduction of the nitro group in 57 using hydrogen and platinum on carbon furnishes the corresponding amino intermediate 58. Condensation of 58 with an acyl chloride generates amide 59, which undergoes base-mediated elimination to afford the final product, sunvozertinib (4).^48^

#### 2.1.5 Modeyso™ (dordaviprone)

Modeyso™ (dordaviprone, 5), developed by Jazz Pharmaceuticals, Inc. is an orally administered anticancer agent that functions as a dopamine D2 receptor antagonist and an allosteric activator of the mitochondrial caseinolytic protease P (ClpP).^49^ It is developed for the treatment of diffuse midline glioma, a highly aggressive and infiltrative grade IV brain tumor that is difficult to remove surgically because of its diffuse nature. Dordaviprone is a first-in-class small molecule imipridone. On August 6, 2025, the U.S. FDA granted accelerated approval to Modeyso™ (dordaviprone) for adult and pediatric patients aged 1 year and older with H3 K27M-mutant diffuse midline glioma whose disease has progressed following prior therapy, marking the first systemic therapy approved for this patient population.^50^ This approval was based on an integrated analysis of 50 patients across five open-label clinical trials, which demonstrated an overall response rate of 22% and a median duration of response of 10.3 months. Although these results are promising, confirmatory studies are required, and the ongoing phase 3 ACTION trial (NCT05580562) is evaluating dordaviprone in newly diagnosed patients following radiotherapy. Together, these findings highlight the clinical potential of dordaviprone and the urgent need for effective systemic therapies for patients with H3 K27M-mutant diffuse midline glioma. Dordaviprone received Orphan Drug, Rare Pediatric Disease, and Fast Track designations and was approved under accelerated approval based on response rate and duration of response, pending confirmatory evidence of clinical benefit. Dordaviprone functions through dual targeting of dopamine D2 receptors and mitochondrial ClpP, triggering mitochondrial stress and activation of the integrated stress response, ultimately promoting tumor cell apoptosis.^49^ Its use is limited by risks of hypersensitivity, QTc prolongation, and embryo-fetal toxicity.

**Scheme 8:**
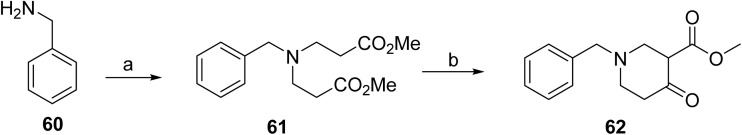
Synthesis of intermediate (62). ^*a*^Reaction conditions: (a) methyl acrylate, MeOH; (b) NaH, THF.

**Scheme 9:**
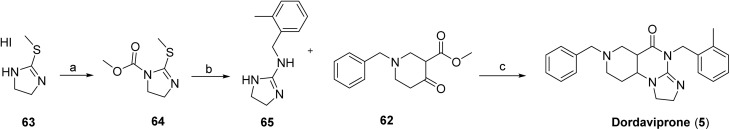
Synthesis of dordaviprone (5). ^*a*^Reaction conditions: (a) methyl chloroformate, Et_3_N,CH_2_Cl_2_; (b) 2-methylbenzylamine, MeOH/AcOH, reflux; (c) NaOMe, MeOH, reflux.

The synthesis of dordaviprone (5)^51^ begins with the formation of intermediate 64 from commercially available 2-methylthio-2-imidazoline hydroiodide (63) through reaction with methyl chloroformate as shown in Scheme 9. Intermediate 64 is then coupled with 2-methylbenzylamine to generate the corresponding guanidine derivative 65. In parallel, 1-benzyl-4-oxopiperidine-3-carboxylate (62) is prepared from benzylamine (60) and methyl acrylate *via* dimethyl 3,3′-(benzylazanediyl)dipropionate (61), which is subsequently treated with sodium hydride in THF to yield 62 as shown in Scheme 8. The final step involves condensation of 65 with 62 under reflux in methanol in the presence of sodium methoxide, affording dordaviprone (5).

#### 2.1.6 Hernexeos™ (zongertinib)

Hernexeos™ (zongertinib, 6) developed by Boehringer Ingelheim Pharmaceuticals, Inc., is an orally administered anticancer agent and a selective kinase inhibitor targeting human epidermal growth factor receptor 2 (HER2).^52^ It is indicated for the treatment of adults with unresectable or metastatic non-squamous non-small cell lung cancer (NSCLC) whose tumors harbor activating mutations in the HER2 (ERBB2) tyrosine kinase domain.^53,54^ The U.S. FDA granted zongertinib priority review, breakthrough therapy, and fast track designations in recognition of its potential clinical benefit. On August 8, 2025, the FDA granted accelerated approval to zongertinib for adults with unresectable or metastatic non-squamous NSCLC with HER2 (ERBB2) tyrosine kinase domain-activating mutations, as detected by an FDA-approved test, who had received prior systemic therapy. The approval was based on efficacy data from the BEAMION LUNG-1 trial (NCT04886804), an open-label, multicenter, multi-cohort study evaluating zongertinib in patients with previously treated, unresectable or metastatic HER2-mutant non-squamous NSCLC. This indication received accelerated approval based on response rate and duration of response, with continued approval dependent on confirmation of clinical benefit in a confirmatory trial. Zongertinib acts as a selective HER2 tyrosine kinase inhibitor by binding to the ATP-binding pocket of the receptor and inhibiting its catalytic activity. This interaction suppresses downstream signaling pathways, including MAPK and PI3K/AKT, thereby inhibiting tumor cell proliferation.^52^ Key safety warnings include hepatotoxicity, left ventricular dysfunction, interstitial lung disease/pneumonitis, and embryo-fetal toxicity.

**Scheme 10:**
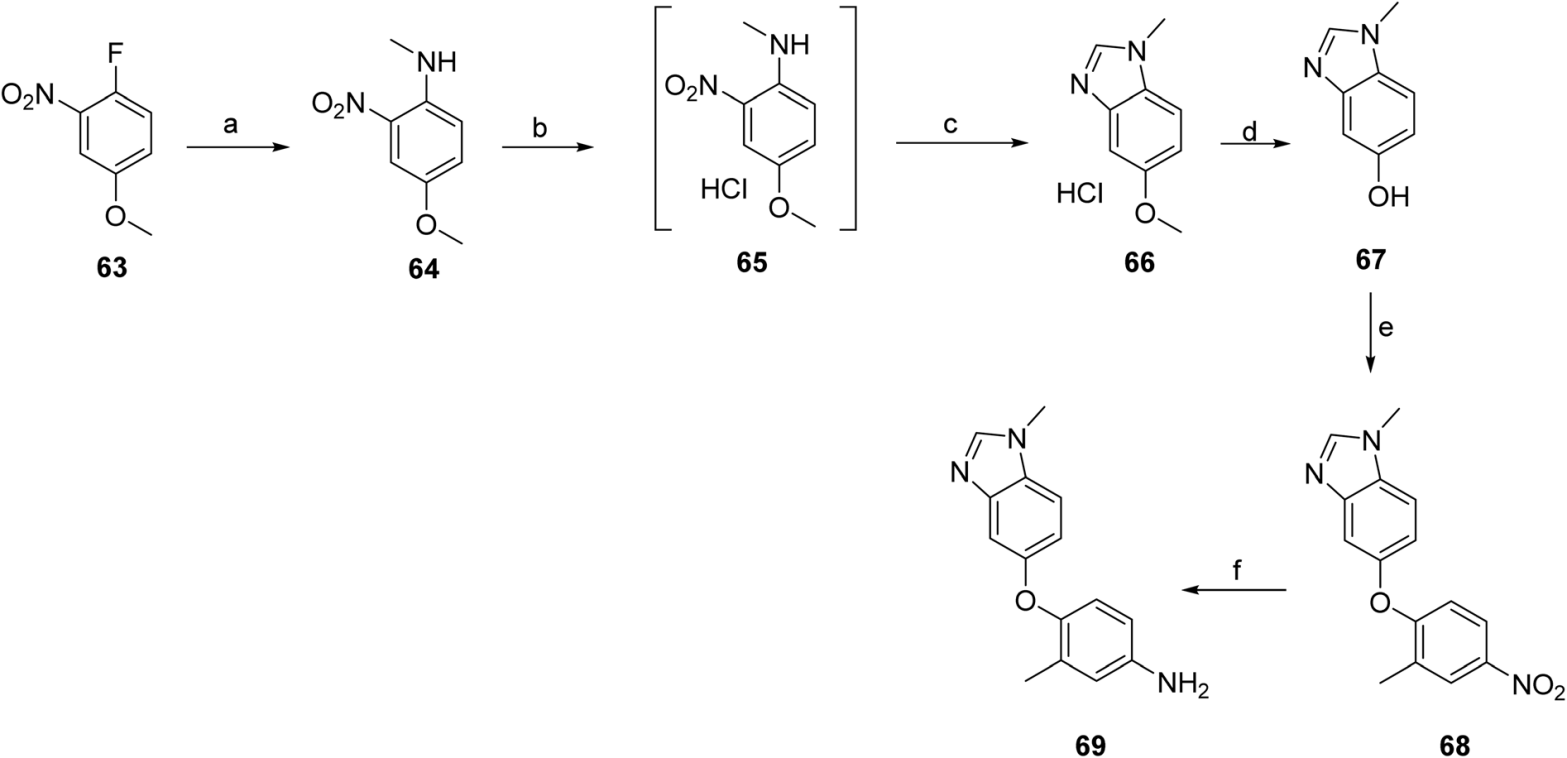
Synthesis of intermediate (69). ^*a*^Reaction conditions: (a) MeNH_2_, K_2_CO_3_, EtOH, 50 °C; (b) (i) Pd/C, H_2_, MeOH, 50 °C, (ii) HCl; (c) methyl formate, MeOH, 41 °C; (d) (i) HBr, 100 °C, (ii) NaOH, H_2_O; (e) 1-fluoro-2-methyl-4-nitrobenzene, K_2_CO_3_, DMAc, 85 °C; (f) Pd/C, H_2_, MeOH, 25 °C.

The synthesis for zongertinib proceeds *via* the initial preparation of intermediate 69. The Intermediate 69 was synthesized from starting material 63*via* a nucleophilic aromatic substitution (SNAr) with methylamine, affording intermediate 64 as shown in Scheme 10. Catalytic hydrogenation of 64 yielded the corresponding 65, which was subsequently cyclized with methyl formate to construct the benzimidazole core, giving intermediate 66. Demethylation of 66 produced compound 67, which then underwent a further S_N_Ar reaction to furnish intermediate 68. Final hydrogenation under mild conditions provided compound 69 after purification by crystallization.

**Scheme 11:**
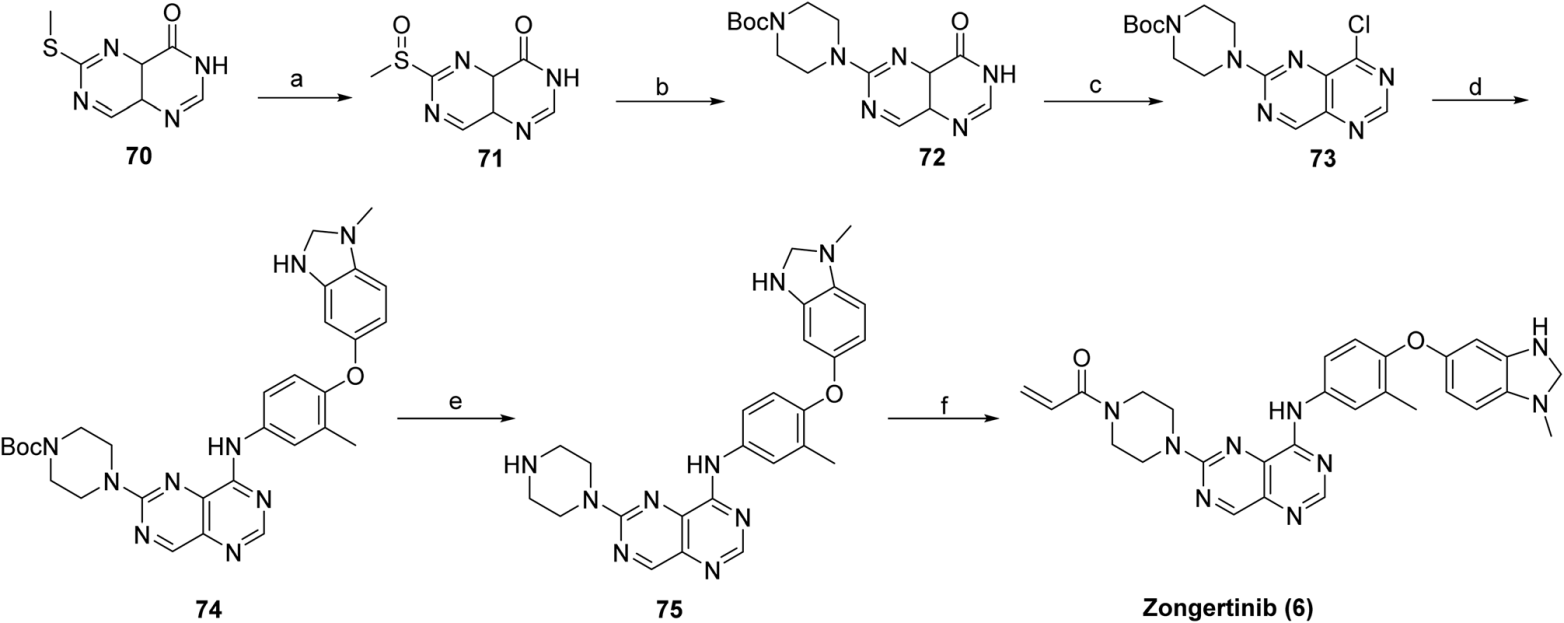
Synthesis of zongertinib (6). ^*a*^Reaction conditions: (a) *m*-CPBA, DCM; (b) 1-boc piperazine, DIPEA, DMF; (c) CoCl_2_, DCM, DMF; (d) intermediate 69, IPA; (e) HCl, 1,4-dioxane, DCM, MeOH; (f) acryloyl chloride, Et_3_N, DCM.

The synthesis of zongertinib was carried out using intermediate 69. The piperazine fragment was installed first *via* an S_N_Ar reaction after the oxidation of methyl sulfide 70 to sulfoxide 71 using *m*-CPBA to make intermediate 72, as shown in Scheme 11. Subsequent chlorination of 72 with oxalyl chloride yielded the compound 73, followed by amination with 69, which provided intermediate 74. Boc-deprotection of 74 formed the precursor intermediate 75, which was used to make compound 6 using amidation with acryloyl chloride.^55,56^

#### 2.1.7 Inluriyo™ (imlunestrant)

Inluriyo™ (imlunestrant, 7), developed by Eli Lilly and Company is an orally bioavailable, brain-penetrant selective estrogen receptor (ER) antagonist and degrader designed for the treatment of hormone receptor-positive breast cancer.^57^ Imlunestrant suppresses ER signaling by antagonizing ER-mediated transcription and inducing receptor degradation through formation of an unstable ER-ligand complex that is subsequently eliminated *via* the ubiquitin–proteasome pathway. On September 25, 2025, the U.S. FDA granted approval for imlunestrant for the treatment of adult patients with ER-positive, HER2-negative, ESR1-mutated advanced or metastatic breast cancer whose disease has progressed following at least one line of endocrine therapy. This approval was supported by efficacy data from the EMBER-3 trial (NCT04975308), a randomized, open-label, multicenter study in patients with previously treated ER-positive, HER2-negative locally advanced or metastatic breast cancer. During clinical development, imlunestrant has been evaluated as both monotherapy and in combination regimens, highlighting its potential as a next-generation oral ER degrader for endocrine-resistant disease. The drug received fast track designation from the FDA. Imlunestrant acts as a selective estrogen receptor degrader by binding to estrogen receptor alpha (ERα), inducing conformational changes that promote receptor destabilization and subsequent degradation *via* the ubiquitin–proteasome pathway, thereby suppressing estrogen-dependent transcriptional signaling.^57^ Imlunestrant carries a warning for embryo-fetal toxicity, and effective contraception is advised during treatment.

**Scheme 12:**
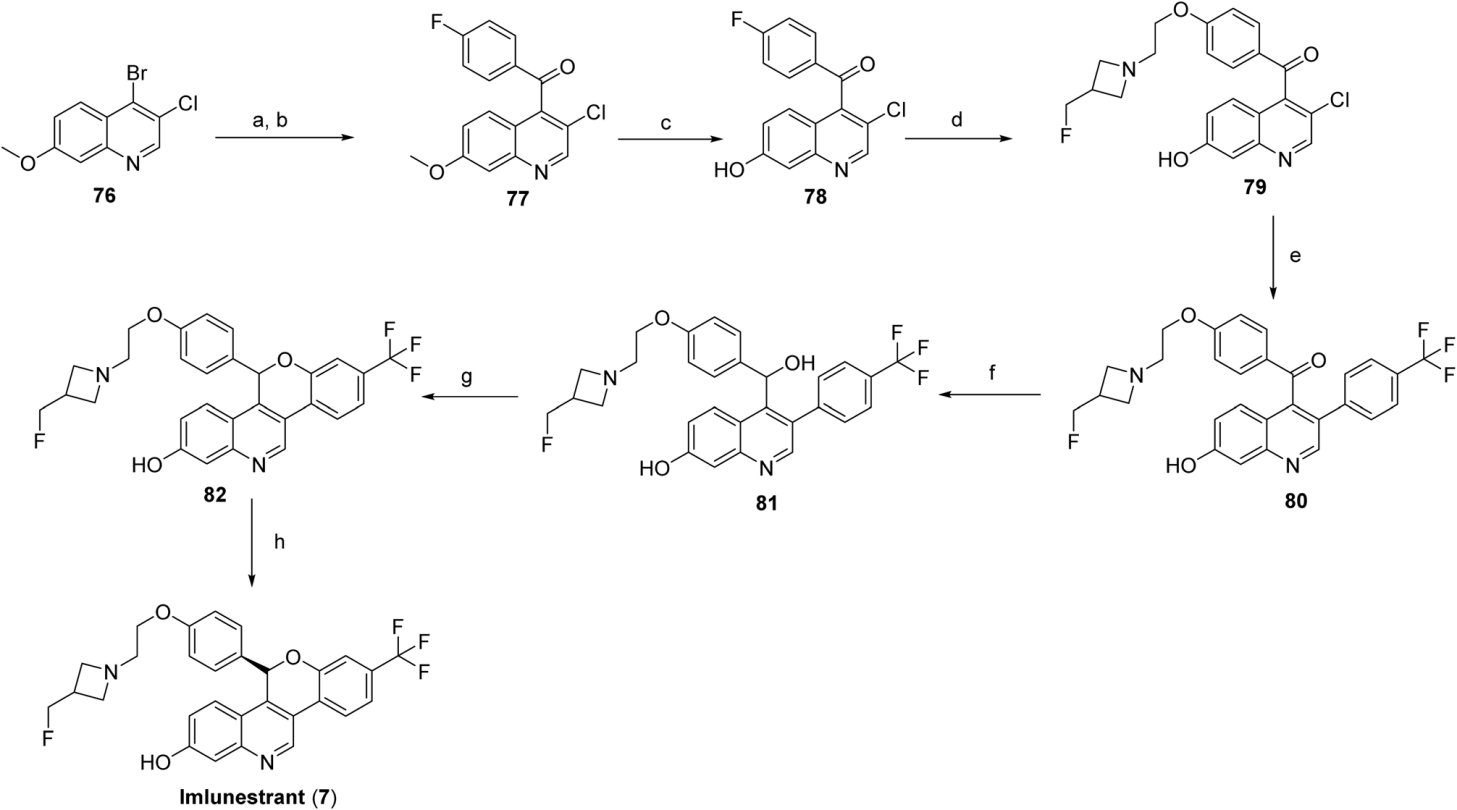
Synthesis of imlunestrant (7). ^*a*^Reaction conditions: (a) i-PrMgCl, THF; (b) 4- fluorobenzoyl chloride in THF; (c) BBr_3_, CH_2_Cl_2_; (d) 2-(3-(fluoromethyl)azetidin-1-yl)ethan-1-ol, NaH, DMF; (e) (4-(trifluoromethyl)phenyl)boronic acid, XPhos-Pd-G2 and K_2_CO_3_, 2-methyl-2-butanol/H_2_O; (f) LiBHEt_3_ in THF/1,4-dioxane; (g) NaH, THF; (h) chiral SFC.

Imlunestrant (7) is synthesized from 4-bromo-3-chloro-7-methoxyquinoline (76) *via* a metal–halogen exchange with i-PrMgCl, followed by Grignard acylation with 4-fluorobenzoyl chloride to yield (3-chloro-7-methoxy-4-quinolyl)(4-fluorophenyl)methanone (77). The 7-methoxy group is then selectively demethylated using BBr_3_ to provide the 7-hydroxyquinoline derivative (78), which undergoes ether formation with 2-[3-(fluoromethyl)azetidin-1-yl]ethanol *via* NaH-mediated alkylation, affording intermediate (79). The fluoroaryl intermediate (79) is further elaborated through a Suzuki–Miyaura cross-coupling with [4-(trifluoromethyl)phenyl]boronic acid to generate the diaryl ketone (80), which is reduced with LiBHEt_3_ to the corresponding secondary alcohol (81). Intramolecular cyclization of this alcohol under basic conditions furnishes the racemic chromeno[4,3-*c*]quinolin-2-ol core (82), which is finally resolved by chiral supercritical fluid chromatography (SFC) to yield enantiomerically pure imlunestrant (7) as shown in Scheme 12.

#### 2.1.8 Komzifti™ (ziftomenib)

Komzifti™ (ziftomenib, 8) developed by Kura Oncology, Inc. is an orally administered, small molecule menin inhibitor for the treatment of acute myeloid leukemia (AML).^58^ Ziftomenib binds to menin at the protein–protein interaction interface with KMT2A, thereby disrupting this complex and inhibiting downstream oncogenic transcriptional programs. Its antileukemic activity arises from selective disruption of the interaction between menin (MEN1) and the histone methyltransferase KMT2A (also known as MLL), a protein–protein interaction that plays a central role in maintaining the oncogenic transcriptional program in NPM1-mutant and KMT2A-rearranged AML. Inhibition of the menin-KMT2A complex downregulates oncogenic target genes and suppreses leukemic cell proliferation.^59,60^

On November 13, 2025, the U.S. FDA approved ziftomenib (Komzifti) for the treatment of adult patients with relapsed or refractory acute myeloid leukemia (AML) harboring a susceptible nucleophosmin 1 (NPM1) mutation and lacking satisfactory alternative treatment options. Approval was based on efficacy results from the KO-MEN-001 trial (NCT04067336), an open-label, multicenter, single-arm study evaluating 112 adults with relapsed or refractory NPM1-mutant AML, including tumors with type A, B, D, and other NPM1 variants associated with cytoplasmic localization of the NPM1 protein, as determined by next-generation sequencing or PCR. Ziftomenib was granted breakthrough therapy and orphan drug designations. The prescribing information includes warnings for differentiation syndrome, QTc interval prolongation, and embryo-fetal toxicity. Differentiation syndrome,^61^ which may be fatal, has been reported and requires prompt interruption of therapy, initiation of corticosteroids and appropriate clinical monitoring, followed by resumption of treatment upon symptom resolution.

**Scheme 13:**
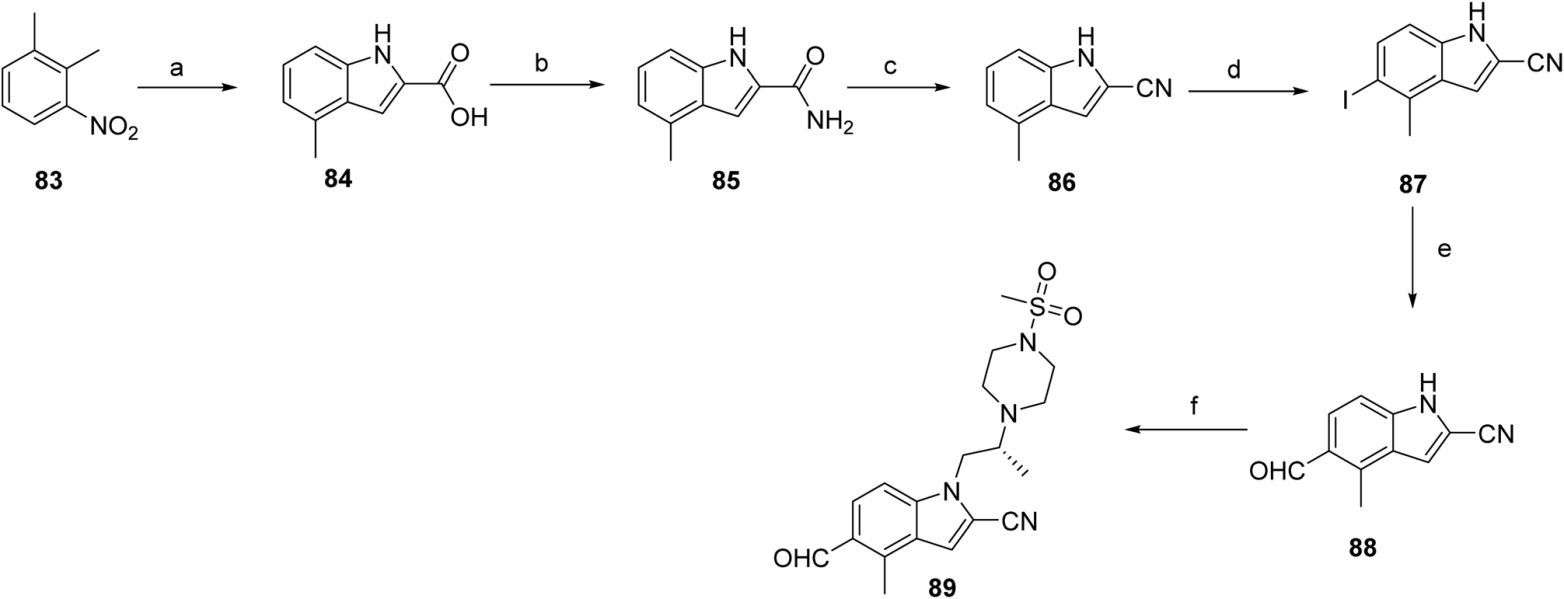
Synthesis of intermediate (89). ^*a*^Reaction conditions: (a) NaH, THF, diethyloxalate, *tert*-amyl alcohol; (b) 25% aq. ammonia, oxalyl chloride, DCM; (c) POCl_3_, toluene; (d) DCM, BF_3_·OEt_2_, NIS; (e) P(Cy)7-HBF7, Na_2_CO_3_, DMF, Pd(OAc)_2_, Et3SiH; (f) Tf_2_O, DIPEA, (*R*)-2-(4-(methylsulfonyl)piperazin-1-yl)propan-1-ol, Cs_2_CO_3._

The synthesis of ziftomenib (8) proceeds through first synthesizing the intermediates (89 & 97). Compound 84 was prepared *via* base-mediated condensation of starting material 83 with diethyl oxalate in the presence of *tert*-amyl alcohol. Activation of 84 followed by amidation furnished 85, which was subsequently converted to the intermediate 86 by reacting with phosphorus oxychloride. Electrophilic iodination of this intermediate using boron trifluoride etherate and *N*-iodosuccinimide afforded compound 87. The iodinated intermediate was then elaborated through a palladium-catalyzed carbonylative coupling to generate 88. Final functionalization involved conversion of the alcohol moiety of 88 to the corresponding triflate, followed by base-mediated coupling with (*R*)-2-(4-(methylsulfonyl)piperazin-1-yl)propan-1-ol to deliver intermediate 89 as shown in Scheme 13.

**Scheme 14:**
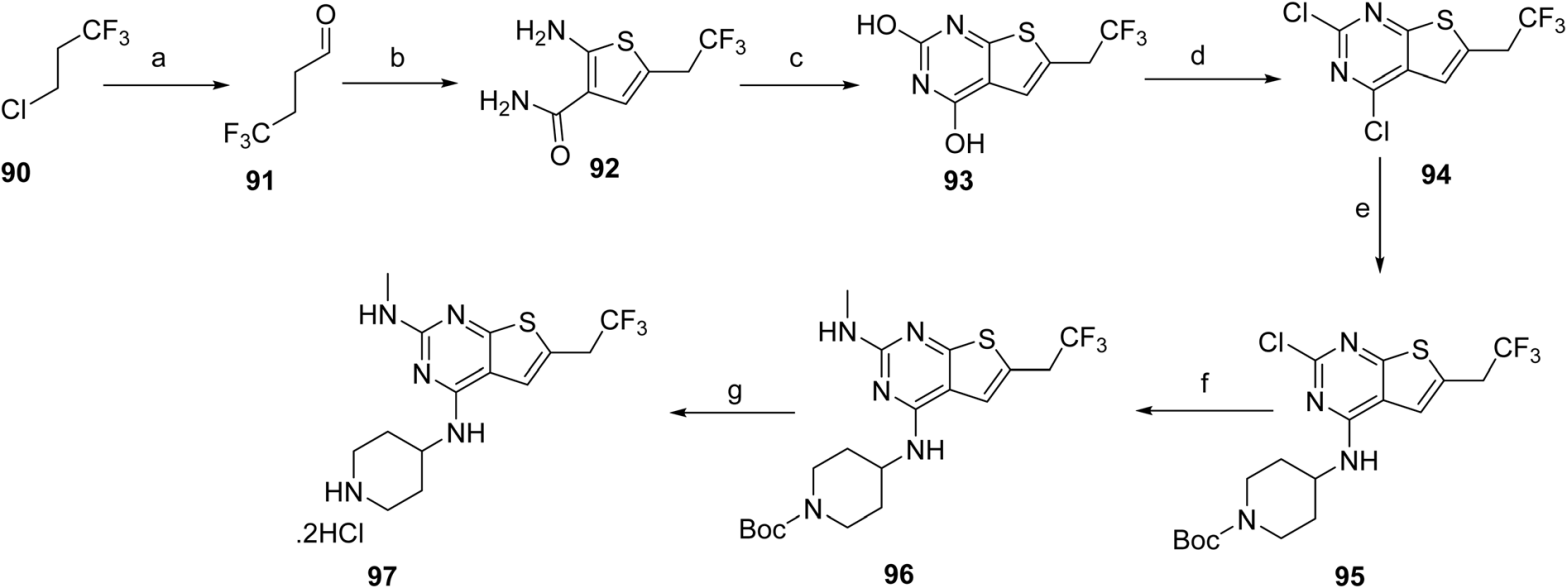
Synthesis of intermediate (97). ^*a*^Reaction conditions: (a) 1,2 dibromoethane, Mg powder, Me-THF; (b) cyanoacetamide, Me-THF, DMF, sulfur S_8_, TEA; (c) CDI, Me-THF; (d) tetraethyl ammonium chloride, POCl_3_; (e) DIPEA, *tert*-butyl 4-aminopiperidine-1-carboxylate; (f) TEA, MeNH_2_, EtOH, H_2_O; (g) 4 M HCl, MeOH.

**Scheme 15:**
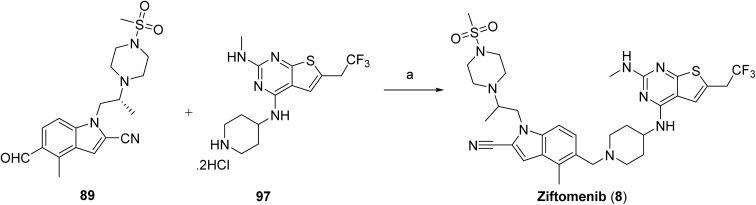
Synthesis of ziftomenib (8). ^*a*^Reaction conditions: (a) isobutyric acid, NaBH_4_, DCM, TEA.

The synthesis of intermediate 97 begins with the formation of an alkyl Grignard reagent from a halogenated trifluoropropane precursor (90), which is subsequently converted to the corresponding trifluorinated aldehyde (91). This aldehyde undergoes a multicomponent thiophene-forming reaction with 2-cyanoacetamide and elemental sulfur under basic conditions to furnish the substituted aminothiophene carboxamide (92). Cyclization of this intermediate using a carbonyl-activating reagent affords the corresponding thieno[2,3-*d*]pyrimidine-2,4-diol core (93). The heterocyclic core is then activated through chlorination to generate a dichlorothienopyrimidine intermediate (94), enabling subsequent nucleophilic aromatic substitution with a protected amine fragment to give compound 95. Further functionalization is achieved *via* amine displacement using methylamine to introduce the desired amino substituent, affording compound 96. Final deprotection under acidic conditions yields the target intermediate 97, Scheme 14. The final assembly of ziftomenib (8) was achieved through a reductive coupling strategy, Scheme 15. An activated hydride reducing system, generated from sodium borohydride and isobutyric acid, was used to mediate the reductive transformation of a preassembled mixture of intermediates 89 and 97 in the presence of a base. This reduction furnished the target ziftomenib (8) as the final product.

#### 2.1.9 Hyrnuo™ (sevabertinib)

Hyrnuo™ (sevabertinib, 9), developed by Bayer HealthCare Pharmaceuticals Inc., is an orally administered, reversible tyrosine kinase inhibitor developed for the treatment of non-small cell lung cancer (NSCLC) driven by activating HER2 (ERBB2) mutations. It is designed to inhibit a broad spectrum of HER2 alterations, including exon 20 insertions and point mutations, and functions as a dual inhibitor of HER2 and epidermal growth factor receptor (EGFR). By reversibly binding to the kinase domains of HER2 and EGFR, sevabertinib suppresses receptor phosphorylation and downstream oncogenic signaling pathways essential for tumor growth and survival, resulting in inhibition of proliferation in HER2-mutant or HER2-overexpressing cancer cells. At the protein-ligand interaction level, homology modeling based on an EGFR–ligand crystal structure suggests that sevabertinib occupies the ATP-binding pocket of HER2, forming a key hydrogen bond with the hinge residue Met801. Additional interactions with Lys753 and Asp863 of the DFG motif, along with contacts involving Thr862 and the gatekeeper region, further stabilize the inhibitor–kinase complex (PDB ID: 9QXN). Notably, no covalent interaction with Cys805 is observed, consistent with its reversible mechanism of inhibition.^62^

On November 19, 2025, the U.S. FDA granted accelerated approval to sevabertinib (Hyrnuo™) for adult patients with locally advanced or metastatic, non-squamous NSCLC harboring HER2 (ERBB2) tyrosine kinase domain-activating mutations,^54,62,63^ as detected by an FDA-approved test, who had received prior systemic therapy. The approval was based on efficacy data from the SOHO-01 trial (NCT05099172), an open-label, multicenter, single-arm, multi-cohort study evaluating sevabertinib in previously treated patients with HER2-mutant NSCLC. Sevabertinib was granted priority review and received both breakthrough therapy and orphan drug designations, highlighting its clinical significance in a molecularly defined patient population with high unmet medical need. The prescribing information includes warnings and precautions for diarrhea, hepatotoxicity, interstitial lung disease (ILD)/pneumonitis, ocular toxicity, pancreatic enzyme elevations, and embryo-fetal toxicity.

**Scheme 16:**
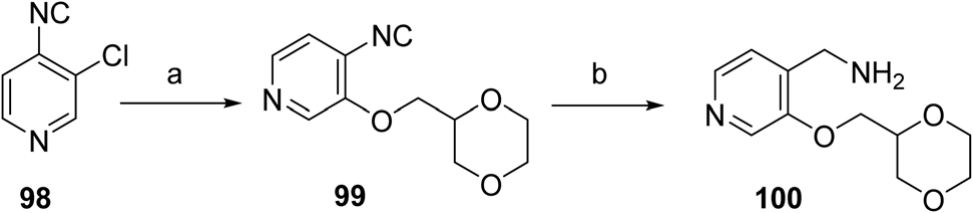
Synthesis of intermediate (100). ^*a*^Reaction conditions: (a) (1,4-dioxan-2-yl)methanol; (b) LiAlH_4_, THF.

**Scheme 17:**
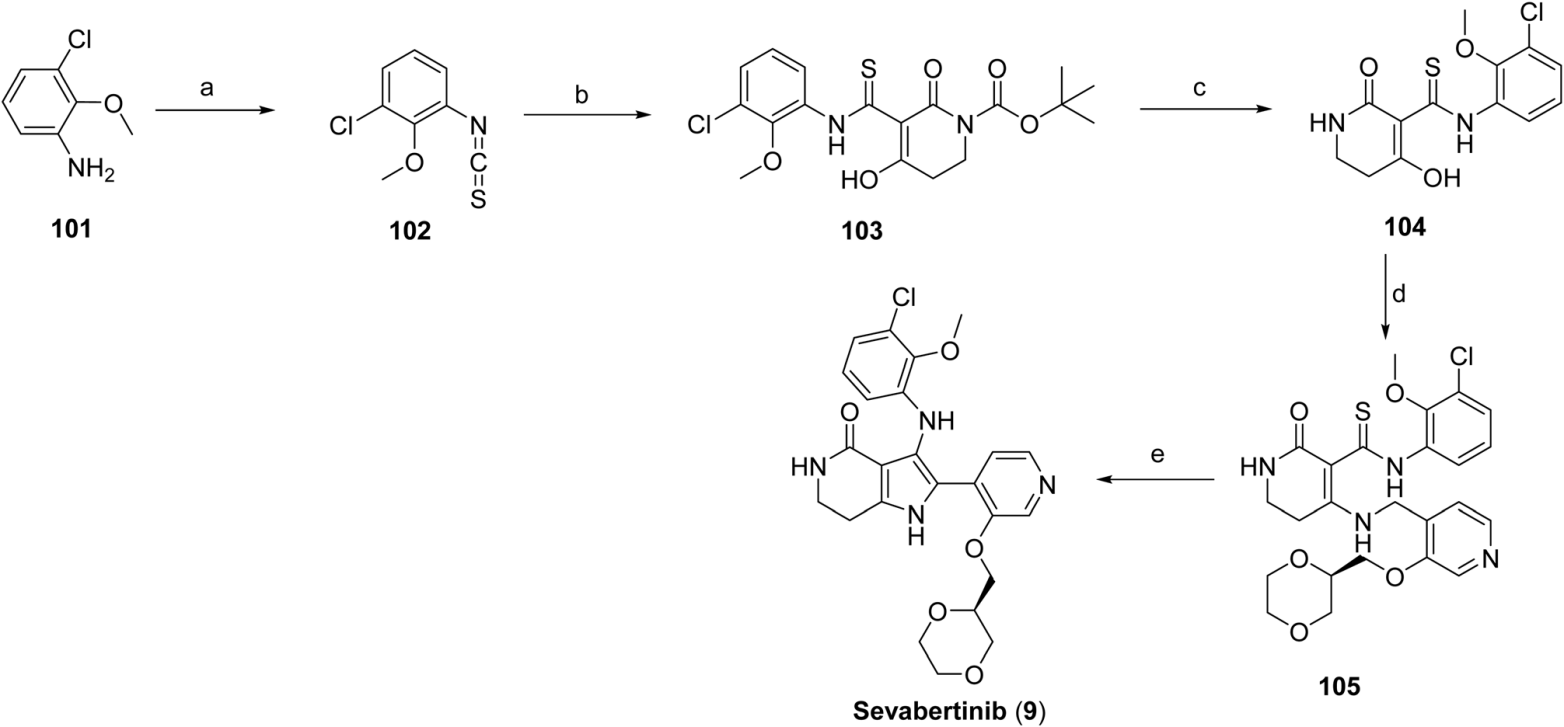
Synthesis of sevabertinib (9). ^*a*^Reaction conditions: (a) thiophosgene (CSCl_2_), DCM; (b) 1-Boc-2,4-piperidinedione, DBU, ACN; (c) DCM, TFA; (d) intermediate 100, bis(trimethylsilyl)acetamide, ACN; (e) (*S*)-(3-((1,4-dioxan-2-yl)methoxy)pyridin-4-yl)methanamine, ACN.

The synthesis of sevabertinib (9) proceeds *via* the synthesis of intermediate 100. The starting material 98 was reacted with (1,4-dioxan-2-yl)methanol to afford 99 which was subjected to catalytic hydrogenation to give the intermediate 100, Scheme 16. 3-Chloro-2-methoxyaniline (101) was converted to the corresponding isothiocyanate (102) under standard thiophosgene conditions at low temperature. The resulting intermediate was Boc-protected to afford intermediate 103, which was subsequently deprotected under acidic conditions to give intermediate 104. Intermediate 104 was then coupled with intermediate 100 at elevated temperature to furnish compound 105. Final oxidative transformation of compound 105 in methanol under acidic conditions completed the synthesis of the target compound, sevabertinib (9) (Scheme 17).

### 2.2 Non-anticancerous drugs

#### 2.2.1 Journavx™ (suzetrigine)

Journavx™ (suzetrigine, 10), developed by Vertex Pharmaceuticals, is a first-in-class, orally administered, non-opioid analgesic approved for the treatment of moderate to severe acute pain in adults. It exerts its analgesic effect by selectively blocking the voltage-gated sodium channel NaV1.8, which is predominantly expressed in peripheral nociceptive neurons, including dorsal root ganglion neurons.^64,65^ Inhibition of NaV1.8 prevents the transmission of nociceptive signals from the peripheral nervous system to the spinal cord and brain, thereby reducing pain without central opioid activity. The primary active metabolite, M6-SUZ, retains NaV1.8 inhibitory activity but is approximately 3.7-fold less potent than the parent compound. Suzetrigine is believed to exhibit state-dependent binding to the NaV1.8 channel, preferentially interacting with the inactivated conformation and stabilizing it in a non-conducting state. This interaction likely occurs within the channel pore region, thereby reducing sodium ion influx and suppressing neuronal excitability associated with pain signaling. Although high-resolution structural data are not available, this mechanism is supported by electrophysiological studies demonstrating selective inhibition of NaV1.8 currents.^64^

The U.S. FDA approved suzetrigine as the first drug in this new class of peripherally acting sodium channel-targeted analgesics. Acute pain, typically arising from tissue injury such as trauma or surgery, is commonly managed with analgesics that may include opioids, highlighting the clinical need for effective non-opioid alternatives. The efficacy of suzetrigine was demonstrated in two randomized, double-blind, placebo- and active-controlled clinical trials of acute postoperative pain following abdominoplasty and bunionectomy. In both studies, suzetrigine produced a statistically significant reduction in pain compared with placebo, with ibuprofen permitted as rescue medication for inadequate pain control. The prescribing information for Journavx includes warnings for pruritus, muscle spasms, elevated creatine phosphokinase, and rash, contraindication with strong CYP3A inhibitors, avoidance of grapefruit, and use in severe hepatic impairment.

**Scheme 18:**
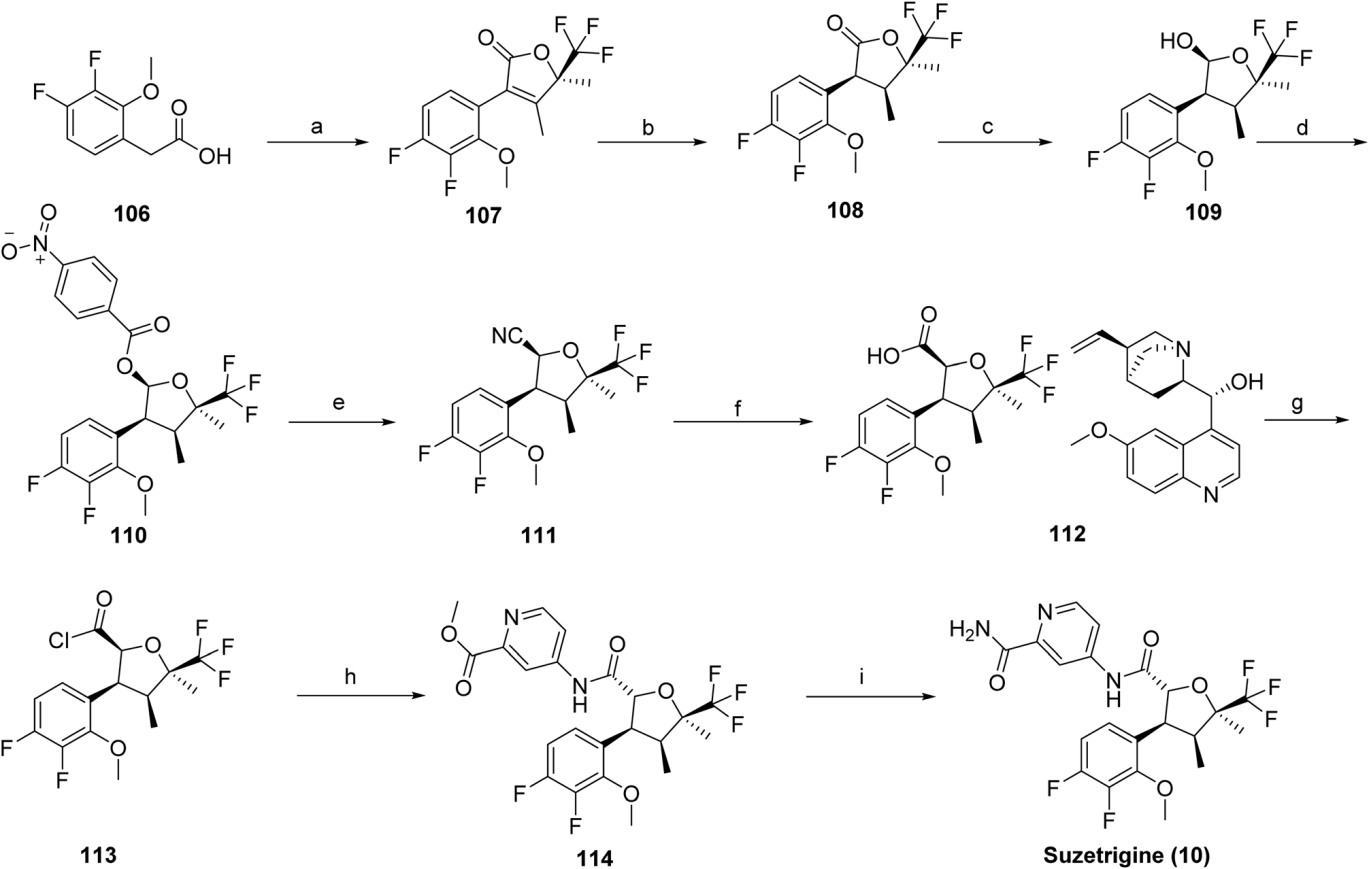
Synthesis of suzetrigine (10). ^*a*^Reaction conditions: (a) (*R*)-4,4,4-trifluoro-3-hydroxy-3-methylbutan-2-one, 1,1′-carbonyldiimidazole/ACN for 1.5 h at −2 to 0 °C, then for 5 h at 35 °C; (b) palladium–carbon; hydrogen/isopropyl alcohol for 30 h at 30–31 °C (c) diisobutylaluminium hydride/toluene for 6.5 h at −31 to −26 °C; (d) 4-nitrobenzoyl chloride, triethylamine/toluene at 0 °C; (e) trimethylsilanecarbonitrile, boron trifluoride diethyl etherate/toluene for 3 h at 20 °C; (f) quinine, isopropyl alcohol; *n*-heptane for 2 h at 65–70 °C; (g) hydrogen chloride/toluene; water at 20 °C, then for 4 h at 30 °C; (h) methyl 4-aminopicolinate, Et_3_N, DCM for 4 h at 25 °C; (i) ammonia/methanol for 24 h at 20 °C.

The chemical synthesis of suzetrigine (10)^66^ was reported by Vertex Pharmaceuticals through a series of patents and involves a multistep sequence, as outlined in Scheme 18. The synthesis begins with (*R*)-4,4,4-trifluoro-3-hydroxy-3-methylbutan-2-one, which is activated using 1,1′-carbonyldiimidazole (CDI) to generate the corresponding activated intermediate (107). Catalytic hydrogenation over Pd/C furnished the reduced product (108), which was then selectively reduced with diisobutylaluminium hydride (DIBAL-H) to afford intermediate (109). Acylation with 4-nitrobenzoyl chloride in the presence of triethylamine produced the ester derivative (110). Introduction of the nitrile functionality was achieved *via* trimethylsilyl cyanide in the presence of boron trifluoride diethyl etherate (111). Enantioenrichment was accomplished through quinine-mediated resolution (112), followed by acidic deprotection to yield the free intermediate (113). Coupling with methyl 4-aminopicolinate provided intermediate (114), which upon aminolysis with ammonia furnished the final product, suzetrigine (10).

#### 2.2.2 Blujepa™ (gepotidacin)

Blujepa™ (gepotidacin, 11), developed by GlaxoSmithKline, is a first-in-class triazaacenaphthylene antibacterial agent that inhibits bacterial DNA replication through selective dual targeting of type II topoisomerases, DNA gyrase, and topoisomerase IV.^67^ These enzymes are essential for regulating DNA topology during replication, transcription, and cell division. Gepotidacin binds to the GyrA subunit of DNA gyrase and the ParC subunit of topoisomerase IV *via* a binding mode that is mechanistically distinct from that of fluoroquinolone antibiotics.^68^ Structural studies demonstrate that gepotidacin occupies a unique pocket between the two scissile DNA bonds, stabilizing enzyme-DNA cleavage complexes. Notably, the basic nitrogen of gepotidacin interacts with the Asp83 residue of GyrA, further distinguishing its binding mode from that of quinolone antibiotics.^69^ This differentiated mechanism highlights its retained activity against pathogens resistant to established topoisomerase inhibitors.

Blujepa is approved for the treatment of uncomplicated urinary tract infections (uUTIs) in adult and pediatric female patients (≥12 years, ≥40 kg) caused by susceptible organisms, including *Escherichia coli*, *Klebsiella pneumoniae*, *Citrobacter freundii* complex, *Staphylococcus saprophyticus*, and *Enterococcus faecalis*. The safety of BLUJEPA was evaluated in 2 double-blind, active-controlled, randomized trials in female adult and pediatric patients 12 years of age and older with uUTI (Trial 1 and Trial 2). On 25 March 2025, the U.S. FDA approved Blujepa (Gepotidacin) oral tablets for patients aged 12 years and older weighing at least 45 kg, addressing an important unmet need in the management of gonorrhea. Gepotidacin is indicated for patients with limited alternative treatment options due to constrained clinical safety data. To limit the emergence of antimicrobial resistance and preserve antibacterial efficacy, BLUJEPA should be used only for infections that are confirmed or strongly suspected to be bacterial in origin.

**Scheme 19:**
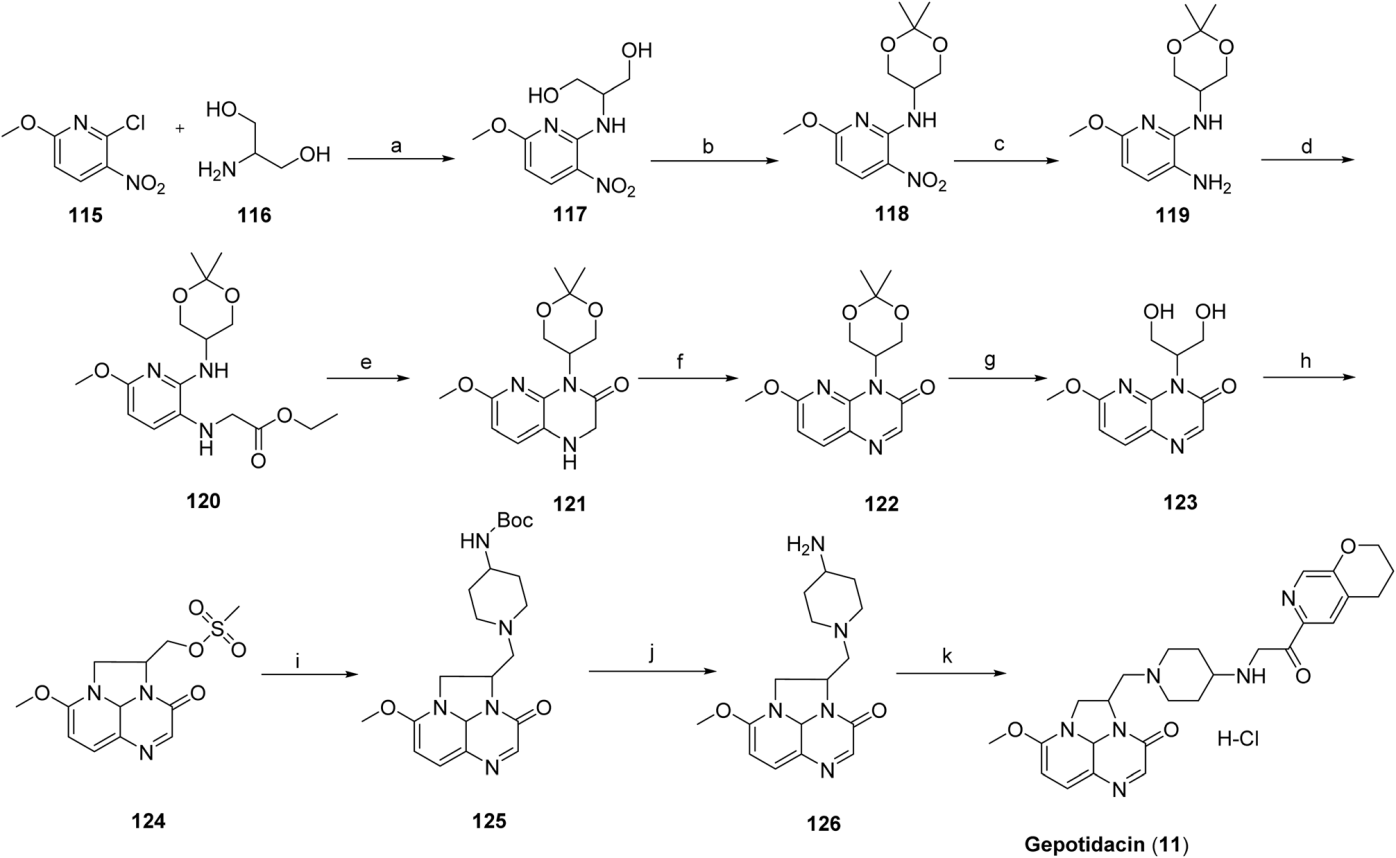
Synthesis of gepotidacin (11). ^*a*^Reaction conditions: (a) H_2_O, EtOH, (b) 2,2-dimethoxypropane, PTSA, NaHCO_3_ (c) H_2_, 10% Pd/C, 1,4-dioxane, (d) ethyl 2-bromoacetate, K_2_CO_3_, DMF (e) NaH, THF (f) MnO_2_, CH_2_Cl_2_ (g) HCl, THF (h) TEA, CHCl_3_, (i) methanesulfonic anhydride, pyridine, CH_3_CN, (j) *tert*-butyl piperidin-4-ylcarbamate, HCl, CH_2_Cl_2_, chiral chromatography (k) 3,4-dihydro-2*H*-pyrano[2,3-*c*]pyridine-6-carbaldehyde, Na(CH_3_COO)_3_BH, CHCl_3_/MeOH, HCl 1 M in Et_2_O, CH_2_Cl_2_.

The synthesis of gepotidacin^70^ has been described in two patents in 2008 (ref. 71) and 2016.^72^ The synthesis of Gepotidacin (11) proceeds with a reaction between 2-chloro-6-methoxy-3-nitropyridine (115) and 2-aminopropane-1,3-diol (116), affording the corresponding amino-substituted diol intermediate (117). Protection of the diol as its acetal using 2,2-dimethoxypropane yielded compound 118, followed by catalytic hydrogenation of the nitro group over 10% Pd/C to furnish the aniline derivative 119. Subsequent alkylation of the aniline with ethyl bromoacetate afforded intermediate 120. Base-mediated cyclization using sodium hydride generated the bicyclic intermediate 121, which was oxidized with manganese dioxide to provide compound 122. Acidic cleavage of the acetal protecting group released the free diol 123, which upon treatment with methanesulfonic anhydride, underwent intramolecular cyclization to construct the triazaacenaphthylene core (124). Introduction of the piperidine moiety was achieved *via* substitution with Boc-protected aminopiperidine to afford intermediate 125. Subsequent Boc deprotection and chiral chromatographic purification yielded the enantiomerically enriched primary amine 126. Final reductive amination with the appropriate aldehyde delivered gepotidacin as the free base, which was converted to the mono-hydrochloride salt by treatment with one equivalent of 1 M HCl in diethyl ether to give gepotidacin salt (11), Scheme 19.^73^

#### 2.2.3 Vanrafia™ (atrasentan)

Vanrafia™ atrasentan (12), developed by Novartis, is a novel, selective endothelin type A receptor antagonist (SERA) indicated to reduce proteinuria in adults with primary immunoglobulin A nephropathy (IgAN) who are at risk of rapid disease progression, typically defined by a urine protein-to-creatinine ratio (UPCR) ≥ 1.5 g g^−1^. IgAN is a rare autoimmune-mediated kidney disorder characterized by the deposition of immune complexes containing aberrant immunoglobulin A within the glomeruli, leading to inflammation, persistent proteinuria, and progressive loss of renal function.^74^ Endothelin-1 (ET-1) is implicated in the pathogenesis of IgAN by activating the ETA receptor, promoting glomerular injury and fibrosis; thus, selective ETA antagonism represents a targeted therapeutic strategy to mitigate disease progression. Atrasentan selectively antagonizes the endothelin A (ETA) receptor by competitively binding to the receptor and blocking endothelin-1 (ET-1)–mediated signaling. This inhibition suppresses downstream pathways involved in vasoconstriction, inflammation, and fibrosis, thereby reducing proteinuria and renal injury.^75^

Vanrafia was evaluated in a randomized, double-blind, placebo-controlled trial (ALIGN) with IgAN. Patients with IgAN and protein in the urine were randomly assigned to receive either Vanrafia or placebo once daily. The trial also included a subset of subjects on sodium–glucose cotransporter-2 inhibitors (SGLT2i) at baseline who were excluded from the primary efficacy analysis. The primary endpoint for accelerated approval was the percent reduction in urine protein at week 36 compared to baseline. This indication was granted accelerated approval based on reductions in proteinuria; however, it has not yet been established whether Vanrafia slows kidney function decline in patients with IgA nephropathy. Continued approval is contingent upon confirmation of clinical benefit in ongoing confirmatory trials. Vanrafia is contraindicated during pregnancy due to the risk of major birth defects, and pregnancy must be excluded prior to treatment initiation; effective contraception is required before, during, and for two weeks following therapy, and treatment should be discontinued if pregnancy occurs.

**Scheme 20:**
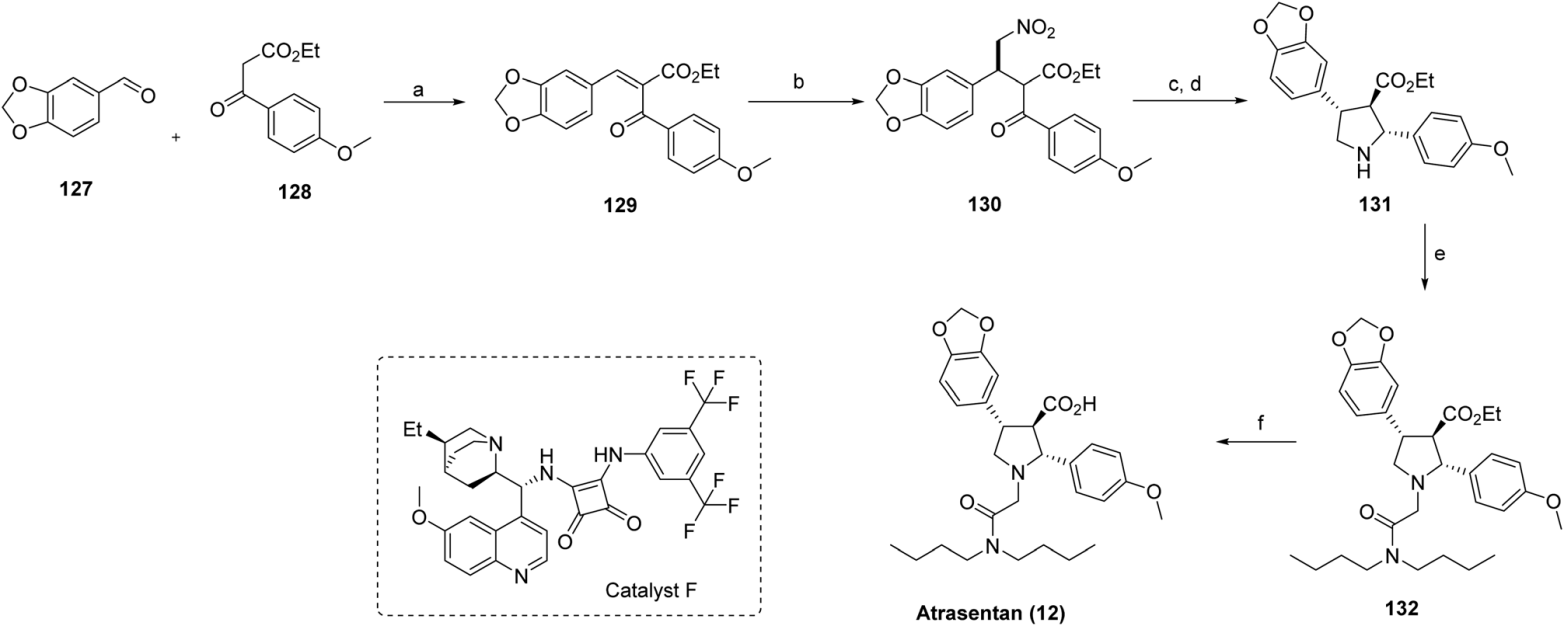
Synthesis of atrasentan (12)^a^. ^a^Reaction conditions: (a) piperidine, EtOH, acetic acid, reflux, 4 hours; (b) MeNO_2_, catalyst F, toluene, 72 h, 30 °C; (c) Raney Ni/H_2_, EtOH; (d) NaBH_3_CN, bromocresol green, THF–EtOH, 84% yield (over two steps); (e) DIPEA, *n*Bu_2_NCOCH_2_Br, DCM, 63% yield; (f) NaOH, ethanol–water, 54% yield.

Previously, the total synthesis of atrasentan (12) had been accomplished using chiral auxiliary-mediated acylation,^76^ aldol reactions,^77^ and a hetero-Diels-Alder strategy.^78^ In contrast, the current synthetic approach employs a nitromethane addition product (130) as a key intermediate. As outlined in Scheme 20, intermediate 129 is first generated *via* condensation of compounds 127 and 128. Subsequent stereoselective addition of nitromethane under optimized conditions, using catalyst F, affords the desired adduct 130 with controlled stereochemistry. Hydrogenation of 130 using Raney® Nickel produces a transient imine intermediate, which is directly reduced with sodium cyanoborohydride to furnish pyrrolidine 131 in 84% yield over two steps. The crude reaction mixture exhibited a diastereomeric ratio of 2.6 : 1; purification by column chromatography provided the major diastereoisomer of 131 in 56% isolated yield. Alkylation of pyrrolidine 131 yielded intermediate 132 in 63% yield. Final saponification of 132 in ethanol/water completed the synthesis of atrasentan (12), delivering the target compound in an overall yield of 15.7% and 67% enantiomeric excess.

#### 2.2.4 Ekterly™ (sebetralstat)

Ekterly™ (sebetralstat, 13) is an orally available plasma kallikrein inhibitor being developed by KalVista Pharmaceuticals for the on-demand treatment of acute attacks of hereditary angioedema (HAE). Sebetralstat received its first approval on 7 July 2025 in the USA,^79^ where it is indicated for the treatment of acute attacks of HAE in adult and pediatric patients aged 12 years and older. Approval was based on the results of the phase 3 KONFIDENT trial (NCT05259917; Sect. 2.3.2). Sebetralstat had received fast track and orphan drug designations from the US FDA.^79^ Hereditary angioedema (HAE) is a rare genetic disease that causes sudden, painful swelling episodes in various body locations that can be life-threatening, particularly when affecting the throat. Sebetralstat is the first and only oral on-demand treatment for HAE, providing a convenient alternative to injectable therapies that have been challenging for patients to use. This plasma kallikrein inhibitor works by reducing bradykinin, the substance that causes the characteristic swelling in HAE attacks.^80^ The prescribing information limits Ekterly use in patients on moderate or strong CYP3A4 inducers and in those with severe hepatic impairment. At the molecular level, sebetralstat binds within the active site of plasma kallikrein, occupying the S1 pocket and forming key interactions with residues such as Asp189. Structural insights from a co-crystal structure (PDB ID: 8A3Q) further support binding within the S1 and adjacent subsites, contributing to its potency and selectivity.^80^

**Scheme 21:**
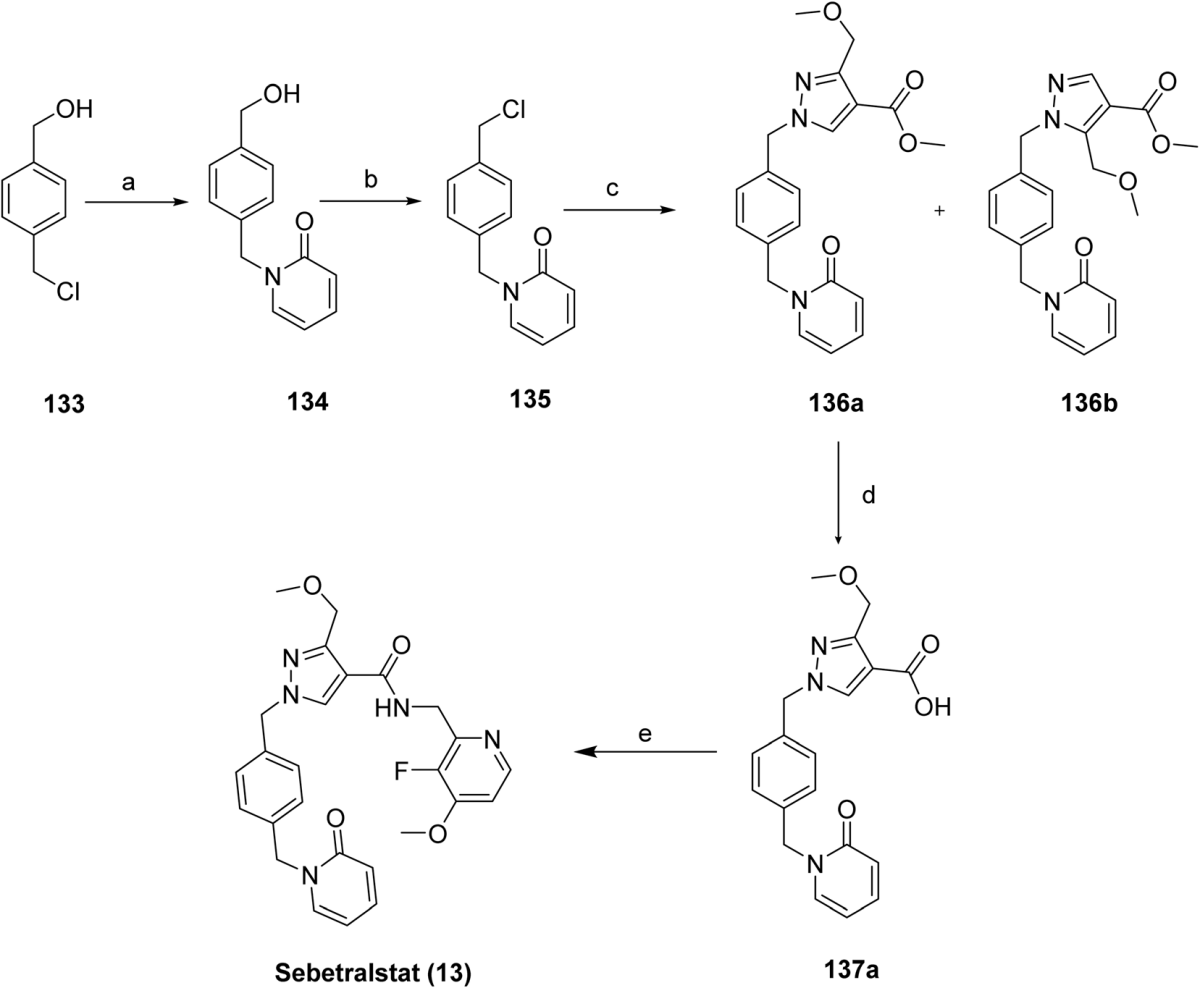
Synthesis of sebetralstat (13). ^*a*^Reaction conditions: (a) 2-hydroxypyridine, K_2_CO_3_, acetone, 50 °C, 18 h, 78%; (b) methanesulfonyl chloride, Et_3_N, dichloromethane, rt, 18h, 93%; (c) methyl 3-(methoxymethyl)-1*H*-pyrazole-4-carboxylate, K_2_CO_3_, DMF, 60 °C, 18 h, 54%; (d) 136a, NaOH, THF-MeOH-H_2_O, rt, 18 h, 34%; (e) *C*-(3-fluoro-4-methoxy-pyridin-2-yl)-methylamine, HATU, Et_3_N, dichloromethane, rt, 4 h, 64%.

Sebetralstat (13)^81^ was synthesized as shown in Scheme 21, *via* a multistep sequence involving benzyl-heterocycle functionalization and late-stage amide coupling. Nucleophilic substitution of 4-(chloromethyl)benzyl alcohol (133) with 2-hydroxypyridine afforded the benzyl-linked pyridinone 134, which was converted to the corresponding benzyl electrophile 135*via* mesylation. Alkylation of 135 with methyl 3-(methoxymethyl)-1*H*-pyrazole-4-carboxylate furnished regioisomeric pyrazole derivatives 136a/136b. 136b was a byproduct in the reaction, and the desired regioisomer 136a was isolated. Hydrolysis of the ester functionality in 136a yielded the carboxylic acid 137a, which underwent amide coupling with a substituted aminopyridine fragment to afford the target compound sebetralstat (13).

#### 2.2.5 Anzupgo™ (delgocitinib)

Anzupgo™ (delgocitinib 14) developed by Leo Pharma Inc., is the first U.S. FDA-approved topical Janus kinase (JAK) inhibitor for the treatment of moderate to severe chronic hand eczema (CHE) in adults who are unresponsive to or unsuitable for topical corticosteroids. CHE is a multifactorial inflammatory skin disorder associated with a substantial impact on quality of life and limited effective therapeutic options. Delgocitinib exerts its anti-inflammatory effects through non-selective inhibition of JAK1, JAK2, JAK3, and tyrosine kinase 2 (TYK2), resulting in suppression of key pro-inflammatory cytokines, including IFN-γ, IL-4, IL-13, IL-17A, and IL-22.^82,83^ X-ray co-crystal structure of JAK3 in complex with delgocitinib (PDB: 7C3N) reveals that the inhibitor binds within the ATP-binding pocket, forming key hydrogen-bond interactions with hinge residues Glu903 and Leu905. The cyanoacetyl group further establishes dipolar interactions with backbone residues Gly829-Lys830 and Gly834-Ser835, while the spirocyclic scaffold occupies a hydrophobic pocket adjacent to the hinge region, supporting its pan-JAK inhibitory activity.^84^ In Phase III randomized, double-blind, vehicle-controlled trials, twice-daily topical application for 16 weeks achieved treatment success rates of up to 37.7% and produced a ≥4-point reduction in Hand Eczema Symptom Diary (HESD) scores. By enabling localized drug delivery and minimizing systemic exposure, delgocitinib represents a novel, effective, and safer therapeutic option for CHE.^85^ Use of Anzupgo in combination with other JAK inhibitors or potent immunosuppressants is not recommended.

**Scheme 22:**
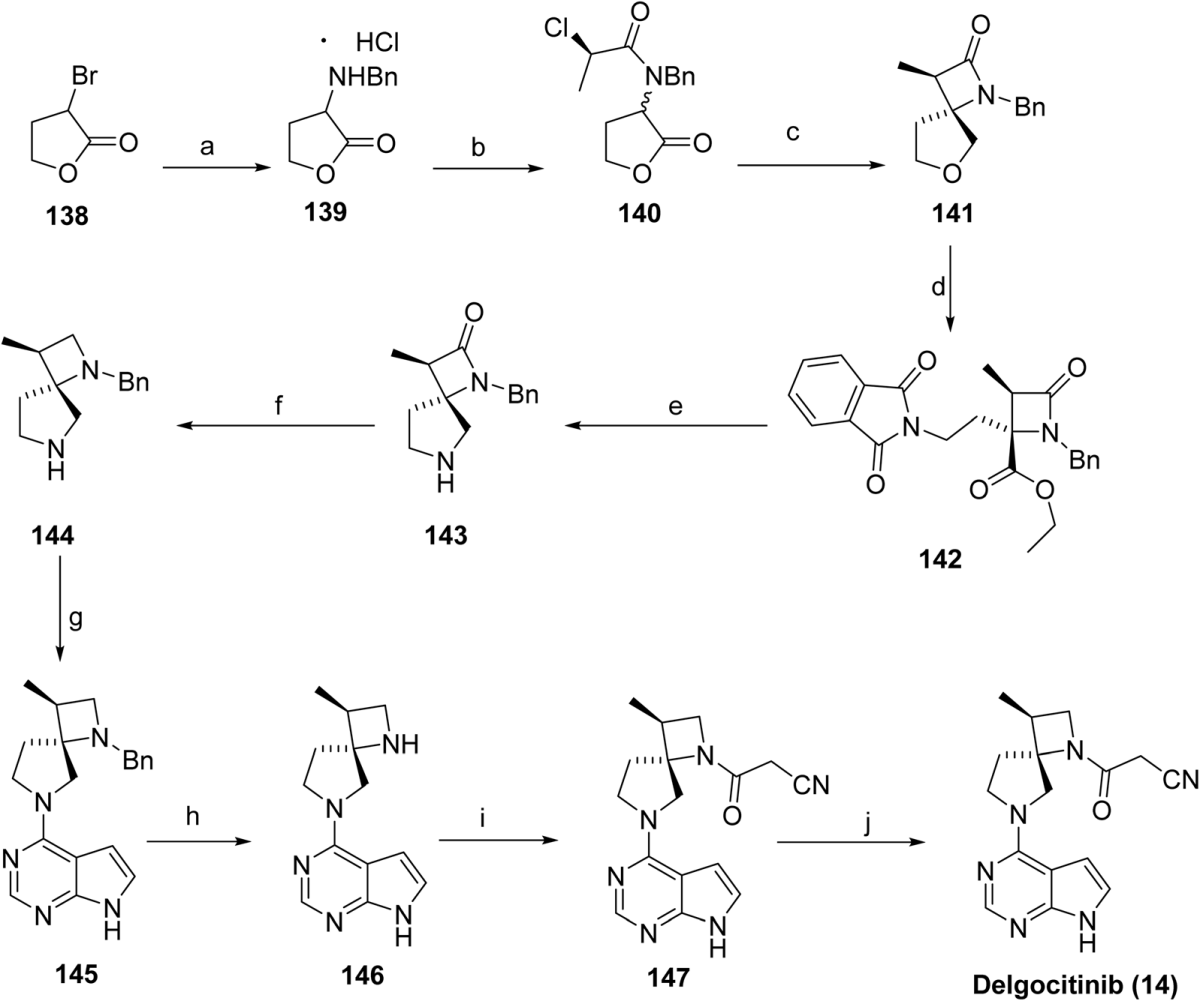
Synthesis of delgocitinib (14). ^*a*^Reaction conditions: (a) BnNH_2_, K_3_PO_4_, MeCN (b) 2,6 lutidine, EtOH, 6 °C, 3 h; (c) LHMDS, PhMe, −9 °C, 1 h; (d) potassium phthalimide, DMSO, 104 °C, 11 h, ethyliodide, 46 °C, 2 h; (e) HN(CH_2_CH_2_NH_2_)_2_, 2-BuOH, 85 °C, 4 h; (f) TMSCl, LiAlH_4_, PhMe-THF; (g) 4-chloro-7*H*-pyrrolo[2,3-*d*]pyrimidine, K_3_PO_4_, *t*-BuOH-H_2_O, 75 °C; (h) H_2_, 5% Pd/C. AcOH; (i) 3-(3,5-dimethyl-1*H*-pyrazol-1-yl)-3-oxopropanenitrile, MeCN, 75 °C; (j) crystallization, *n*-BuOH.

The stereocontrolled synthesis of delgocitinib (14)^86^ was accomplished *via* a modular sequence (Scheme 22). Initial SN_2_ coupling of bromolactone 138 with benzylamine furnished the α-amino lactone 139 as the HCl salt. Acylation of 139 with an enantiomerically pure acid chloride afforded lactone 140. Treatment of 140 with LHMDS generated the corresponding enolate, which underwent intramolecular SN_2_ displacement of the chloride, forming the spirocyclic lactone 141 with high stereocontrol (dr 98 : 2, 96% ee). The lactone ring of 141 was subsequently opened by nucleophilic attack of potassium phthalimide at the γ-carbon, and the resulting carboxylic acid was converted to the ethyl ester. Subsequent treatment with diethylenetriamine released the phthalimide, enabling cyclization to the spirolactam 142*via* the ethyl ester intermediate (80% yield over four steps, >99% de). Reduction of the carbonyls in 143 with LiAlH_4_/AlCl_3_ in THF provided diamine 144, isolated as the succinic acid salt in 86% yield. Finally, SNAr reaction of 144 with chloropyrrolopyrimidine resulted in 145, followed by hydrogenolytic removal of the benzyl group, furnished amine 146 (92% yield over 2 steps). Acylation of 146 with cyanoacetyl pyrazole afforded 147 and recrystallization from *n*-butanol containing 3 wt% BHT provided delgocitinib (15) in 86% yield, >99% ee, and >99% de.

#### 2.2.6 Sephience™ (sepiapterin)

Sephience™ (sepiapterin, 15) developed by PTC Therapeutics, Inc., is a small-molecule activator of phenylalanine hydroxylase (PAH) used to lower phenylalanine levels in patients with phenylketonuria. It serves as a natural precursor to the enzymatic cofactor tetrahydrobiopterin (BH4), thereby enhancing PAH activity by stabilizing misfolded enzyme conformations and increasing intracellular BH_4_ availability, which collectively promotes more efficient phenylalanine metabolism. Sepiapterin does not directly bind to PAH as a classical active-site inhibitor or ligand but functions through intracellular conversion to tetrahydrobiopterin (BH_4_), which acts as an essential cofactor for PAH. The increased BH_4_ availability stabilizes the active conformation of PAH and enhances catalytic turnover of phenylalanine, thereby restoring enzymatic function.^87^ It was approved by the U.S. FDA in July 2025 for the treatment of adults and children with phenylketonuria. Phenylketonuria (PKU) is an inherited metabolic disorder caused by reduced PAH activity, the enzyme that converts phenylalanine to tyrosine, leading to elevated blood phenylalanine levels and, if untreated, progressive neurocognitive impairment.^88^ The FDA approved sephience based on evidence from Trial 1 (NCT05099640) of patients with PKU and supportive clinical evidence from Trial 2. Sephience is to be used in conjunction with a phenylalanine (Phe)– restricted diet.

**Scheme 23:**
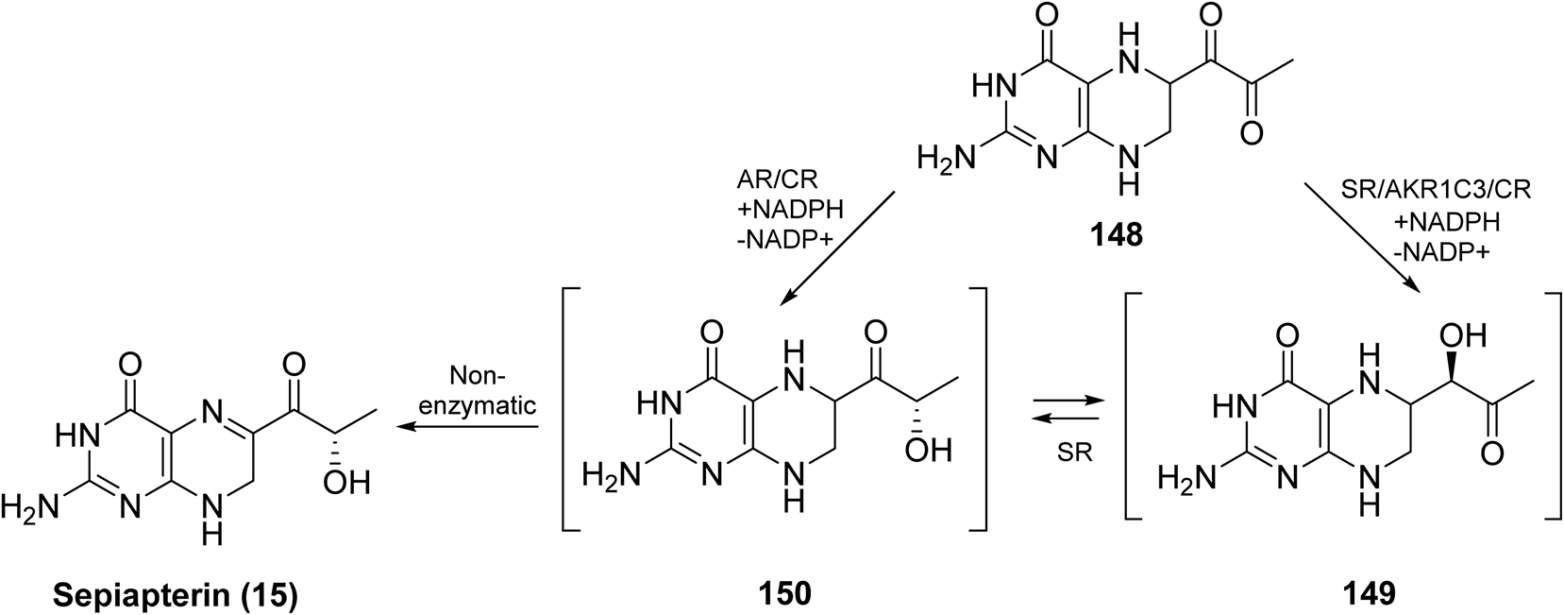
Synthesis of sepiapterin (15) *via* enzymatic and non-enzymatic pathways.

6-Pyruvoyl tetrahydropterin (148) can be converted into two intermediates: 2′-oxo-tetrahydropterin (149) and 1′-oxo-tetrahydropterin, also called lactoyl tetrahydropterin (150), with formation of 150 being more favorable (Scheme 23). 3α-Hydroxysteroid dehydrogenase type 2 efficiently converts 148 into 149, while aldose reductase (AR) can transform 148 into either 150 or 149, which can then be further processed to tetrahydrobiopterin (BH_4_). In the absence of sepiapterin reductase (SR), BH_4_ can be synthesized *via* two alternative routes: one through 149, involving the sequential action of 3α-hydroxysteroid dehydrogenase type 2 and AR and consuming 2 NADPH equivalents; and another through 150, proceeding through 148 → 150 →15, with the participation of AR, CR, and dihydrofolate reductase, including a nonenzymatic reduction to Sepiapterin, consuming 3 NADPH equivalents.

#### 2.2.7 Vizz™ (aceclidine)

Vizz™ (aceclidine, 16), developed by LENZ Therapeutics, Inc., has recently been approved as a noninvasive, nonsurgical ophthalmic treatment for presbyopia in adults, representing the first aceclidine-based therapy approved for this indication. Presbyopia is a prevalent age-related visual disorder characterized by a progressive loss of accommodative ability, resulting in impaired near vision.^89^ Aceclidine functions primarily as a pupil-selective miotic, inducing contraction of the iris sphincter while exerting minimal stimulation of the ciliary muscle. This selective miotic action produces a pinhole effect, generating a pupil diameter of less than 2 mm, thereby increasing depth of focus and enhancing near visual acuity without causing a myopic shift. Aceclidine acts as an orthosteric agonist of muscarinic acetylcholine receptors, preferentially activating M3 receptors on the iris sphincter. This interaction triggers G protein-mediated signaling, leading to calcium mobilization and smooth muscle contraction, resulting in pupil constriction.^90^ Owing to its preferential pupil-selective pharmacological profile, aceclidine has been proposed to offer advantages over conventional miotics; however, definitive head-to-head comparative clinical studies remain limited.^91^ On 31st July 2025, the FDA approved Vizz based on results from three randomized, double-masked, controlled Phase 3 studies. CLARITY 1 and CLARITY 2 were designed to evaluate the safety and efficacy of Vizz.

**Scheme 24:**
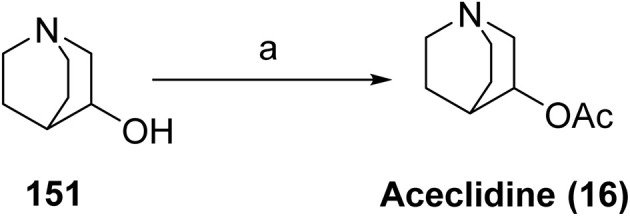
Synthesis of aceclidine (16). ^*a*^Reaction conditions: (a) acetic anhydride.

Aceclidine is synthesized as shown in Scheme 24. Subsequent *O*-acetylation of this secondary alcohol 3-quinuclidinol (151) using acetic anhydride in the presence of an appropriate base affords 3-acetoxyquinuclidine (16).

#### 2.2.8 Brinsupri™ (brensocatib)

Brinsupri™ (brensocatib, 17) developed by Insmed, is an oral small-molecule inhibitor of dipeptidyl peptidase 1 (DPP1) used to reduce neutrophilic inflammation associated with non-cystic fibrosis bronchiectasis (NCFB). It is the first drug specifically approved to treat this inflammatory lung disease. NCFB is a chronic lung disease, a progressive immune-mediated disease that causes excessive mucus production, persistent cough, and widening of the airways, impairing lung function. Brensocatib inhibits neutrophil serine proteases, addressing a key driver for this inflammation. Brensocatib acts as a selective inhibitor of dipeptidyl peptidase 1 (DPP1) by binding within its catalytic site, thereby preventing activation of neutrophil serine proteases. This inhibition reduces downstream inflammatory tissue damage associated with excessive neutrophil activity.^92^ It reduces exacerbation and slows lung function decline, offering a targeted approach to managing NCFB. On August 12, 2025, Brensocatib received U.S. FDA approval for the treatment of NCFB in adult and pediatric patients. Regular monitoring is required for dermatologic, gingival, and periodontal adverse reactions during the therapy. Avoid the use of live attenuated vaccines during treatment, as their safety and effectiveness are unknown.

**Scheme 25:**
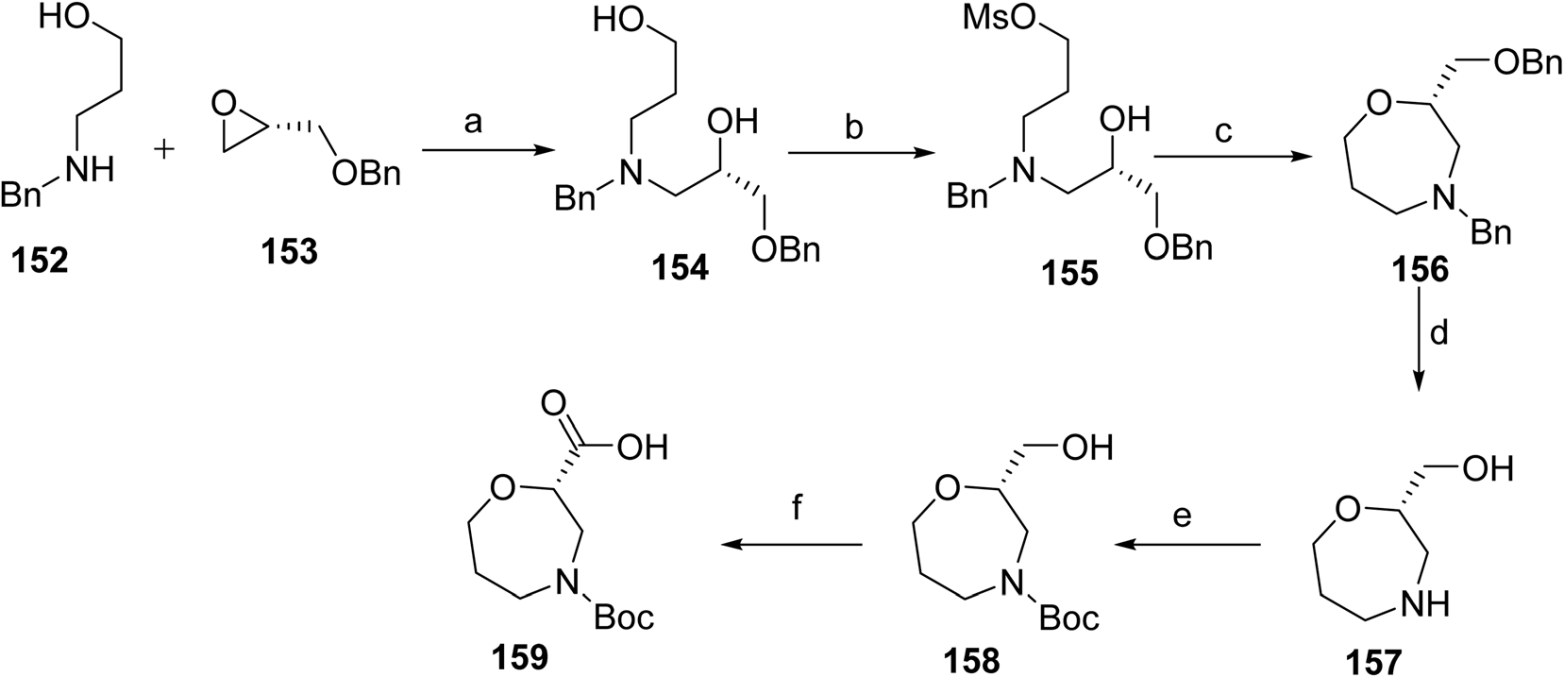
Synthesis of intermediate (159). ^*a*^Reaction conditions: (a) i-PrOH, 50 °C, 24 h; (b) MsCl, DIPEA, CH_2_Cl_2_, −6 °C, 1 h; (c) NaH, THF, SGC; (d) H_2_, EtOH, r. t, 18 h; (e) Boc_2_O, MeOH; (f) Bu_4_NHSO_4_, TEMPO, NaBr, KHSO_4_.

The synthesis of brensocatib (17) proceeds *via* the synthesis of an intermediate 159, Scheme 25. The benzylamino propanol (155) and the oxirane derivative (156) were stirred in i-PrOH to afford the corresponding intermediate (154). The reaction mixture is treated with MsCl to form the mesylate derivative (155). Subsequent treatment with NaH promoted the cyclization 156. The resulting intermediate was subjected to catalytic hydrogenation to afford 157, which was then protected using Boc_2_O to give 158. Finally, oxidation was carried out using Bu_4_NHSO_4_ to furnish the target intermediate (159) after standard aqueous workup and purification.

**Scheme 26:**
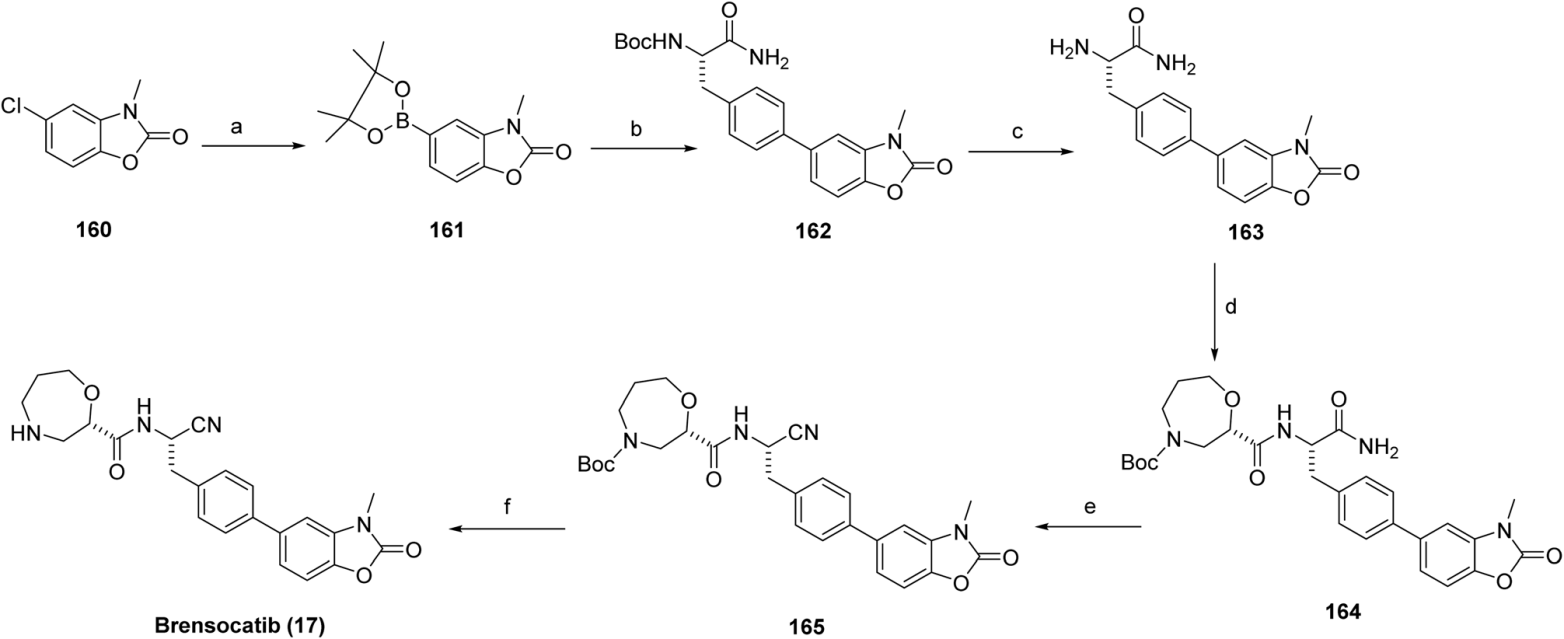
Synthesis of brensocatib (17). ^*a*^Reaction conditions: (a) B_2_pin_2_, KOAc, Pd(OAc)_2_, X-Phos, 1,4-dioxane, 75–80 °C, 2 h; (b) *tert*-butyl (*S*)-(1-amino-3-(4-iodophenyl)-1-oxopropan-2-yl)carbamate, PdCl_2_(dppf), K_2_CO_3_, 1,4-dioxane-H_2_O, 75 °C, 3 h; (c) HCl, CH_2_Cl_2_-1,4-dioxane, 15 °C, 2 h, 68%; (d) intermediate 159 (*S*)-4-(*tert*-butoxycarbonyl)-1,4-oxazepane-2-carboxylic acid, T3P, DIPEA, DMF, 25 °C, 1.5 h; (e) T3P, DIPEA, DMF, 50 °C, 4 h, 98%; (f) HCO_2_H–H_2_O (10 : 1), room temperature, 3 h.

The synthesis of brensocatib (17) commenced with a palladium-catalyzed borylation of the 160 to afford the corresponding boronate intermediate (161), which was subsequently subjected to Suzuki–Miyaura cross-coupling with a protected chiral aryl amide (*tert*-butyl(*S*)-(1-amino-3-(4-iodophenyl)-1-oxopropan-2-yl)carbamate) under standard palladium catalysis to generate the biaryl intermediate (162). Acidic deprotection furnished the corresponding amine (163), which was coupled with the oxazepane carboxylic acid intermediate (159) using a peptide-coupling reagent to form the desired amide linkage (164). The amide is efficiently converted to a nitrile *via* T3P mediated dehydration to give 165. Final acidic treatment provided the target compound brensocatib (17), Scheme 26.

#### 2.2.9 Wayrilz™ (rilzabrutinib)

Wayrilz™ (rilzabrutinib, 18), is an orally bioavailable, reversible covalent inhibitor of Bruton's tyrosine kinase (BTK)^93^ developed by Principia Biopharma (a Sanofi company). The compound demonstrates potent and durable BTK inhibition through reversible covalent engagement of the active site cysteine, translating into robust *in vivo* efficacy in rodent models of inflammatory arthritis and clinical activity in canine pemphigus foliaceus. BTK inhibition by rilzabrutinib modulates pathogenic Fc receptor and B-cell receptor-mediated signaling pathways implicated in autoantibody-driven diseases.^93,94^ Rilzabrutinib inhibits BTK *via* reversible covalent engagement of a cysteine residue proximal to the ATP-binding pocket, resulting in prolonged target occupancy and suppression of B-cell receptor-mediated signaling. Clinical efficacy and safety were established in the Phase 3 PEGASUS trial in adults with pemphigus and the LUNA3 trial in immune thrombocytopenia (ITP).^95^ On August 29, 2025, the U.S. FDA approved rilzabrutinib for the treatment of adults with persistent or chronic ITP who have shown an insufficient response to prior therapies, including immunoglobulins, anti-D therapy, or corticosteroids. A key limitation of Wayrilz is its potential to increase the risk of serious infections, including bacterial, viral, and fungal.

**Scheme 27:**
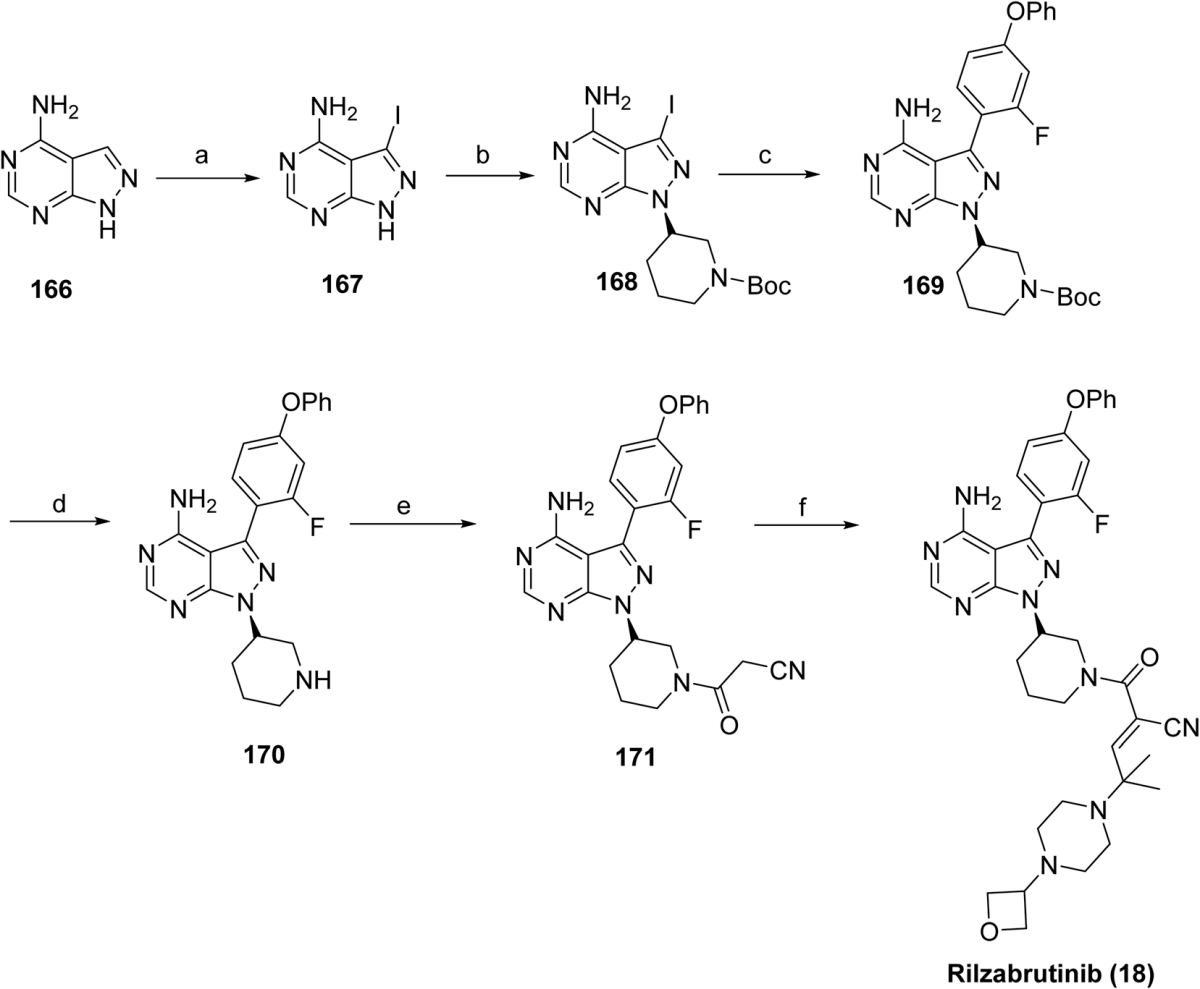
Synthesis of rilzabrutinib (18). ^*a*^Reaction conditions: (a) *N*-iodosuccinimide, DMF, 80 °C; (b) *tert*-butyl(*S*)-3-hydroxypiperidine-1-carboxylate, PPh_3_, DIAD, THF; (c) (2-fluoro-4-phenoxyphenyl)boronic acid, K_2_CO_3_, 80 °C, 1,4-dioxane/water; (d) TFA, CH_2_Cl_2_; (e) cyanoacetic acid, HOBt, EDCI, CH_2_Cl_2_; (f) 2-methyl-2-(4-(oxetan-3-yl)piperazin-1-yl)propanal, pyrrolidine, TMSCl, CH_2_Cl_2_.

Sanofi reported a synthetic route to rilzabrutinib in 2022 (Scheme 27).^93^ The synthesis commenced with 4-aminopyrazolo[3,4-*d*]pyrimidine 166 as the starting material, which was sequentially transformed into cyanoacetamide derivative 171*via* five steps: iodination (167), Mitsunobu reaction (168), Suzuki–Miyaura coupling (169), Boc deprotection (170), and amidation with cyanoacetic acid, affording a total yield of 3% of 171. The final step involved a Knoevenagel condensation between 171 and 2-methyl-2-(4-(oxetan-3-yl)piperazin-1-yl)propanal, yielding a 9 : 1 *E*/*Z* mixture, from which the *Z* isomer was removed by chiralpak IC HPLC purification, yielding rilzabrutinib (18). Overall, the reported route is limited by low cumulative yields (0.5% over eight steps), high cost, and labor-intensive procedures, emphasizing the need for a more efficient and economical synthetic strategy. A recent study describes an alternative approach to rilzabrutinib using an *E*-configured cyanoacrylic acid intermediate.^96^

#### 2.2.10 Palsonify™ (paltusotine)

Palsonify™ (paltusotine, 19) developed by Crinetics Pharmaceuticals, Inc., is an orally bioavailable somatostatin receptor agonist. It was approved by the U.S. FDA on 25th September 2025 to treat adults with acromegaly who have had an inadequate response to surgery and/or for whom surgery is not an option. Acromegaly is a hormonal disorder characterized by the overproduction of growth hormone (GH), which leads to elevated levels of insulin-like growth factor I (IGF-I).^97^ The FDA approval of palsonify was supported by results from the Phase 3 pivotal trials, PATHFNDR-1 and PATHFNDR-2, which assessed its safety and efficacy in both previously treated and treatment-naïve adults with acromegaly. In these studies, palsonify consistently showed a rapid onset of action, dependable biochemical control, and durable therapeutic effects. At the molecular level, paltusotine acts as a selective agonist of somatostatin receptor subtype 2 (SSTR2), binding to the orthosteric site of this G protein-coupled receptor and activating Gi-mediated signaling, which suppresses adenylate cyclase activity and reduces growth hormone secretion.^98^ The limitations of palsonify include the risk of gallstones, blood sugar fluctuations, slow heart rate, thyroid abnormalities, fat malabsorption, and altered vitamin B_12_ levels.

**Scheme 28:**
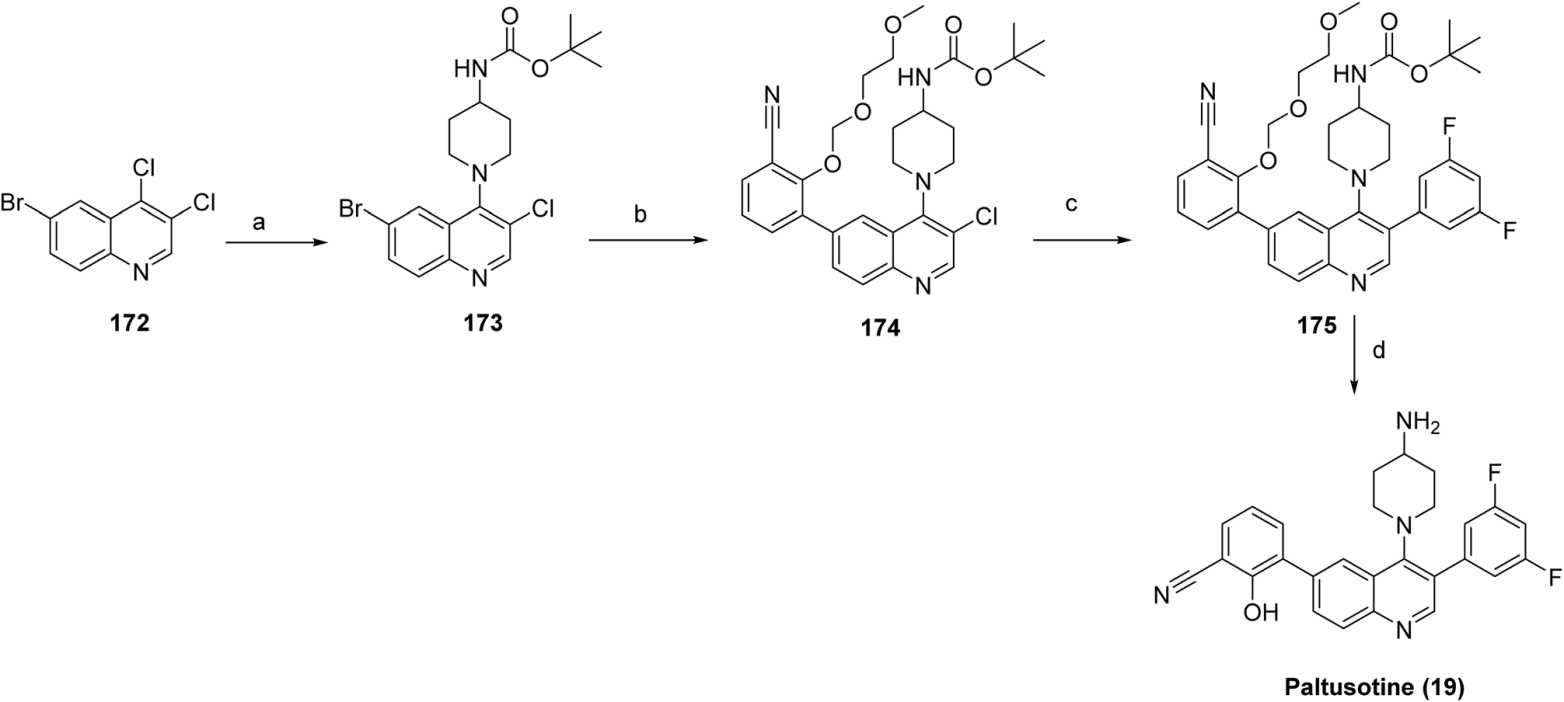
Synthesis of paltusotine (19). ^*a*^Reaction conditions: (a) *tert*-butyl piperidin-4-ylcarbamate, DIPEA, 60 °C, 63% (b) 2-(2-methoxy-ethoxymethoxy)-3-(4,4,5,5-tetramethyl-[1,3,2]dioxaborolan-2-yl)-benzonitrile, PdCl_2_dppf, KOAc, 80 °C, 1 h; (c) 3, 5-difluorophenyl boronic acid, Pd (amphos)Cl_2_, K_2_CO_3_, 95 °C; (d) TFA, ACN/water.

The target compound paltusotine (19)^99^ was synthesized through a modular, stepwise cross-coupling strategy (Scheme 28). Nucleophilic aromatic substitution of 6-bromo-3,4-dichloroquinoline (172) with *tert*-butyl piperidin-4-ylcarbamate provided the corresponding quinoline–piperidine intermediate (173). Suzuki–Miyaura cross-coupling of 173 with a protected 2-(2-methoxy-ethoxymethoxy)-3-boronate benzonitrile generated the aryl-substituted intermediate 174, which underwent a second Suzuki coupling with 3,5-difluorophenylboronic acid to furnish the fully substituted quinoline derivative 175. Finally, deprotection of the *tert*-butyl carbamate under acidic conditions followed by isolation as the hydrochloride salt afforded the final target compound paltusotine (19) in high purity.

#### 2.2.11 Rhapsido™ (remibrutinib)

Rhapsido™ (remibrutinib, 20), developed by Novartis, is an oral, small-molecule BTK inhibitor with high potency, selectivity, and covalent target engagement. BTK is an intracellular kinase expressed in mast cells, basophils, B cells, macrophages, and platelets, where it mediates signaling through the B-cell receptor (BCR) and Fc receptors.^100^ Aberrant BTK signaling contributes to chronic spontaneous urticaria (CSU) by promoting mast cell and basophil degranulation and the release of histamine and other pro-inflammatory mediators. Consistent with this mechanism, remibrutinib effectively inhibits IgE and IgG-mediated activation of mast cells and basophils, attenuating mast cell-driven inflammatory responses in CSU. Remibrutinib covalently binds to Cys481 within the ATP-binding site of BTK and forms key hinge interactions with Met477, stabilizing the inactive kinase conformation and leading to sustained inhibition of BTK signaling.^101^ On September 30, 2025, the U.S. FDA approved remibrutinib as the first oral, targeted therapy for CSU. Beyond CSU, remibrutinib has also been explored in other immune-mediated disorders,^102^ including autoimmune encephalomyelitis and Sjögren's syndrome.^101^ Its limitation is that it is not indicated for other forms of urticaria.

**Scheme 29:**
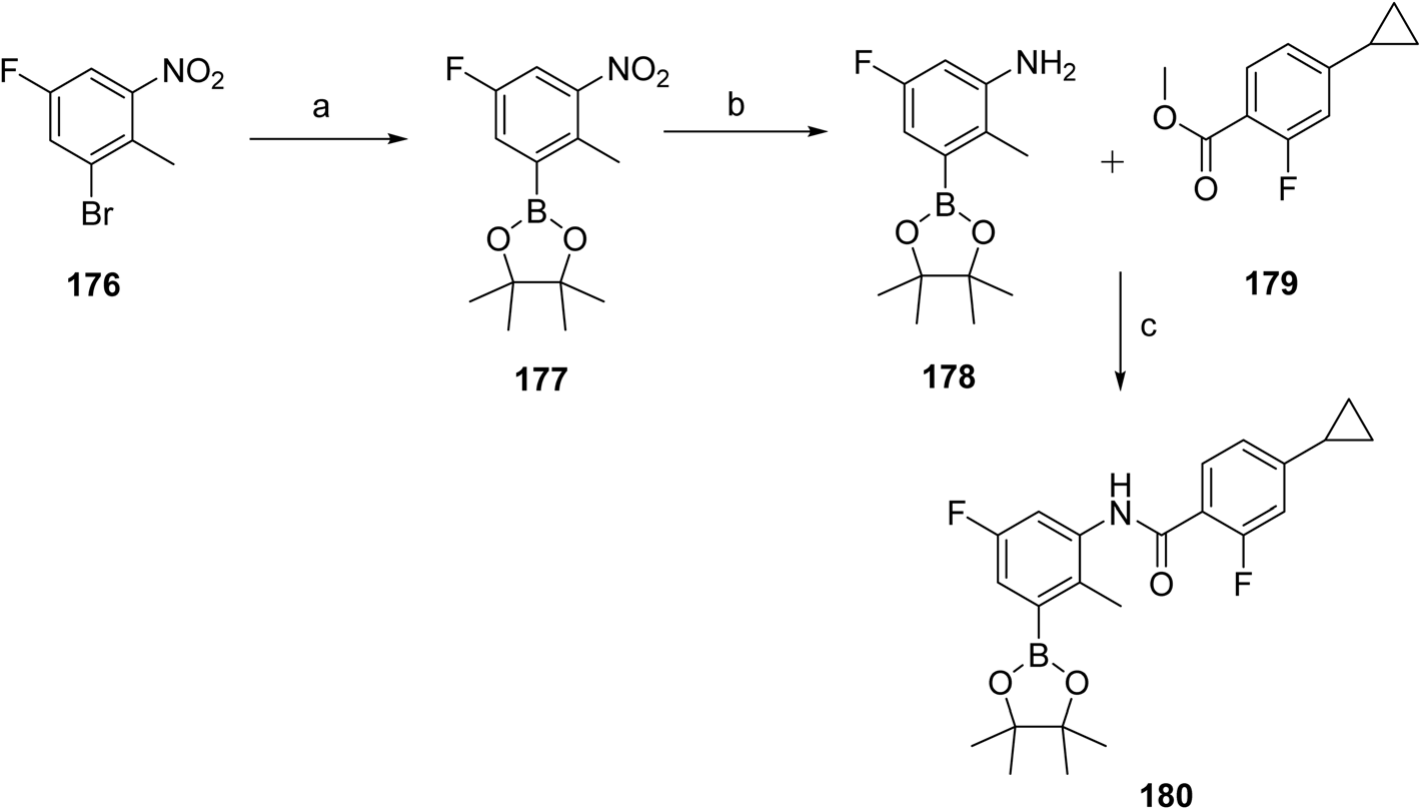
Synthesis of intermediate (180). ^*a*^Reaction conditions: (a) BISPIN, Pd(dppf)Cl_2_·DCM, KOAc, 1,4-dioxane, 100 °C, 3.5 h, 92%; (b) H_2_, Pd/C, MeOH, RT, 7 h, 93%; (c) NaHMDS (1 M in THF), THF, RT, 4 h, 76%.

**Scheme 30:**
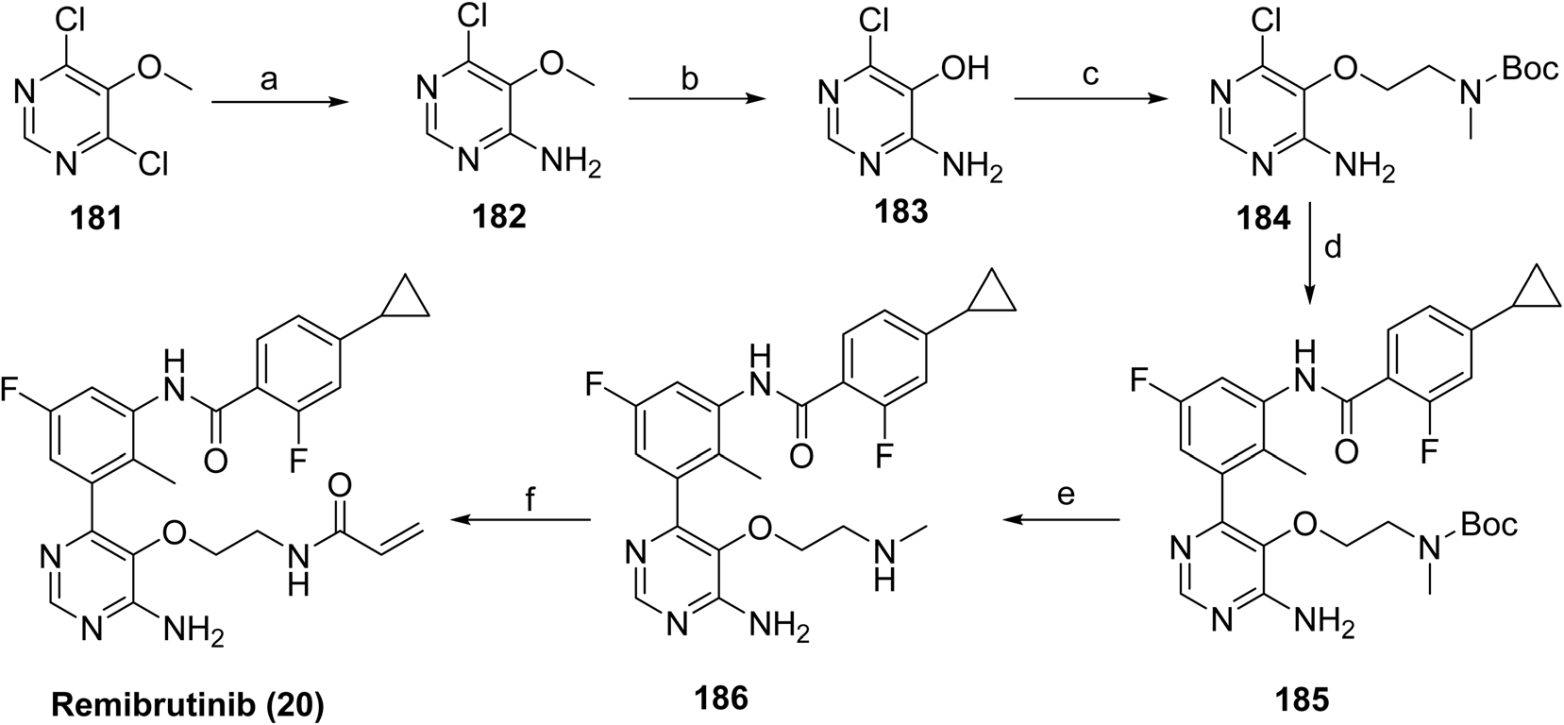
Synthesis of remibrutinib (20). ^*a*^Reaction conditions: (a) NH_4_OH, 2-propanol, 70 °C, 48 h, 94%; (b) BBr_3_, DCM, 40 °C, 3 h, 59%; (c) *N*-Boc-*N*-methyl-2-hydroxyethylamine, DIAD, Smopex-301, THF, 60 °C, 2 h, 53%; (d) 180, PdCl_2_(PPh_3_)_2_, aq Na_2_CO_3_, DME, water, microwave, 110 °C, 25 min, 74%; (e) TFA, DCM, RT, 12 h; (f) acrylic acid, DIPEA, T3P (50% in DMF), DMF, RT, 2 h, 45% over 2 steps.

The synthesis of remibrutinib (20)^103^ is outlined in Schemes 29 and 30 where Scheme 29 depicts the synthesis of the upper portion of the molecule, intermediate 180. Borylation of 1-bromo-5-fluoro-2-methyl-3-nitrobenzene (176) with bis(pinacolato)diboron in the presence of Pd(dppf)Cl_2_·DCM and potassium acetate gave boronic ester 177, which upon catalytic hydrogenation provided aniline 178. Boronic ester 181 was then accessed *via* a NaHMDS mediated amide formation of aniline 178 and cyclopropyl intermediate 179. The synthesis of 20 was completed as outlined in Scheme 30. The lower portion of the molecule was assembled starting from 2,4-dichloro-3-methoxypyrimidine (181). Treatment with ammonia in a pressure reactor yielded in aminopyrimidine 182, which was subjected to boron tribromide treatment to give aminopyrimidinol 183. The linker moiety was introduced *via* a Mitsunobu reaction with *N*-Boc-*N*-methyl-2-hydroxyethylamine in the presence of DIAD and Smopex-301, a convenient polymer-supported version of triphenylphosphine, to obtain 184. Boc-protected intermediate 185 was assembled *via* Suzuki coupling of 184 and 180. Finally, Boc deprotected 186 was followed by amide coupling with acrylic acid in the presence of propylphosphonic anhydride (T3P) provided remibrutinib (20).

#### 2.2.12 Jascayd™ (nerandomilast)

Jascayd™ (nerandomilast, 21) is developed by Boehringer Ingelheim, is an oral, selective phosphodiesterase 4 (PDE4) inhibitor with preferential activity against the PDE4B isoenzyme, administered as its *R*-enantiomer for the treatment of idiopathic pulmonary fibrosis (IPF) in adults. By inhibiting PDE4B, nerandomilast increases intracellular cAMP levels and exerts antifibrotic and immunomodulatory effects by downregulating of profibrotic growth factors and inflammatory cytokines, including MAPK, TGF-β, and TNF-α, which are typically overexpressed in IPF.^104^ Nerandomilast inhibits PDE4 by binding to its catalytic site, increasing intracellular cAMP levels and suppressing downstream inflammatory and fibrotic signaling. The U.S. FDA approved nerandomilast on October 7, 2025, for IPF and on December 19, 2025, for progressive pulmonary fibrosis (PPF) in adults. PPF is a chronic condition characterized by irreversible lung fibrosis, leading to worsening respiratory function, and represents a shared targetable pathway across interstitial lung diseases. Jascayd received approval for IFP based on positive outcomes from the FIBRONEER-IPF (NCT05321069) and Trial 2 (NCT04419506) studies. Its approval for progressive pulmonary fibrosis (PPF) was supported by the FIBRONEER-ILD trial (NCT05321082), which demonstrated a meaningful benefit in slowing lung function decline compared with placebo.^105^ Jascayd received a breakthrough therapy designation for PPF.

**Scheme 31:**
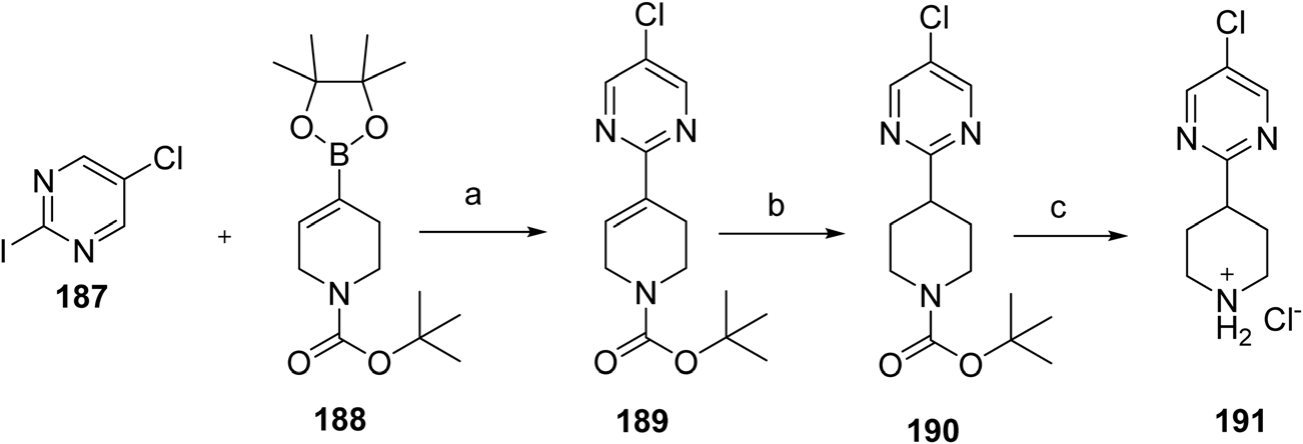
Synthesis of intermediate (191). ^*a*^Reaction conditions: (a) (i) acetonitrile (2.00 mL g^−1^), 2-propanol (2.00 mL g^−1^), 20% aq. K_3_PO_4_ (1.50 equiv.), water (1.25 mL g^−1^), PdCl_2_ (AmPhos)_2_ (0.05 mol%), 78 °C, (ii) *N*-acetylcysteine (0.04 equiv.), 70 °C, (iii) water (1.40 mL g^−1^), 91%; (b) methanol (6.50 mL g^−1^), Sponge Nickel A-5000 (10 mol%), H_2_ (4.00 bar), 50 °C; (c) (i) methanol (3.70 mL g^−1^), HCl (36% aqueous, 1.50 equiv.), 65 °C, (ii) MTBE (6.1 mL g^−1^), 74.3%.

**Scheme 32:**
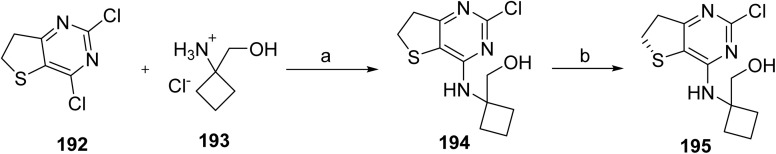
Synthesis of intermediate (195). ^*a*^Reaction conditions: (a) (i) CH_3_CN (2.00 mL g^−1^), Et_3_N (4.00 equiv.), 75 °C, (ii) methanol (2.00 mL g^−1^), (iii) recrystallization from ethanol (6.00 mL g^−1^), 76.0%; (b) CH_2_Cl_2_ (1.00 mL g^−1^), (S)-(–)-BINOL (2.0 mol%), (i-PrO)_4_Ti (1.0 mol%), water (0.20 equiv.), *t*-BuOOH (70% aqueous, 1.10 equiv.), 20–25 °C, 87.4%.

**Scheme 33:**
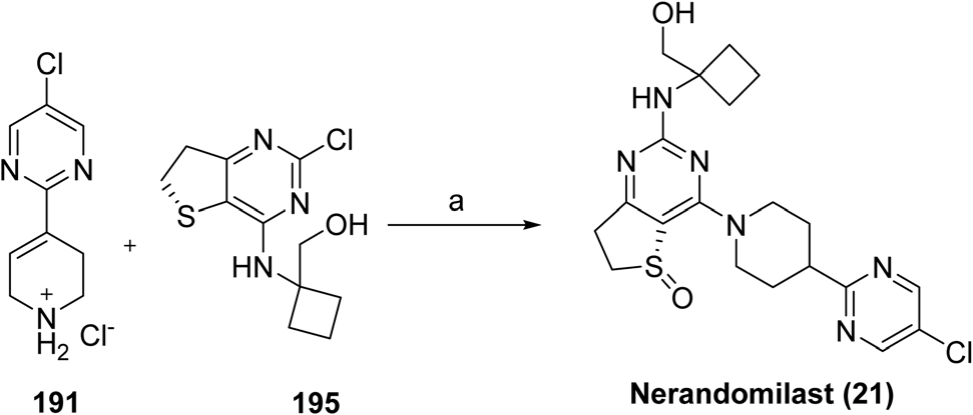
Synthesis of nerandomilast (21). ^*a*^Reaction conditions: (a) DIPEA, THF, H_2_O, 65 °C for 14 h.

The synthesis for nerandomilast (21) proceeds *via* coupling of intermediates synthesized from Schemes 31 and 32 shown in Scheme 33.^106^ Intermediate 191 is synthesized *via* a key Suzuki coupling using commercially available iodo-pyrimidine 187 and boronic ester 188 with 0.05 mol% PdCl_2_(AmPhos)_2_ as the catalyst, upon completion, *N*-acetylcysteine was added (to reduce residual Pd in the product), and the product was crystallized by adding water as the antisolvent to give 189. Subsequently, 189 was reduced by a Sponge (Raney) Nickel catalyzed (10 mol%) hydrogenation (4.00 bar H_2_ pressure), which gave 190. Subsequent cleavage of the BOC protecting group in 190 was accomplished, followed by crystallization from MTBE to its hydrochloride salt (191).

The synthesis of chiral sulfoxide intermediate 195 was carried out as shown in Scheme 32. The synthesis of 195 was accomplished by carrying out an S_N_Ar displacement of the chlorine at the C4 of 192 with commercially available 1-aminocyclobanemethanol hydrochloride (193). This transformation was not completely regioselective, and approximately 10% of the product resulted from C2-chlorine the displacement was also observed. However, this by-product was completely removed during the isolation of 194. Intermediate 194 was then recrystallized from ethanol to remove traces of triethylamine or other impurities that could affect the subsequent asymmetric oxidation to chiral sulfoxide 195, which was carried out using a modification of the procedure reported by Uemura and co-workers^107^ for the general asymmetric oxidation of sulfides. Accordingly, oxidation of 194 in the presence of a catalyst made from (*S*)-BINOL (2 mol%) and (i-PrO)_4_Ti (1 mol%), and with *tert*-BuOOH as the stoichiometric oxidant, sulfoxide 195 in good yield (87.4%) and enantioselectivity (>99.0 %ee).

With robust and scalable processes for the synthesis of intermediates 191 and 195 at hand, the final chemical step in the synthesis, which was carried out as shown in Scheme 33 by the S_N_Ar reaction between 191 and 195 in the presence of Hünig's base and a solvent system consisting of a mixture of THF and water. Under these conditions, nerandomilast (21) was obtained with good yield (>88%) and purity (>99%) as a product.

#### 2.2.13 Lynkuet™ (elinzanetant)

Lynkuet™ (elinzanetant, 22), developed by Bayer Health Care Pharmaceuticals, Inc. is an orally administered, non-hormonal, selective dual antagonist of neurokinin 1 (NK-1) and neurokinin 3 (NK-3) receptors.^108^ On October 24, 2025, it was approved by the U.S. FDA for the treatment of moderate to severe vasomotor symptoms (VMS), including hot flashes and night sweats, making it the third FDA-approved nonhormonal option for menopause-related VMS. These symptoms are linked to the activity of peptide neurotransmitters and neuropeptides, such as neurokinin A, substance P, neurokinin B (NKB), and kisspeptin, which regulate neuroendocrine and reproductive functions. During menopause, declining estrogen levels lead to hyperactivation of kisspeptin/NKB/dynorphin (KNDy) neurons, disrupting thermoregulation and triggering VMS.^109–111^ Elinzanetant functions as a dual antagonist of NK1 and NK3 receptors by binding to their orthosteric sites, thereby blocking tachykinin-mediated signaling involved in thermoregulation. Elinzanetant was also shown to lower estradiol and luteal-phase progesterone levels in serum in a dose-dependent manner.

**Scheme 34:**
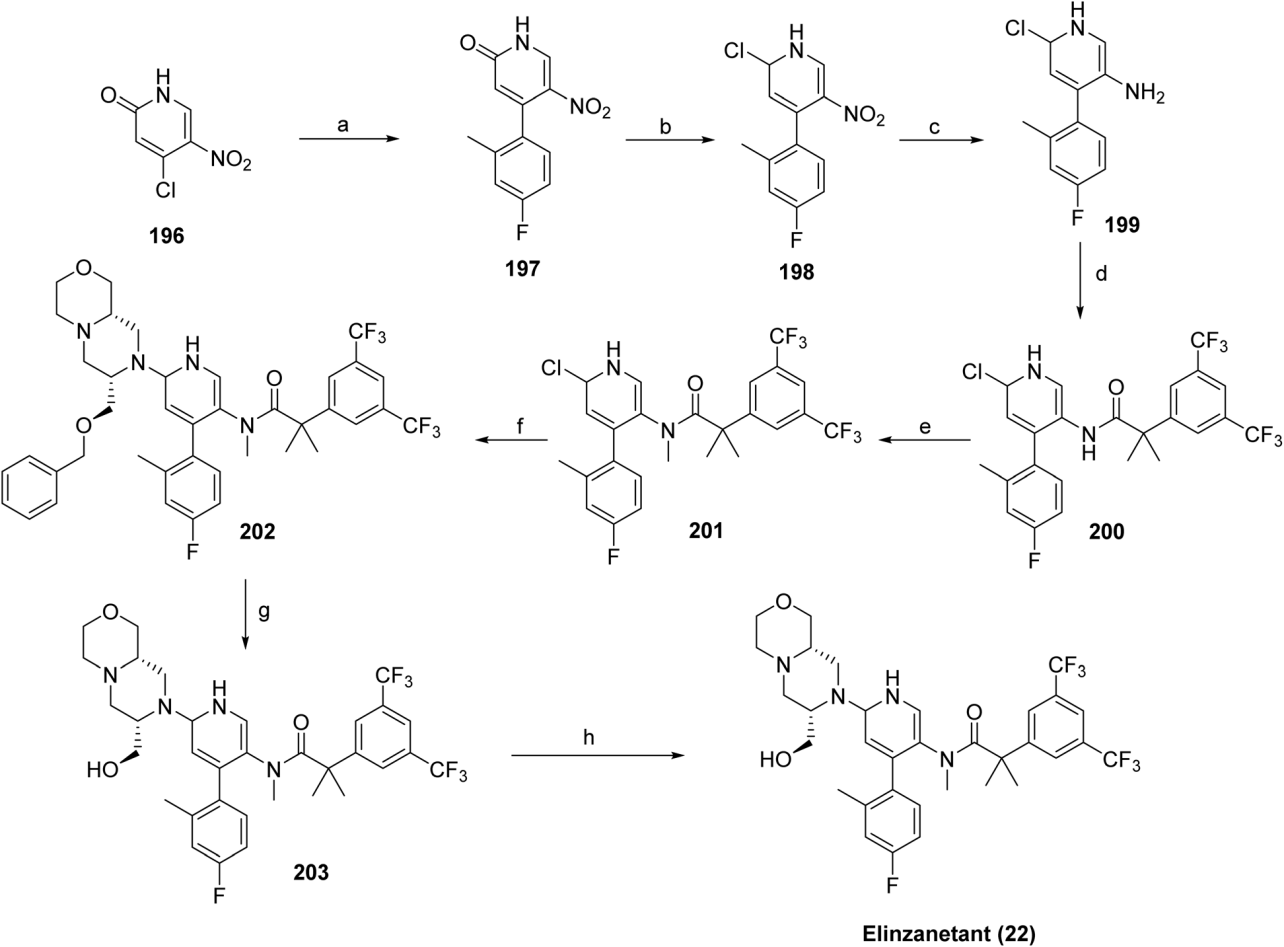
Synthesis of elinzanetant (22). ^*a*^Reaction conditions: (a) (4-fluoro-2-methylphenyl)boronic acid, (Pd(PPh_3_)_4_), K_2_CO_3_, 1,4-dioxane; (b) POCl_3_, DMF; (c) Pt/C, EtOAc (d) 2-(3,5-bis(trifluoromethyl)phenyl)-2-methylpropanoic acid, DCM, oxalyl chloride; (e) CsCO_3_, CH_3_Cl, DMF, (f) Bis(tri-*tert*-butylphosphine)palladium, NaOtBu; (g) H_2_, Pd/C, isopropanol (IPA), HCl, IPA, (h) NaOH, MTBE, IPA.

The synthesis of elinzanetant (22) shown in Scheme 34, proceeds with a palladium-catalyzed cross-coupling of the 196 and arylboronic acid under basic conditions in 1,4-dioxane to afford the coupled intermediate (197). Subsequent formylation using POCl_3_/DMF generated the corresponding chlorine derivative (198) where the nitro group was then reduced over Pt/C in ethyl acetate to yield the amine derivative (199). Amide intermediate (200) was generated by acid and amine (199) coupling. Later methylation was achieved under basic conditions in DMF to introduce the desired methyl group, followed by a palladium-catalyzed coupling of oxazine derivative to afford intermediate 202. Final hydrogenation over Pd/C in isopropanol under acidic conditions caused the selective hydrogenolytic cleavage of the benzyl ether protecting group (203), and the crude product was purified by basic workup with NaOH in MTBE/IPA to afford the target compound elinzanetant (22).

#### 2.2.14 Kygevvi™ (doxecitine and doxribtimine)

The U.S. FDA approved Kygevvi (doxecitine (23a) and doxribtimine (23b)) powder to treat thymidine kinase 2 deficiency (TK2d) in adults and pediatric patients who start to show symptoms when they are 12 years old or younger. Kygevvi received Breakthrough Therapy Designation for this indication. TK2d is a rare, inherited genetic disorder that affects the body's ability to produce and repair mitochondrial DNA (mtDNA). Conditions that cause low levels of mtDNA, including TK2d, can be called mitochondrial depletion syndromes. Doxecitine and doxribtimine act as nucleoside substrates that replenish mitochondrial deoxynucleotide pools, thereby enabling mitochondrial DNA replication and repair in the absence of functional thymidine kinase 2. Symptoms of TK2d can include muscle weakness and respiratory (breathing) failure. While the exact frequency of TKd2 is not known, it is considered very rare. FDA approval was supported by safety and efficacy data from one Phase 2 clinical study Trial 1, (NCT03845712), two retrospective chart review studies (Study 1 NCT03701568 and Study 2 NCT05017818, and an expanded access use program.

**Scheme 35:**
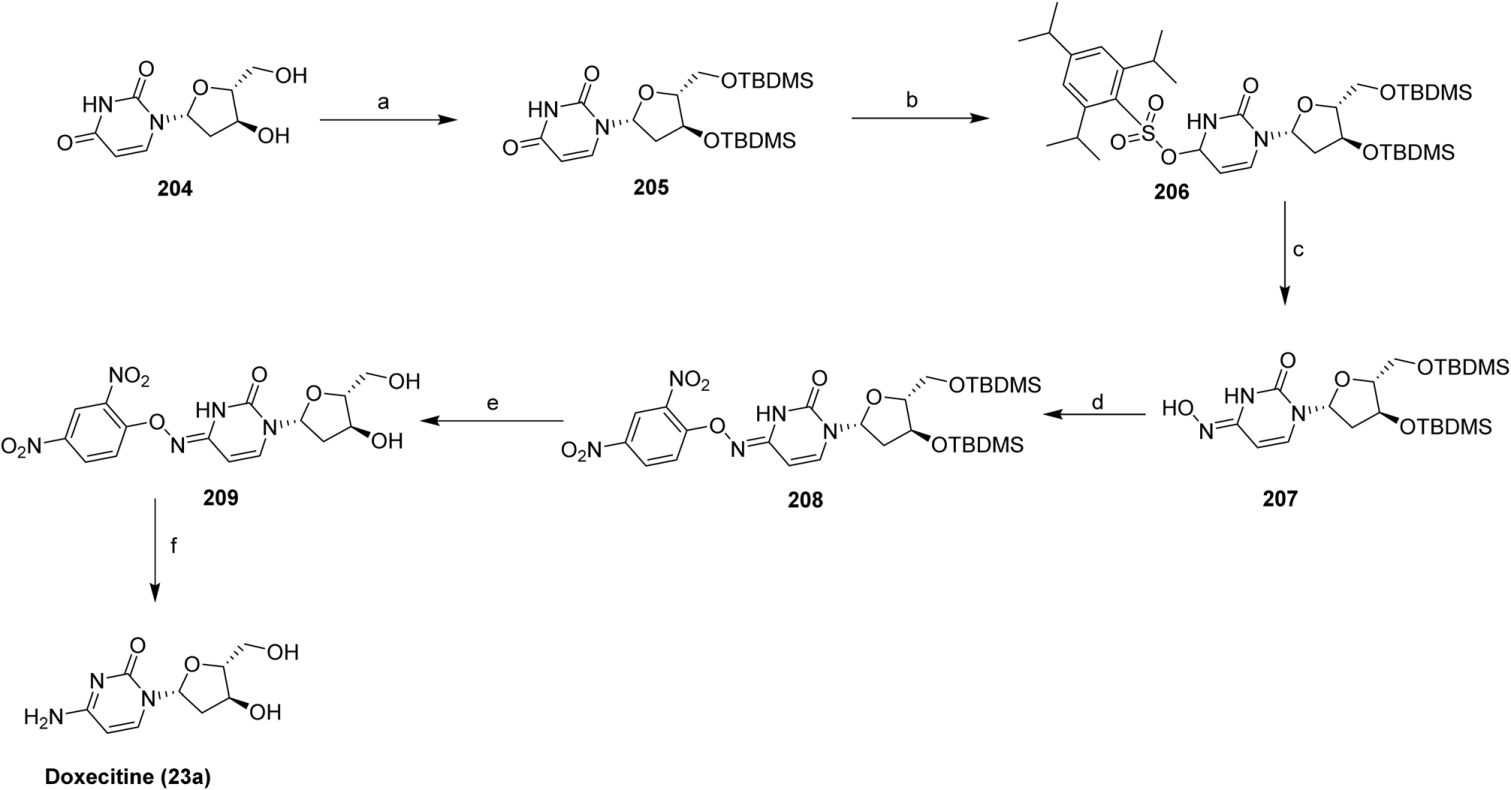
Synthesis of doxecitine (23a): ^*a*^Reaction conditions: (a) TBDMSCl, DMF, Py, Imidazole, 0 °C to rt, 15 h; (b) NaH, 2,4,6-triisopropylbenzenesulfonyl chloride, THF, 0 °C to rt, 63 h; (c) hydroxylamine hydrochloride, DBU, DMF, rt, 24 h; (d) 1-fluoro-2,4-dinitrobenzene, NaH, THF, 0 °C to rt, 1 h and 60 °C, 4 h; (e) triethylamine trishydrofluoride, THF, rt, overnight; (f) DMSO, MeOH, pH 7.2, rt, 0.5 h.

The synthesis of doxecitine (23a) and related compounds has been reported in the literature.^112,113^ As shown in Scheme 35, doxecitine (23a) was synthesized *via* a sequential protection, activation, and substitution strategy starting from the nucleoside deoxyuridine (204). Initial silylation of the ribose hydroxyl groups with TBDMSCl in DMF/pyridine in the presence of imidazole afforded intermediate 205, which was then converted to sulfonate 206 by treatment with NaH and 2,4,6-triisopropylbenzenesulfonyl chloride in THF. Displacement of the sulfonate with hydroxylamine hydrochloride under DBU-mediated conditions yielded intermediate 207, which was then subjected to nucleophilic aromatic substitution with 1-fluoro-2,4-dinitrobenzene to produce compound 208. Subsequent removal of the silyl protecting groups using triethylamine trihydrofluoride in THF afforded diol 209, and the final transformation under mild DMSO/MeOH conditions at near-neutral pH provided the target compound doxecitine (23a).

**Scheme 36:**
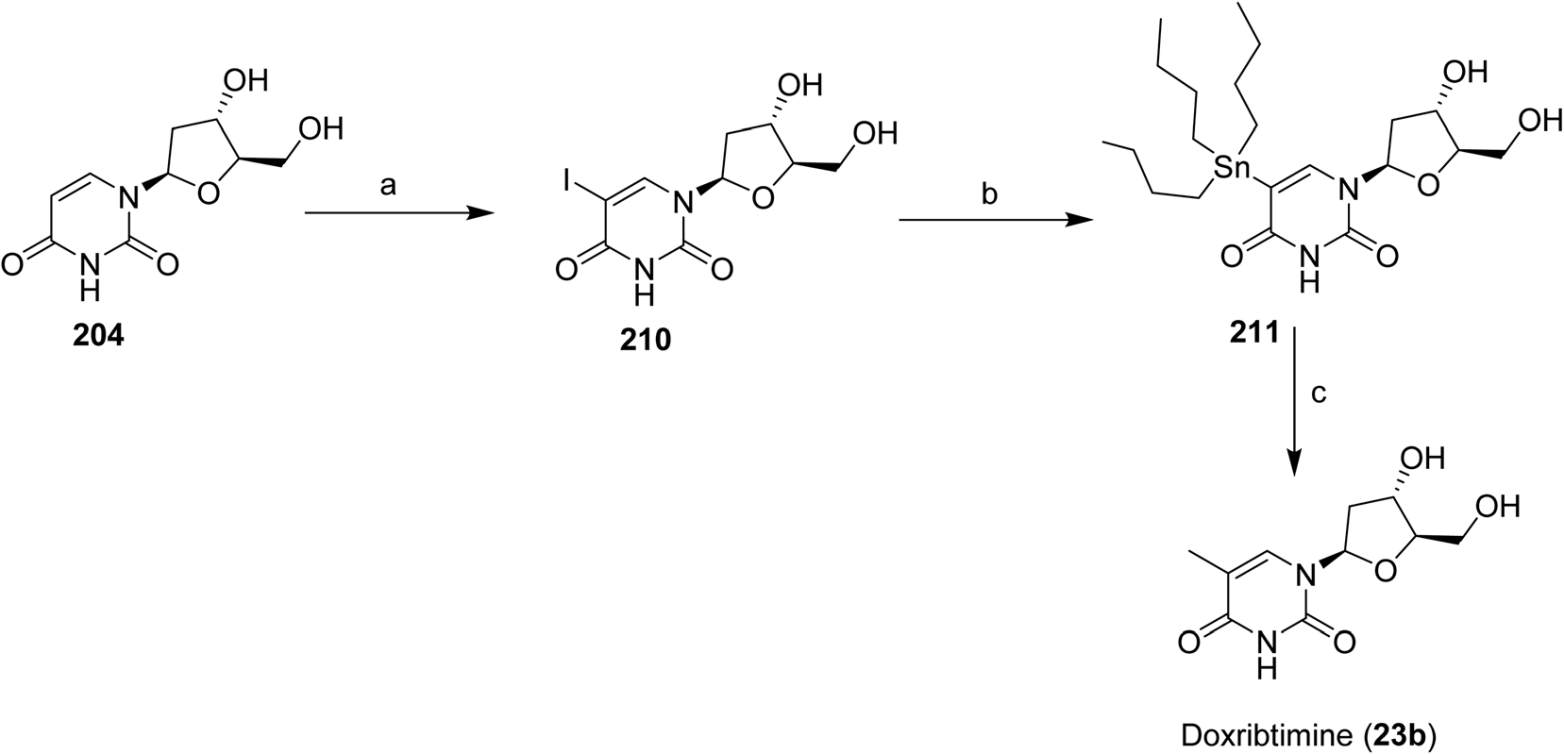
Synthesis of doxribtimine (23b): ^*a*^Reaction conditions: (a) iodine, silver nitrate, MeOH, 40 °C, 3 h; (b) Bis(tributyltin), tetrakis(triphenylphosphine)palladium, DMF, 65 °C, 5 h; (c) methyl iodide, tris(dibenzylideneacetone)dipalladium, tri(*o*-tolyl)phosphine, copper bromide, cesium fluoride, DMF, 5 minutes at rt, and then 5 minutes at 60 °C.

Similarly, compound doxribtimine (23b)^114,115^ was synthesized from nucleoside 204*via* halogenation, an organometallic transformation, and a final coupling reaction to afford the desired product (Scheme 35). Treatment of 204 with iodine and silver nitrate in MeOH at 40 °C afforded the iodinated intermediate 210, which was then converted to the organotin derivative 211 by reaction with bis(tributyltin) in the presence of tetrakis(triphenylphosphine)palladium in DMF. Final conversion of 211 using methyl iodide, tris(dibenzylideneacetone)dipalladium, tri(*o*-tolyl)phosphine, copper bromide, and cesium fluoride in DMF under controlled heating conditions afforded the target compound doxribtimine (23b) (Scheme 36).

#### 2.2.15 Nuzolvence™ (zoliflodacin)

Nuzolvence™ (zoliflodacin, 24) developed by Innoviva Specialty Therapeutics, Inc., is a first-in-class spiropyrimidinetrione antibiotic, indicated for the treatment of uncomplicated urogenital gonorrhea caused by *Neisseria gonorrhoeae* in adults and pediatric patients 12 years of age and older and weighing at least 35 kg. Gonorrhea is a sexually transmitted infection caused by *Neisseria gonorrhoeae*. Uncomplicated urogenital gonorrhea involves localized infection of the urethra or cervix and commonly presents with painful urination, genital discharge, and swelling; if untreated, it may progress to reproductive tract infection and infertility. Zoliflodacin inhibits bacterial type II topoisomerases (DNA gyrase and topoisomerase IV), which are required for DNA synthesis. Unlike other topoisomerase inhibitors, zoliflodacin binds within the DNA–gyrase cleavage complex, stabilizing the cleaved DNA state and preventing religation. Structural studies indicate that this interaction primarily involves the GyrB subunit, with contacts to conserved residues such as Asp437, thereby conferring a mechanism distinct from fluoroquinolones and retaining activity against resistant strains.^116^ This distinct mode of action targeting the gyrase B subunit allows for activity against strains resistant to other classes of antimicrobials, including fluoroquinolones.^117,118^ FDA approval was based on the results from a Phase 3 clinical trial (NCT03959527; Trial 1). To limit antibacterial resistance, Nuzolvence should be used only for infections proven or strongly suspected to be bacterial.

**Scheme 37:**
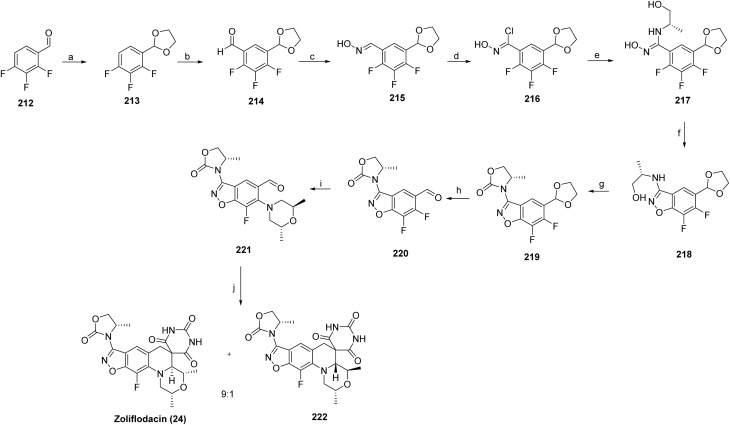
Synthesis of zoliflodacin (24). ^*a*^Reaction conditions: (a) ethylene glycol, *p*-TsOH, refluxing toluene, 78% yield; (b) *n*-BuLi, −70 °C, THF, DMF quench, 93% yield; (c) NH_2_OH, EtOH, rt, 24 h, 80% yield; (d) NCS, DMF, rt, 4 h, 76% yield; (e) d-alaninol, DMF, rt1-3 h; (f) Cs_2_CO_3_, rt, 59–93%; (g) CDI, DIEA, DMF, 70 °C, 2–3 h; (h) HCl, THF, water, 70 °C, 75–99%; (i) (2*R*,6*R*)-2,6-dimethylmorpholine, K_2_CO_3_ or DIEA, CH_3_CN, water, 80 °C, 4–5 h, 73–98%; (j) pyrimidine-2,4,6(1*H*,3*H*,5*H*)-trione, AcOH, water 120 °C, 1 h, 26–81%.

The synthesis was initiated by protecting the starting material (212) with ethylene glycol in the presence of *p*-TsOH in refluxing toluene to afford the corresponding acetal (213) lithiation with *n*-BuLi in THF at −70 °C, followed by DMF quenching, provided the formylated intermediate (214), which was subsequently converted to oxime using hydroxylamine in ethanol (215). Treatment with NCS in DMF furnished the chlorinated derivative (216), which was then reacted with d-alaninol in DMF to yield the corresponding amine intermediate (217). Further transformation with Cs_2_CO_3_ at room temperature afforded the coupled product (218), which was activated using CDI and DIEA in DMF at 70 °C (219). Acidic deprotection with HCl in THF/water gave the key intermediate, (220) which was then reacted with (2*R*,6*R*)-2,6-dimethylmorpholine in the presence of K_2_CO_3_ or DIEA in CH_3_CN/water at 80 °C (221). Finally, condensation with pyrimidine-2,4,6 (1*H*,3*H*,5*H*)-trione in AcOH/water at 120 °C afforded a 9 : 1 mixture of diastereomers, from which the major isomer was separated to furnish the target compound zoliflodacin (24) (Scheme 37).

#### 2.2.16 Myqorzo™ (aficamten)

Myqorzo™ (aficamten, 25) developed by cytokinetics, is an oral, selective, and reversible inhibitor of cardiac myosin designed to reduce hypercontractility and left ventricular outflow tract (LVOT) obstruction, key features of symptomatic obstructive hypertrophic cardiomyopathy (oHCM).^119–121^ At the molecular level, aficamten binds to an allosteric site in the cardiac myosin motor domain, stabilizing a pre-powerstroke, weak actin-binding state. X-ray crystallographic studies demonstrate that this interaction suppresses phosphate release and prevents transition to force-generating conformations, thereby reducing myocardial contractility.^122^ The U.S. FDA approved Myqorzo for adults with symptomatic oHCM to improve functional capacity and alleviate symptoms such as shortness of breath, fatigue, and risk of life-threatening cardiac events. Approval included a Risk Evaluation and Mitigation Strategy (REMS) due to the potential for reduced left ventricular ejection fraction (LVEF) and systolic dysfunction. The efficacy and safety of Myqorzo were evaluated in a 24 weeks, randomized study of 282 adults with symptomatic oHCM. Treatment with Myqorzo increased exercise capacity, as measured by peak oxygen uptake, and 59% of participants showed improvement in physical activity limitations, based on the New York Heart Association classification, compared with 24% in the placebo group. Myqorzo's use is limited by its potential to cause heart failure, necessitating REMS monitoring and drug interaction precautions.

**Scheme 38:**
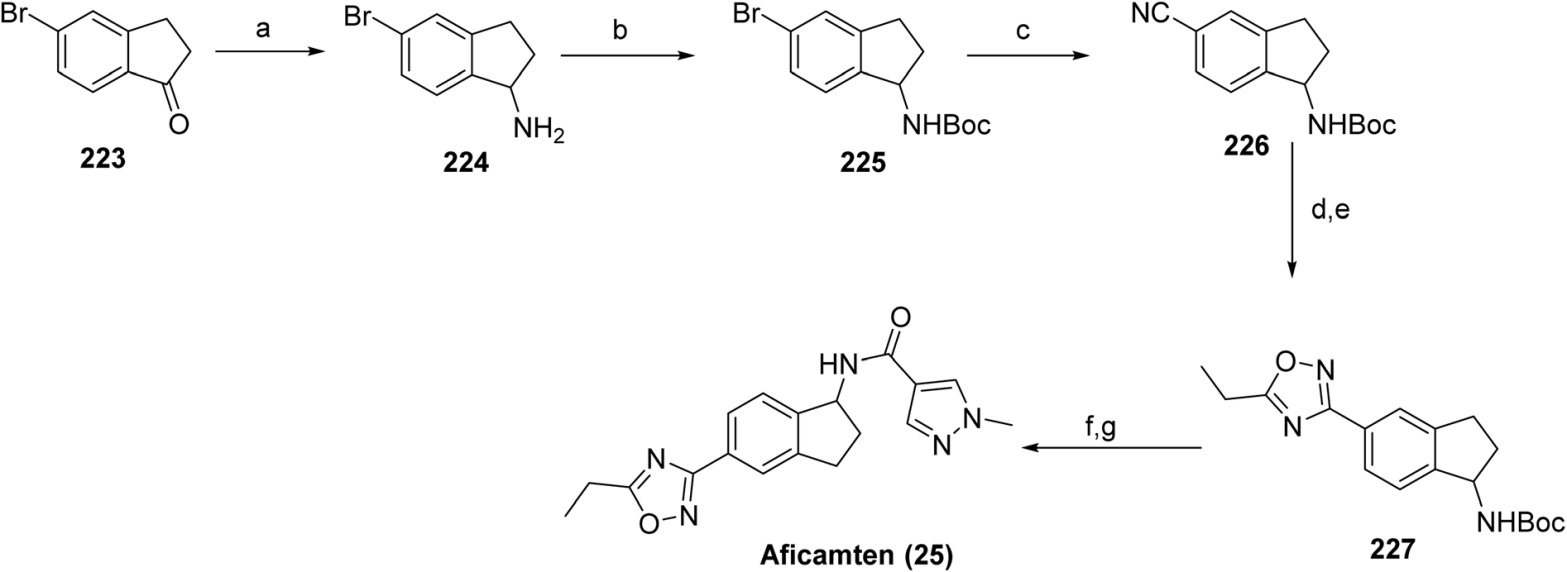
Synthesis of aficamten (25). ^*a*^Reaction conditions: (a) HCO_2_NH_4_, MeOH, NaBH_3_CN; (b) Boc_2_O, TEA, CHCl_2_; (c) K_4_Fe(CN)_6_·3H_2_O, XPhos Pd G2, KOAc, 1,4-dioxane/water; (d) H_2_NOH·HCl, TEA, EtOH; (e) ClCOCH_2_CH_3_, pyridine; (f) TFA, CH_2_Cl_2_; (g) 1-methyl-1*H*-pyrazole-4-carboxylic acid, EDC, HOBt, DIEA, DMF.

The synthesis of aficamten (25)^123^ was accomplished *via* a multistep route (Scheme 38). Reductive amination of 5-bromo-1-indanone (223) with ammonium formate and sodium cyanoborohydride furnished the corresponding amine (224), which was protected as the *tert*-butyloxycarbonyl (Boc) derivative. The resulting aryl bromide (225) underwent palladium-mediated cyanation using potassium ferrocyanide to afford the corresponding nitrile (226). Treatment of this nitrile with hydroxylamine generated a hydroxycarbamimidoyl intermediate (22), which is cyclized with propionyl chloride in the presence of pyridine to give the oxadiazole derivative. Finally, Boc deprotection of the amide followed by amide coupling with methyl pyrazole carboxylic acid furnished the target compound, aficamten (25).

#### 2.2.17 Nereus™ (tradipitant)

Nereus™ (tradipitant, 26), developed by Vanda Pharmaceuticals, is a selective, high-affinity antagonist of human substance P/NK-1 receptors. NK-1 receptors are expressed in the brainstem and the nucleus tractus solitarius, which integrate emetogenic signals from stimuli such as dizziness and vertigo.^124^ Tradipitant acts as a selective antagonist of the neurokinin-1 (NK1) receptor by binding to its orthosteric site and preventing activation by substance P, thereby suppressing downstream neurogenic signaling involved in emesis. The U.S. FDA approved tradipitant on December 30, 2025, for the prevention of motion-induced vomiting, marking the first new pharmacologic treatment for motion sickness in over four decades and representing a significant advancement in the management of this debilitating condition that affects a substantial portion of the population and has implications for military operational readiness. Its approval was supported by two Phase 3 real-world provocation studies conducted on boats. In Motion Syros (*n* = 365), vomiting incidence was 18.3–19.5% with NEREUS *versus* 44.3% with placebo (*p* < 0.0001), while in Motion Serifos (*n* = 316), vomiting rates were 10.4–18.3% with NEREUS *versus* 37.7% with placebo (*p* ≤ 0.0014), demonstrating risk reductions of 50–70%. Tradipitant has also been investigated in additional indications, including gastroparesis, COVID-19–associated pneumonia, and atopic dermatitis.^125,126^

**Scheme 39:**
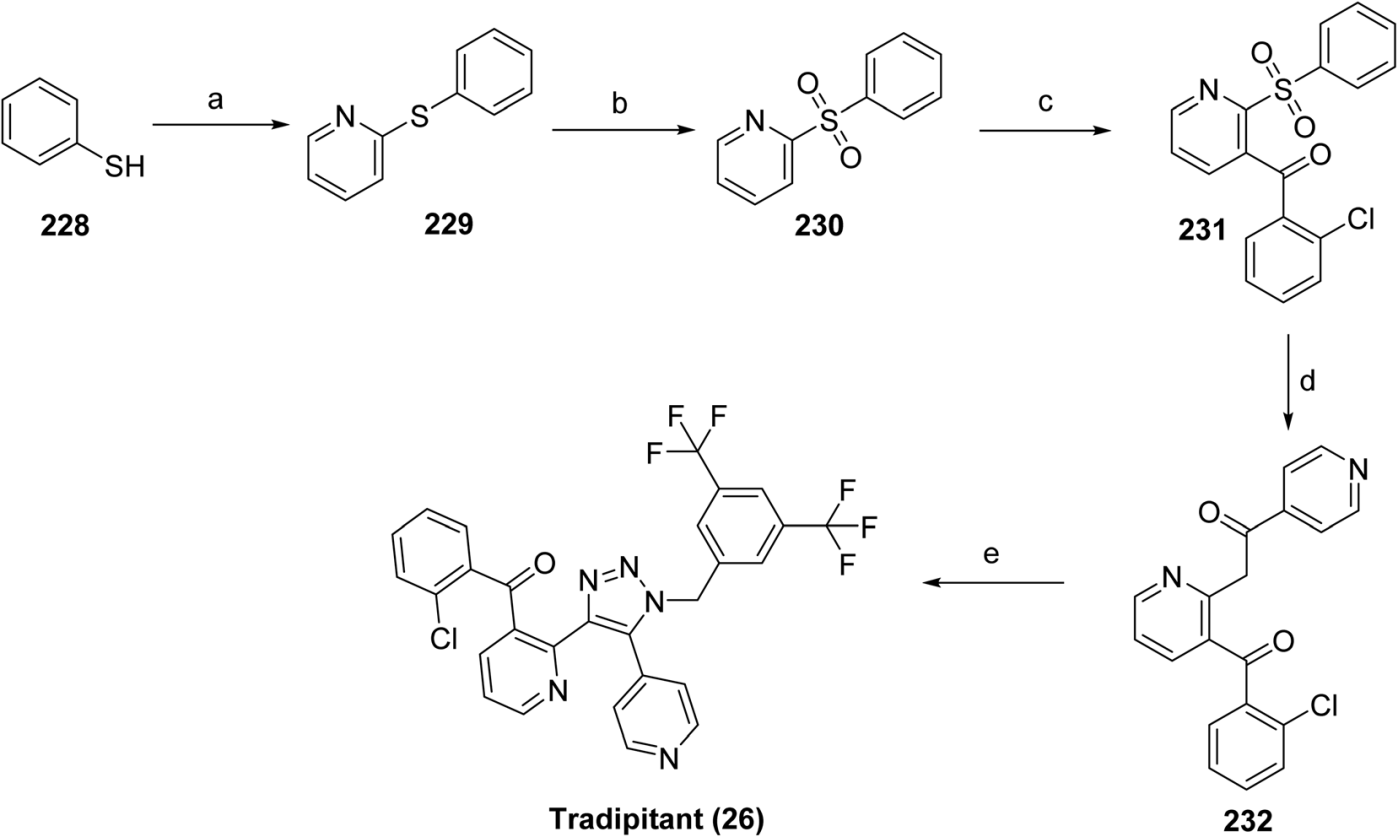
Synthesis of tradipitant (26). ^*a*^Reaction conditions: (a) K_2_CO_3_, DMF at 110 °C; (b) acetic acid, sodium hypochlorite, DMF, 45 °C, 45 min; (c) *n*-BuLi, i-Pr_2_NH, 2-chlorobenzaldehyde, NaOCl, TEMPO; (d) 4-acetylpyridine, *t*-BuOK, DMSO, LiOH, PhCOOH, iPrOAc; (e) 3,5-bis(trifluoromethyl)benzyl azide, K_2_CO_3_, *t*-BuOH, 1 h at rt, then reflux for 18 h.

Tradipitant was synthesized as shown in Scheme 39. The synthesis commences with a nucleophilic aromatic substitution of 2-chloropyridine with thiophenol (228) under basic conditions, affording the corresponding pyridyl sulfide (229). Subsequent oxidation of the sulfide furnishes the sulfone intermediate, 2-(benzenesulfonyl)pyridine (230). Directed lithiation of sulfone 230 using *n*-butyllithium in the presence of diisopropylamine, followed by electrophilic trapping with 2-chlorobenzaldehyde, yields ketone intermediate 231. This intermediate undergoes condensation with the enolate derived from 4-acetylpyridine, generated *in situ* with potassium *tert*-butoxide in DMSO. The resulting adduct is subjected to base-mediated cyclization in the presence of lithium hydroxide and benzoic acid to produce the pyridine benzoate intermediate 232. Finally, nucleophilic substitution with 3,5-bis(trifluoromethyl)benzyl azide affords the target molecule, tradipitant (26).

To provide a comparative overview of pharmacological profiles, Table 1 summarizes representative activity data (IC_50_/EC_50_ or equivalent potency values) for selected FDA-approved drugs against their primary biological targets. It should be noted that standardized IC_50_ and CC_50_ values are not consistently reported, as pharmacological evaluations often involve different assay systems and endpoints. Therefore, representative potency data have been included where available to ensure accuracy and consistency, offering a clear overview of their pharmacological characteristics.

**Table 1:** Representative activity and target profiles of nitrogen-containing FDA-approved drugs in 2025

S. no	Brand name	Active ingredient	Activity and target	References
1	Romvimza™	Vimseltinib	IC_50_: <0.01 µM (c-FMS/CSF-IR), 0.1–1 µM (c-Kit)	127
2	Avmapki Fakzynja Co-Pack™	Avutometinib and defactinib	Avutometinib: (IC_50_): MEK 160 nM; BRafV600E 8.2 nM; Braf 190 nM; CRAF 56 nM	128
			Defactinib: FAK (IC_50_): 0.6 nM	
3	Ibtrozi™	Taletrectinib	IC_50_: (1–1000 nM; 72 hours) against Ba/F3-TPM3-NTRK1, Ba/F3-ETV6-NTRK1, –NTRK2, –NTRK3, or KM12 cells is ∼3–20 nM	129
4	Zegfrovy™	Sunvozertinib	IC_50_: EGFR exon 20 insertion: 20.4 nM	130
			EGFRL858R/T790M: 1.1 nM	
			Her2 Exon20 YVMA: 7.5 nM	
5	Modeyso™	Dordaviprone	hERG IC_50_ is reported as 2.4 µM	131
6	Hernexeos™	Zongertinib	IC_50_: HER2 (YVMA) 16 nM; EGFR wild type 1540 nM	132
7	Inluriyo™	Imlunestrant	IC_50_: MCF7: 17 nM	133
8	Komzifti™	Ziftomenib	IC_50_: (4–31 nM) for KMT2A-rearranged and NPM1-mutant acute myeloid leukemia (AML) cell lines	59
9	Hyrnuo™	Sevabertinib	IC_50_: <0.5 nM for wild-type HER2, HER2 A775insYVMA, wild-type EGFR, and EGFR D770_N771insSVD.	62
10	Journavx™	Suzetrigine	Highly selective Na_V_1.8 inhibitor	65
11	Blujepa™	Gepotidacin	The MIC_50_ and MIC_90_ for gepotidacin against the 25 *N. gonorrhoeae* isolates tested are 0.12 and 0.25 µg mL^−1^, respectively	134
12	Vanrafia™	Atrasentan	IC_50_: 0.055 nM (ET_A_)	135
13	Ekterly™	Sebetralstat	IC_50_: 6.0 nM plasma kallikrein	81
14	Anzupgo™	Delgocitinib	IC_50_: JAK2:2.6 nM; JAK1:2.8 nM; JAK3:13 nM; Tyk2: 58 nM	136
15	Sephience™	Sepiapterin	Human endogenous metabolite; BH_4_ precursor (eNOS cofactor)	87
16	Vizz™	Aceclidine	Aceclidine is a modulator of M3 muscarinic acetylcholine receptor and a M1 receptor agonist (EC50: 40 µM)	137
17	Brinsupri™	Brensocatib	DPP1 inhibitor with pIC_50_s of 6.85, 7.6, 7.7, 7.8, and 7.8 in human, mouse, rat, dog and rabbit, respectively	138
18	Wayrilz™	Rilzabrutinib	IC_50_: BTK: 1.3 nM; BMX: 1.0 nM; ITK: 440 nM; TEC:0.8 nM *etc.*	138 and 139
19	Palsonify™	Paltusotine	Human SST2 EC_50_: 0.25 nM	99
			Rat SST2 EC_50_: 1.2 nM	
20	Rhapsido™	Remibrutinib	IC_50_: 1 nM (BTK)	103
21	Jascayd™	Nerandomilast	PDE4B IC_50_: 7.2 nM	140
22	Lynkuet™	Elinzanetant	Orally active and selective NK-1 and NK-3 receptor antagonist	108
23	Kygevvi™	Doxecitine and Doxribtimine	Nucleoside therapy; restores mtDNA in TK2 deficiency	141
24	Nuzolvence™	Zoliflodacin	*S. aureus* with the MIC_90_ of 0.25 µg mL^−1^; HepG2: IC_50_: > 50 µM	142
25	Myqorzo™	Aficamten	IC_50_ of 1.4 µM	123
26	Nereus™	Tradipitant	NK1 receptor antagonist; inhibits substance P-mediated signaling	125

### 2.3 Highlights of FDA-approved drugs in 2025

In Table 2, the key highlights of FDA-approved drugs in 2025 are summarized, including their active pharmaceutical ingredients, therapeutic indications, recommended dosages, and approval dates as reported by the U.S. FDA. The table emphasizes the structural diversity of nitrogen-containing motifs present in these agents and illustrates their contributions to target selectivity and overall therapeutic performance, underscoring the importance of nitrogen functionalities in contemporary drug discovery and design.

**Table 2:** Nitrogen-containing heterocyclic drugs approved by the FDA in 2025

S. no	Drug name	Active ingredient	FDA-approved use, dosage, and administration on approval date	FDA approval date
**I. Anticancer drugs**				
1	Romvimza™	Vimseltinib	Use: for the treatment of symptomatic TGCT for which surgical resection will potentially cause worsening functional limitation or severe morbidity	2/14/2025
			Recommended dosage and administration: 30 mg orally taken twice weekly, with a minimum of 72 hours between doses	
2	Avmapki Fakzynja Co-Pack™	Avutometinib and defactinib	Use: for the treatment of KRAS-mutated recurrent low-grade serous ovarian cancer (LGSOC) after prior systemic therapy	5/8/2025
			Recommended dosage and administration: AVMAPKI 3.2 mg administered orally twice weekly (day 1 and day 4) for the first 3 weeks of each 4 weeks cycle	
			FAKZYNJA 200 mg administered orally twice daily for the first 3 weeks of each 4 weeks cycle	
3	Ibtrozi™	Taletrectinib	Use: for the treatment of locally advanced or metastatic ROS1-positive non-small cell lung cancer	6/11/2025
			Recommended dosage and administration: 600 mg orally once daily on an empty stomach (no food intake at least 2 hours before and 2 hours after taking IBTROZI	
4	Zegfrovy™	Sunvozertinib	Use: for the treatment of locally advanced or metastatic non-small cell lung cancer with epidermal growth factor receptor exon 20 insertion mutations, as detected by an FDA-approved test, with disease progression on or after platinum-based chemotherapy	7/2/2025
			Recommended dosage and administration: 200 mg orally once daily taken with food	
5	Modeyso™	Dordaviprone	Use: to treat diffuse midline glioma harboring an H3 K27M mutation with progressive disease following prior therapy	8/6/2025
			Recommended dosage and administration: in pediatric patients weighing ≥10 kg is based on body weight	
			Taken orally once weekly on an empty stomach (at least 1 hour before or 3 hours after food intake)	
6	Hernexeos™	Zongertinib	Use: to treat adults with unresectable or metastatic non-squamous non-small cell lung cancer whose tumors have HER2 tyrosine kinase domain activating mutations, as detected by an FDA-approved test, and who have received prior systemic therapy	8/8/2025
			Recommended dosage and administration: it is based on body weight < 90 kg : 120 mg; ≥ 90 kg : 180 mg. Orally once daily with or without food until disease progression or unacceptable toxicity	
7	Inluriyo™	Imlunestrant	Use: for the treatment of estrogen receptor-positive, human epidermal growth factor receptor 2-negative, estrogen receptor-1-mutated advanced or metastatic breast cancer with disease progression following at least one line of endocrine therapy	9/25/2025
			Recommended dosage and administration: 400 mg orally once daily, on an empty stomach	
8	Komzifti™	Ziftomenib	Use: for the treatment of adults with relapsed or refractory acute myeloid leukemia with a susceptible nucleophosmin 1 mutation who have no satisfactory alternative treatment options	11/13/2025
			Recommended dosage and administration: 600 mg taken orally once daily until disease progression or unacceptable toxicity	
9	Hyrnuo™	Sevabertinib	Use: for the treatment of locally advanced or metastatic non-squamous non-small cell lung cancer with tumors that have activating HER2 tyrosine kinase domain activating mutations in patients who received a systemic therapy	11/19/2025
			Recommended dosage and administration: 20 mg orally twice daily with food until disease progression or unacceptable toxicity	

**II. Non-anticancerous drugs**				
10	Journavx™	Suzetrigine	Use: for the treatment of moderate to severe acute pain	1/30/2025
			Recommended dosage and administration: it is 100 mg. Take the starting dose on an empty stomach at least 1 hour before or 2 hours after food	
11	Blujepa™	Gepotidacin	Use: to treat uncomplicated urinary tract infections	3/25/2025
			Recommended dosage and administration: 1500 mg (two 750 mg tablets) orally twice daily after meals, approximately 12 hours apart, for 5 days	
12	Vanrafia™	Atrasentan	Use: to reduce proteinuria in adults with primary immunoglobulin A nephropathy at risk of rapid disease progression	4/02/2025
			Recommended dosage and administration: 0.75 mg orally once daily with or without food	
13	Ekterly™	Sebetralstat	Use: to treat acute attacks of hereditary angioedema	7/3/2025
			Recommended dosage and administration: one dose of 600 mg (2 tablets) taken orally at the earliest recognition of an HAE attack	
14	Anzupgo™	Delgocitinib	Use: to treat moderate-to-severe chronic hand eczema when topical corticosteroids are not advisable or produce an inadequate response	7/23/2025
			Recommended dosage and administration: apply to affected areas of the hands and wrists twice daily. Not more than 30 g per 2 weeks or 60 g per month. For topical use only	
15	Sephience™	Sepiapterin	Use: to treat hyperphenylalaninemia in patients with sepiapterin-responsive phenylketonuria, in conjunction with a phenylalanine-restricted diet.	7/28/2025
			Recommended dosage and administration: it is taken orally once daily with food. The starting dosage is	
			Less than 6 months 7.5 mg kg^−1^	
			6 months to less than 1 year 15 mg kg^−1^	
			1 year to less than 2 years 30 mg kg^−1^	
			2 years and older 60 mg kg^−1^	
16	Vizz™	Aceclidine	Use: to treat presbyopia	7/31/2025
			Recommended dosage and administration: instill one drop in each eye, wait 2 minutes, and instill a second drop in each eye once daily	
17	Brinsupri™	Brensocatib	Use: to treat non-cystic fibrosis bronchiectasis	8/12/2025
			Recommended dosage and administration: 10 mg or 25 mg orally once daily with or without food	
18	Wayrilz™	Rilzabrutinib	Use: to treat persistent or chronic immune thrombocytopenia that has not sufficiently responded to immunoglobulins, anti-D therapy, or corticosteroids	8/29/2025
			Recommended dosage and administration: 400 mg orally twice daily; swallow whole with water, with or without food. Do not cut, crush, or chew tablets	
19	Palsonify™	Paltusotine	Use: to treat acromegaly in adults who had an inadequate response to surgery and/or for whom surgery is not an option	9/25/2025
			Recommended dosage and administration: orally once daily with water on an empty stomach (at least 6 hours after a meal) and at least 1 hour before the next meal. Initial dose of 40 mg once daily; may reduce to 20 mg temporarily for tolerability, then resume 40 mg	
20	Rhapsido™	Remibrutinib	Use: to treat chronic spontaneous urticaria in adults who remain symptomatic despite H1 antihistamine treatment	9/30/2025
			Recommended dosage and administration: it is taken 25 mg orally twice daily with or without food	
			Swallow tablets whole. Do not split, crush, or chew	
21	Jascayd™	Nerandomilast	Use: to treat idiopathic pulmonary fibrosis	10/7/2025
			Recommended dosage and administration: 18 mg orally twice daily approximately 12 hours apart with or without food	
22	Lynkuet™	Elinzanetant	Use: to treat moderate-to-severe vasomotor symptoms due to menopause	10/24/2025
			Recommended dosage and administration: 120 mg (two 60 mg capsules) orally once daily at bedtime with or without food. Swallow tablets whole. Do not split, crush, or chew	
23	Kygevvi™	Doxecitine and doxribtimine	Use: to treat thymidine kinase 2 deficiency in patients who start to show symptoms when they are 12 years old or younger	11/3/2025
			Recommended dosage and administration: starting 260 mg kg^−1^ day^−1^ (consisting of 130 mg doxecitine and 130 mg doxribtimine), intermediate 520 mg kg^−1^ day^−1^ (consisting of 260 mg doxecitine and 260 mg doxribtimine), maintenance 800 mg kg^−1^ day^−1^ (consisting of 400 mg doxecitine and 400 mg doxribtimine)	
24	Nuzolvence™	Zoliflodacin	Use: to treat uncomplicated urogenital gonorrhea due to *Neisseria gonorrhoeae*	12/12/2025
			Recommended dosage and administration: adults and pediatric patients 12 years of age and older, weighing at least 35 kg: recommended dose is 3 g (one packet) administered as a single dose orally	
25	Myqorzo™	Aficamten	Use: to treat symptomatic obstructive hypertrophic cardiomyopathy	12/19/2025
			Recommended dosage and administration: starting dose is 5 mg orally once daily	
26	Nereus™	Tradipitant	Use: to treat vomiting associated with motion	12/30/2025
			Recommended dosage and administration: 5 mg or 170 mg orally, single dose, ∼60 minutes before motion, on an empty stomach	

## 3. Conclusion

The analysis of FDA-approved drugs in 2025 shows a strong dominance of nitrogen-containing heterocyclic small molecules, reaffirming their central role in modern medicinal chemistry. Of the 46 drugs approved in 2025, 26 (∼56.5%) are nitrogen-containing small molecules, the largest chemical category, compared with monoclonal antibodies/biologics (14; ∼30.4%), non-nitrogen small molecules (3; ∼6.5%), and oligonucleotide/ASO/siRNA therapeutics (3; ∼6.5%). This trend underscores the sustained preference for nitrogen-rich scaffolds in the design of clinically effective and drug-like molecules.

A defining structural characteristic of these nitrogen-containing drugs is the broad use of heterocyclic frameworks, including pyridines, pyrimidines, imidazoles, triazoles, indoles, and fused polycyclic nitrogen systems, which enable precise tuning of electronic properties, hydrogen-bonding potential, solubility, metabolic stability, and receptor-binding affinity. In particular, oncology-focused approvals, such as Vimseltinib (1), Taletrectinib (3), Sunvozertinib (4), Dordaviprone (5), Zongertinib (6), Ziftomenib (8), and Sevabertinib (9), illustrate the strategic use of nitrogen heterocycles for kinase inhibition, mutation-selective targeting, and enhanced therapeutic precision. Similarly, CNS- and rare-disease-directed agents, including Sepiapterin (15), Doxecitine/Doxribtimine (23), and Tradipitant (26), highlight the role of nitrogen-centered scaffolds in improving brain penetration, pharmacokinetics, and pathway-specific modulation.

Beyond oncology, nitrogen-containing frameworks underpin advances in anti-infective, immunological, metabolic, cardiovascular, and pain-related therapies, exemplified by Gepotidacin (11), Zoliflodacin (24), Remibrutinib (20), Nerandomilast (21), Paltusotine (19), Aficamten (25), and Suzetrigine (10). A notable feature of these molecules is their increased stereochemical complexity and structural refinement, reflecting regulatory trends that prioritize enantioselectivity, safety, and target specificity, particularly in kinase inhibitors, immune modulators, and CNS-active drugs.

From a medicinal chemistry and synthetic perspective, the 2025 FDA approvals underscore the growing reliance on nitrogen heterocycles as privileged scaffolds in modern drug design. Advances in heterocycle-focused synthetic methodologies, including late-stage functionalization, modular cross-coupling, asymmetric synthesis, and scalable green chemistry, have enabled efficient construction of structurally complex and stereochemically rich nitrogen frameworks. Collectively, these trends underscore the indispensable role of N-heterocycles in modulating biological activity, optimizing pharmacokinetic profiles, and enhancing target engagement. As synthetic innovation and structure-guided design continue to evolve, nitrogen-containing scaffolds are poised to remain key drivers of next-generation therapeutic discovery.

## Conflicts of interest

There are no conflicts of interest to declare.

## Data Availability

No primary research results, software, or code have been included, and no new data were generated or analyzed as part of this review.
